# Genetic susceptibility to bone and soft tissue sarcomas: a field synopsis and meta-analysis

**DOI:** 10.18632/oncotarget.24719

**Published:** 2018-04-06

**Authors:** Clara Benna, Andrea Simioni, Sandro Pasquali, Davide De Boni, Senthilkumar Rajendran, Giovanna Spiro, Chiara Colombo, Calogero Virgone, Steven G. DuBois, Alessandro Gronchi, Carlo Riccardo Rossi, Simone Mocellin

**Affiliations:** ^1^ Department of Surgery Oncology and Gastroenterology, University of Padova, Padova, Italy; ^2^ Clinica Chirurgica I, Azienda Ospedaliera Padova, Padova, Italy; ^3^ Surgical Oncology Unit, Istituto Oncologico Veneto (IOV-IRCCS), Padova, Italy; ^4^ Sarcoma Service, Department of Surgery, Fondazione IRCCS Istituto Nazionale dei Tumori, Milano, Italy; ^5^ Pediatric Surgery, Department of Women's and Children's Health, University of Padua, Padua, Italy; ^6^ Department of Pediatric Hematology/Oncology, Dana-Farber/Boston Children's Cancer and Blood Disorders Center and Harvard Medical School, Boston, MA, USA

**Keywords:** sarcoma, SNP, meta-analysis, polymorphisms, risk

## Abstract

**Background:**

The genetic architecture of bone and soft tissue sarcomas susceptibility is yet to be elucidated. We aimed to comprehensively collect and meta-analyze the current knowledge on genetic susceptibility in these rare tumors.

**Methods:**

We conducted a systematic review and meta-analysis of the evidence on the association between DNA variation and risk of developing sarcomas through searching PubMed, The Cochrane Library, Scopus and Web of Science databases. To evaluate result credibility, summary evidence was graded according to the Venice criteria and false positive report probability (FPRP) was calculated to further validate result noteworthiness. Integrative analysis of genetic and eQTL (expression quantitative trait locus) data was coupled with network and pathway analysis to explore the hypothesis that specific cell functions are involved in sarcoma predisposition.

**Results:**

We retrieved 90 eligible studies comprising 47,796 subjects (cases: 14,358, 30%) and investigating 1,126 polymorphisms involving 320 distinct genes. Meta-analysis identified 55 single nucleotide polymorphisms (SNPs) significantly associated with disease risk with a high (N=9), moderate (N=38) and low (N=8) level of evidence, findings being classified as noteworthy basically only when the level of evidence was high. The estimated joint population attributable risk for three independent SNPs (rs11599754 of *ZNF365/EGR2*, rs231775 of *CTLA4*, and rs454006 of *PRKCG*) was 37.2%. We also identified 53 SNPs significantly associated with sarcoma risk based on single studies.

Pathway analysis enabled us to propose that sarcoma predisposition might be linked especially to germline variation of genes whose products are involved in the function of the DNA repair machinery.

**Conclusions:**

We built the first knowledgebase on the evidence linking DNA variation to sarcomas susceptibility, which can be used to generate mechanistic hypotheses and inform future studies in this field of oncology.

## INTRODUCTION

Sarcomas are a family of rare malignant tumors arising from bone and soft tissues with more than 50 different histologies accounting for about 1-2% of cancers in adults and 15-20% in children (worldwide incidence: approximately 200,000 cases per year). The pathogenesis of sarcomas is multifactorial including environmental (such as exposure to ionizing radiations or chemical carcinogens) and genetic components, although the disease rarity represents an objective hurdle to the research in this field of investigation. Significant advances have been made in the understanding of the acquired genetic events leading to sarcomagenesis. It has been recognized that three types of somatic DNA alterations, translocations, mutations, and copy number variations, play a key role in these tumors [1]. As a consequence, sarcomas are grouped into two categories: balanced translocation associated sarcomas (BATS) and complex genotype/karyotype sarcomas (CGKS), which are characterized by a stable genome and genomic instability, respectively [2]. A potential therapeutic implication of such genetic taxonomy classification is that some recurrent chromosomal translocations might be exploited for the development of drugs targeting the protein products of fusion oncogenes [1].

Conversely, knowledge on the role of germline DNA variations in sarcomagenesis is sparse and limited. Although a minority of sarcomas arise within well characterized heritable cancer predisposition syndromes (e.g., osteosarcoma and Bloom syndrome, desmoid tumors and familial adenomatous polyposis) [3], the vast majority of sarcomas occur sporadically and the role of the genetic background in their pathogenesis is to be uncovered. Recent advances in molecular high-throughput technology, which conduct of genome wide association studies (GWAS), is accelerating the pace of discovery of sarcoma predisposition loci.

Looking at the already existing international literature, some investigators have meta-analyzed the evidence regarding a handful of SNPs such as *XRCC3* rs861539 [4], *MDM2* rs2279744 [5, 6], and *CTLA4* rs231775 [7]: however, to the best of our knowledge no comprehensive collection of the available data in this field of oncology has been published thus far.

With the present work we systematically reviewed and meta-analyzed the available evidence in this field in order to: 1) provide readers with the first knowledgebase dedicated to the relationship between germline DNA variation and sarcoma risk; 2) identify areas lacking of meaningful information thus helping to inform future studies; and 3) suggest a biological interpretation of current findings utilizing network and pathway analysis [8] after integrating multiple sources of biological data [9].

## RESULTS

### Characteristics of the eligible studies

**Figure 1:**
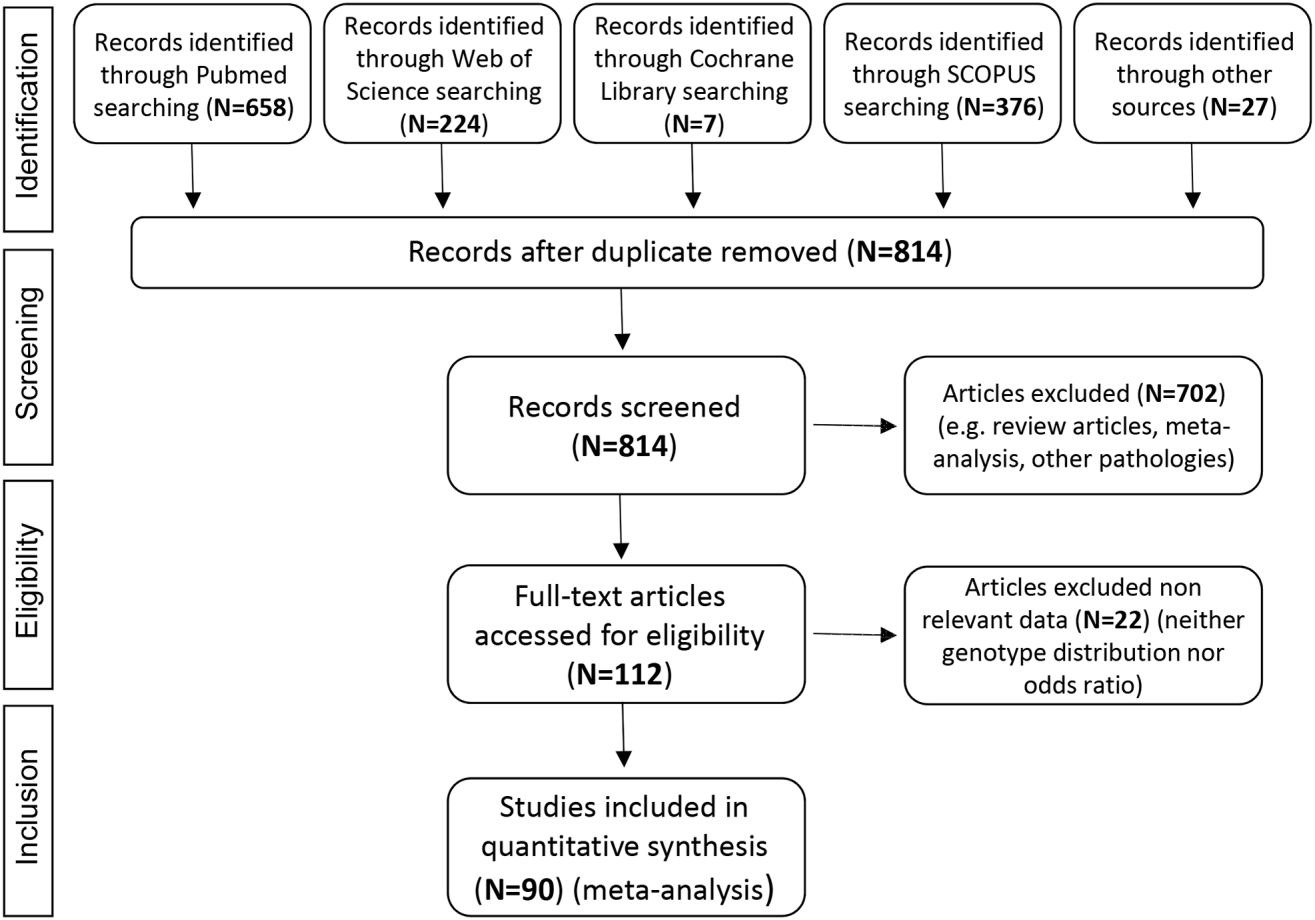
Flow diagram summarizing the search strategy and the study selection process

We identified 90 eligible articles, comprising 47,796 subjects, 14,358 cases and 33,438 controls. The details of the literature search are summarized in Figure 1.

Based on the prevalent ancestry (ie. the race of at least 80% of the enrolled subjects) the majority of the studies were Asian (N=57 studies) the rest being Caucasian (N=25 studies), or mixed (N=8 studies). Based on study design, half of included studies were population based case-controls studies (N=40 studies), the remaining were hospital based (N=39 studies), with a few (N=11) being mixed or not specified. Two studies were GWAS [10, 11].

According to histology, the majority of the eligible studies investigated bone tumors (N=65) and the remaining investigated Ewing's sarcoma (N=9), soft tissue sarcomas (N=6), chordoma (N=4), hemangiosarcoma (N=1), and mixed sarcomas (N=5). Thirteen studies investigated pediatric subjects or young adults. Although pediatric/young age ranged from 0 to 35 years old in eligible studies, most of the studies considered subjects < 20 years old.

We evaluated the included studies following the criteria of the Newcastle-Ottawa scale (NOS) scoring system. The mean score was 7.8. The main features of all the eligible studies and the NOS score are available on Table 1.

**Table 1:** Characteristics of the included studies and Newcastle-Ottawa quality assessment (NOS) evaluation

Included articles references			Subjects characteristics						NOS	
First Author	Journal	Year	Cancer Type	Cases	Controls	Age	Ethnicity	Source of Controls	NOS 123	NOS [0–9]
Adiguzel M. [12]	Indian J Exp Biol	2016	Bone tumors	54	81	Adult	Caucasian	Population	413	8
Alhopuro P. [13]	J Med Genet	2005	Soft tissue sarcoma	68	185	Adult	Caucasian	Population	413	8
Almeida PSR. [14]	Genet Mol Res	2008	Soft tissue sarcoma	100	85	Adult	Mixed	not specified	213	6
Aoyama T. [15]	Cancer Letters	2002	Bone tumors	38	72	Adult	Asian	Population	313	7
Barnette P. [16]	Cancer Epidemiol Biomarkers Prev	2004	Mixed	42	326	Pediat/Young	Caucasian	Population	323	8
Biason P. [17]	Pharmacogenomics J	2012	Bone tumors	130	250	Adult	Caucasian	Hospital	323	8
Bilbao-Aldaiturriaga N. [18]	Pediatr Blood Cancer	2015	Bone tumors	99	387	Pediat/Young	Caucasian	Hospital	323	8
Chen Y. [19]	Tumor Biol	2016	Bone tumors	190	190	Adult	Asian	Hospital	323	8
Cong Y. [20]	Tumor Biol	2015	Bone tumors	203	406	Adult	Asian	Hospital	323	8
Cui Y. [21]	Biomarkers	2016	Bone tumors	251	251	Adult	Asian	Hospital	323	8
Cui Y. [22]	Tumor Biol	2016	Bone tumors	260	260	Adult	Asian	Hospital	323	8
Dong YZ. [23]	Genet Mol Res	2015	Bone tumors	185	201	Adult	Asian	Hospital	323	8
DuBois SG. [24]	Pediatr Blood Cancer	2011	Ewing's sarcoma	135	200	Pediat/Young	Caucasian	Hospital	213	6
Ergen A. [25]	Mol Biol Rep	2011	Bone tumors	50	50	Adult	Caucasian	not specified	313	7
Feng D. [26]	Genet Test Mol Biomarkers	2013	Ewing's sarcoma	308	362	Adult	Asian	Hospital	323	8
Gloudemans T. [27]	Cancer Res	1993	Soft tissue sarcoma	9	26	Adult	Caucasian	Population	303	6
Grochola LF. [28]	Clin Cancer Res	2009	Soft tissue sarcoma	130	497	Adult	Caucasian	Population	313	7
Grünewald TG. [29]	Nat Genet	2015	Ewing's sarcoma	343	251	Adult	Caucasian	Population	423	9
Guo J. [30]	Genet Mol Res	2015	Bone tumors	136	136	Adult	Asian	Hospital	313	7
He J. [31]	Endocr J	2013	Bone tumors	415	431	Adult	Asian	Hospital	323	8
He J. [32]	Endocrine	2014	Bone tumors	415	431	Adult	Asian	Hospital	323	8
He M. [33]	Tumor Biol	2014	Bone tumors	189	195	Adult	Asian	Hospital	323	8
He ML. [34]	Asian Pac J Cancer Prev	2013	Bone tumors	59	63	Adult	Asian	Hospital	313	7
He Y. [35]	Int Orthop	2014	Bone tumors	120	120	Adult	Asian	Hospital	323	8
Hu GL. [36]	Genet Mol Res	2015	Bone tumors	130	130	Adult	Asian	Hospital	323	8
Hu YS. [37]	BMC Cancer	2010	Bone tumors	168	168	Adult	Asian	Population	423	9
Hu YS. [38]	Med Oncol	2011	Bone tumors	168	168	Adult	Asian	Population	423	9
Hu Z. [39]	Genet Test Mol Biomarkers	2015	Bone tumors	368	370	Adult	Asian	not specified	213	6
Ito M. [40]	Clin Cancer Res	2010	Soft tissue sarcoma	155	37	Adult	Mixed	Hospital	203	5
Jiang C. [41]	Med Oncol	2014	Bone tumors	168	216	Adult	Asian	Hospital	323	8
Kelley MJ. [42]	Hum Genet	2014	Chordoma	103	160	Adult	Asian	Population	413	8
Koshkina NV. [43]	J Pediatr Hematol Oncol	2007	Bone tumors	123	510	Pediat/Young	Mixed	Population	413	8
Le Morvan V. [44]	Int J Cancer	2006	Mixed	93	53	Adult	Caucasian	Population	403	7
Li L. [45]	Genet Mol Res	2015	Bone tumors	52	100	Adult	Asian	Hospital	312	6
Liu Y. [46]	DNA Cell Biol	2011	Bone tumors	267	282	Adult	Asian	Population	313	7
Liu Y. [47]	PloSONE	2012	Bone tumors	326	433	Adult	Asian	Population	423	9
Lu H. [48]	Tumor Biol	2015	Bone tumors	388	388	Adult	Asian	Hospital	323	8
Lu XF. [49]	Asian Pac J Cancer Prev	2011	Bone tumors	110	226	Adult	Asian	Hospital	313	7
Lv H. [50]	Mol Med Rep	2014	Bone tumors	103	201	Adult	Asian	Hospital	213	6
Ma X. [51]	Genet Mol Res	2016	Bone tumors	141	282	Adult	Asian	Hospital	223	7
Martinelli M. [52]	Oncotarget	2016	Ewing's sarcoma	100	147	Pediat/Young	Caucasian	Population	423	9
Mei JW. [99]	Int J Clin Exp Pathol	2016	Bone tumors	97	120	Adult	Asian	Population	313	7
Miao C.[53]	Sci Rep	2015	Soft tissue sarcoma	138	131	Adult	Asian	Hospital	223	7
Mirabello L. [54]	Carcinogenesis	2010	Bone tumors	99	1430	Adult	Caucasian	mixed	323	8
Mirabello L. [55]	BMC Cancer	2011	Bone tumors	96	1426	Adult	Caucasian	mixed	323	8
Nakayama R. [56]	Cancer Sci	2008	Mixed	544	1378	Adult	Asian	mixed	323	8
Naumov VA. [57]	Bull Exp Biol Med	2012	Bone tumors	68	96	Adult	Caucasian	not specified	313	7
Oliveira ID. [58]	J Pediatr Hematol Oncol	2007	Bone tumors	80	160	Pediat/Young	Mixed	Hospital	323	8
Ozger H. [59]	Folia Biologica (Praha)	2008	Mixed	56	44	Adult	Caucasian	Population	403	7
Patino-Garcia A. [60]	J Med Genet	2000	Bone tumors	110	111	Pediat/Young	Caucasian	not specified	323	8
Pillay N. [61]	Nat Genet	2012	Chordoma	40	358	Adult	Caucasian	population	323	8
Postel-Vinay S. [10]	Nat Genet	2012	Ewing's sarcoma	401	4352	Adult	Caucasian	population	423	9
Qi Y. [62]	Tumor Biol	2016	Bone tumors	206	206	Adult	Asian	Hospital	323	8
Qu WR. [63]	Genetic Mol Res	2016	Bone tumors	153	252	Adult	Asian	Hospital	323	8
Ru JY. [64]	Int J Clin Exp Pathol	2015	Bone tumors	210	420	Adult	Asian	Hospital	323	8
Ruza E. [65]	J Pediatr Hematol Oncol	2003	Mixed	125	143	Pediat/Young	Caucasian	not specified	322	7
Saito T. [66]	Int J Cancer	2000	Hemangiosarcoma	22	84	Adult	Mixed	Population	213	6
Salinas-Souza C. [67]	Pharmacogenet Genomics	2010	Bone tumors	80	160	Pediat/Young	Mixed	Hospital	323	8
Savage SA. [68]	Cancer Epidemiol Biomarkers Prev	2007	Bone tumors	104	74	Pediat/Young	Caucasian	Hospital	213	6
Savage SA. [69]	Pediatr Blood Cancer	2007	Bone tumors	104	74	Pediat/Young	Caucasian	Hospital	213	6
Savage SA. [11]	Nat Genet	2013	Bone tumors	941	3291	Adult	Caucasian	Population	423	9
Shi ZW. [70]	Cancer Biomark	2016	Bone tumors	174	150	Adult	Asian	Hospital	313	7
Silva DS. [71]	Gene	2012	Ewing's sarcoma	24	200	Adult	Mixed	Population	323	8
Tang YJ. [72]	Medicine	2014	Bone tumors	160	250	Adult	Asian	Population	423	9
Thurow HS. [73]	Mol Biol Rep	2013	Ewing's sarcoma	24	91	Adult	Mixed	Population	323	8
Tian Q. [74]	Eur J Surg Oncol	2013	Bone tumors	133	133	Adult	Asian	Population	423	9
Tie Z. [75]	Int J Clin Exp Pathol	2014	Bone tumors	165	330	Adult	Asian	Population	423	9
Toffoli G. [76]	Clin Cancer Res	2009	Bone tumors	201	250	Adult	Caucasian	Population	423	9
Walsh KM. [77]	Carcinogenesis	2016	Bone tumors	660	6892	Pediat/Young	Caucasian	Population	423	9
Wang J. [78]	DNA Cell Biol	2012	Ewing's sarcoma	158	212	Adult	Asian	Population	323	8
Wang J. [79]	DNA Cell Biol	2013	Bone tumors	106	210	Adult	Asian	Population	323	8
Wang K. [80]	Biomed Rep	2014	Chordoma	65	65	Adult	Asian	Population	313	7
Wang K. [81]	Tumor Biol	2016	Bone tumors	126	168	Adult	Asian	Hospital	323	8
Wang W. [82]	DNA Cell Biol	2011	Bone tumors	205	216	Adult	Asian	Hospital	323	8
Wang W. [83]	Genet Test Mol Biomarkers	2011	Bone tumors	205	215	Adult	Asian	Hospital	323	8
Wang Z. [84]	Tumor Biol	2014	Bone tumors	330	342	Adult	Asian	Population	423	9
Wu Y. [85]	Tumor Biol	2015	Bone tumors	124	136	Adult	Asian	Hospital	323	8
Wu Z. [86]	Int J Mol Sci	2013	Chordoma	65	120	Adult	Asian	not specified	313	7
Xin DJ. [87]	Int J Clin Exp Pathol	2015	Bone tumors	90	100	Adult	Asian	Population	413	8
Xu H. [88]	Med Sci Monit	2016	Bone tumors	279	286	Pediat/Young	Asian	Hospital	323	8
Xu S. [89]	DNA Cell Biol	2014	Bone tumors	202	216	Adult	Asian	Population	423	9
Yang L. [90]	Int J Clin Exp Pathol	2015	Bone tumors	152	304	Adult	Asian	Population	423	9
Yang S. [91]	Genet Test Mol Biomarkers	2012	Ewing's sarcoma	223	302	Adult	Asian	Population	423	9
Yang W. [92]	Med Oncol	2014	Bone tumors	118	126	Adult	Asian	not specified	323	8
Zhang G. [93]	Genet Mol Res	2015	Bone tumors	180	360	Adult	Asian	Population	423	9
Zhang HF. [94]	Genet Mol Res	2015	Bone tumors	182	182	Adult	Asian	Population	423	9
Zhang N. [95]	Onco Targets Ther	2016	Bone tumors	276	286	Adult	Asian	Hospital	323	8
Zhang Y. [96]	Tumor Biol	2014	Bone tumors	610	610	Adult	Asian	Population	423	9
Zhao J. [97]	BioMed Res Int	2014	Bone tumors	247	428	Adult	Asian	Population	423	9
Zhi LQ. [98]	Tumor Biol	2014	Bone tumors	212	240	Adult	Asian	Hospital	323	8

NOS: Newcastle-Ottawa quality assessment scale evaluation (0-9). NOS1: selection of the study groups (0-4); NOS2: comparability of the groups (0-2); NOS3: ascertainment of the exposure or outcome (0-3).

### Characteristics of the retrieved genetic variants

Overall, data on 1,126 polymorphisms involving 320 genes were retrieved. Variations were mainly SNPs, only six being insertion/deletions of more than one nucleotide. Based on the number of different genetic variations studied, the 11 most studied genes were the following: *EGR2* (179 different SNPs), *ADO* (58 different SNPs), *ZNF365* (40 different SNPs), *TRAPPC9* (28 different SNPs), *CASC8* (23 different SNPs), *CD99* (20 different SNPs), *EWSR1* (16 different SNPs) *TP53, HSD17B2* (15 different SNPs each) and *UGT1A8, LOC107984012* (12 different SNPs each).

Thirty-seven of these genetic variants were located no more than 2kb upstream the relevant gene, ten no more than 500bp downstream the relevant gene, 493 in introns, 100 in exons (non-UTRs), 19 in the 3’-UTR, seven in the 5’-UTR. Moreover, 413 SNPs were located in intergenic regions more than 2kb upstream or more than 500 bp downstream the relevant gene and 41 in non-coding transcripts. Among the exonic SNPs, 63 had a missense functional effect, while 37 were synonymous. Detailed information on all SNPs is reported in Supplementary Table 1.

### Meta-analysis findings

At least two independent datasets were available for 51 genetic variations allowing us to perform 118 meta-analyses, 16 of them were histology-based meta-analysis on osteosarcoma and Ewing's sarcoma. Moreover, 13 sensitivity analysis were performed considering the ethnicity of the different datasets. The results of data meta-analyses are comprehensively reported in Supplementary Table 2. Polymorphism “rs” identifier, nucleotide change and amino acid change are reported in Supplementary Table 3.

The eight most studied genetic variants were the following: *TP53* rs1042522 (6 datasets), *VEGF* rs3025039 and *GSTM1* deletion (5 datasets each), *CTLA4* rs231775, *CTLA4* rs5742909, *MDM2* rs2279744, rs10434 *VEGF* and *GSTT1* deletion (4 datasets each).

The number of subject (cases plus controls) enrolled in the 118 meta-analyses ranged from 144 to 5,347 (median: 1,195). Based on the number of subjects, the 10 most studied genetic variants, all with 5,347 subjects, were the following: *EGR2* rs224292 and rs224278, *ADO* rs1848797 and rs1509966, *MDM2* rs1690916, *LOC107984012* rs9633562, rs944684 and rs6479860, *ZNF365* rs11599754 and rs10761660.

Of the 118 meta-analyses and 13 sensitivity analysis (131 total analyses) performed, 55 resulted to be statistically significant (P-value <0.05). The level of summary evidence, among the significant associations identified by meta-analysis, was high, intermediate, and low in 9, 38, and 8 analyses respectively. The most frequent single cause of non-high-quality level of evidence was between-study heterogeneity followed by the small sample size. Considering all statistically significant meta-analyses FPRP was optimal (<0.2) at least at the 10E3 level for 10/55 analysis, 9 of them with high level of summary evidence.

The details of significant associations are reported in Table 2.

**Table 2:** Meta-analysis results: genetic variants significantly associated with sarcoma risk

SNP ID	Genes	Analysis	Model	Sarcoma type	data sets	Meta-analysis Ethnicity	OR [95% CI]	I ^2^ %	P value	Cases	Controls	Ref/ Alt	Venice Criteria	FPRP (E-03)	Level of Evidence
rs11599754	ZNF365, ADO	primary	Per allele	Ewing's	2	Caucasian	1.48 [1.32, 1.66]	0	<0.00001	744	4603	T/C	AAA	Y	HIGH
rs1509966	ADO, EGR2	primary	Per allele	Ewing's	2	Caucasian	1.58 [1.42, 1.77]	0	<0.00001	744	4603	A/G	AAA	Y	HIGH
rs1848797	ADO, EGR2	primary	Per allele	Ewing's	2	Caucasian	1.57 [1.4, 1.77]	0	<0.00001	744	4603	G/A	AAA	Y	HIGH
rs224278	EGR2	primary	Per allele	Ewing's	2	Caucasian	1.73 [1.49, 2.02]	0	<0.00001	744	4603	T/C	AAA	Y	HIGH
rs9633562	EGR2, LOC107984012	primary	Per allele	Ewing's	2	Caucasian	1.46 [1.29, 1.65]	0	<0.00001	744	4603	A/C	AAA	Y	HIGH
rs10761660	ADO, EGR2	primary	Per allele	Ewing's	2	Caucasian	1.39 [1.21, 1.6]	0	<0.00001	744	4603	T/C	AAA	Y	HIGH
rs224292	ADO, EGR2	primary	Per allele	Ewing's	2	Caucasian	1.67 [1.42, 1.96]	0	<0.00001	744	4603	A/G	AAA	Y	HIGH
rs231775	CTLA4	primary	Per allele	Mixed	4	Asian	1.36 [1.2, 1.54]	0	<0.00001	1003	1162	G/A	AAA	Y	HIGH
rs454006	PRKCG	primary	Per allele	Osteo	2	Asian	1.35 [1.18, 1.54]	0	<0.0001	998	998	T/C	AAA	Y	HIGH
rs944684	LOC107984012	primary	Per allele	Ewing's	2	Caucasian	1.73 [1.4, 2.14]	49	<0.00001	744	4603	C/T	ABA	Y	INTERM
rs2305089	T	sensitivity	Per allele	Chordoma	2	Caucasian	3.91 [2.4, 6.38]	47	<0.00001	163	881	G/A	ABA	N	INTERM
rs1042522	TP53	primary	Dominant	Mixed	6	Mixed	0.67 [0.53, 0.84]	0	0.0007	788	950	G/C	AAA	N	INTERM
rs1042522	TP53	subgroup	Dominant	Osteo	3	Mixed	0.6 [0.43, 0.84]	15	0.002	509	737	G/C	AAA	N	INTERM
rs1129055	CD86	primary	Recessive	Mixed	2	Asian	0.6 [0.41, 0.88]	0	0.008	363	428	A/G	BAA	N	INTERM
rs11737764	NUDT6	primary	Dominant	Bone tumor	2	Caucasian	2.12 [1.34, 3.37]	0	0.001	164	1522	A/C	AAA	N	INTERM
rs1690916	MDM2	primary	Per allele	Ewing's	2	Caucasian	0.62 [0.46, 0.83]	0	0.001	164	1522	C/T	AAA	N	INTERM
rs17206779	ADAMTS6	primary	Per allele	Osteo	2	Mixed	0.79 [0.67, 0.93]	35	0.004	1109	3507	C/T	ABA	N	INTERM
rs17655	ERCC5	primary	Recessive	Mixed	2	Caucasian	2.04 [1.07, 3.9]	0	0.03	223	515	G/C	BAA	N	INTERM
rs1799793	ERCC2	primary	Per allele	Osteo	2	Mixed	0.75 [0.58, 0.97]	23	0.03	271	532	G/A	BAA	N	INTERM
rs1799793	ERCC2	primary	Dominant	Osteo	2	Mixed	0.63 [0.44, 0.89]	0	0.009	271	532	G/A	BAA	N	INTERM
rs1800896	IL10	primary	Per allele	Osteo	2	Mixed	1.33 [1.06,1.66]	0	0.01	340	420	A/G	BAA	N	INTERM
rs1906953	GRM4	sensitivity	Per allele	Osteo	2	Asian	0.68 [0.55, 0.84]	0	0.0004	294	384	G/A	BAA	N	INTERM
rs2279744	MDM2	primary	Per allele	Mixed	4	Mixed	1.36 [1.06, 1.76]	26	0.02	448	563	T/G	ABA	N	INTERM
rs2279744	MDM2	primary	Recessive	Mixed	4	Mixed	1.58 [1.03, 2.42]	20	0.04	448	563	T/G	AAA	N	INTERM
rs2279744	MDM2	primary	Dominant	Mixed	4	Mixed	1.55 [1.05, 2.29]	36	0.03	448	563	T/G	ABA	N	INTERM
rs231775	CTLA4	primary	Recessive	Mixed	4	Asian	2 [1.53, 2.62]	0	<0.00001	1003	1162	G/A	AAA	N	INTERM
rs231775	CTLA4	primary	Dominant	Mixed	4	Asian	1.35 [1.14, 1.61]	0	0.0007	1003	1162	G/A	AAA	N	INTERM
rs231775	CTLA4	subgroup	Per allele	Ewing's	2	Asian	1.36 [1.15, 1.61]	0	0.0003	531	664	G/A	AAA	N	INTERM
rs231775	CTLA4	subgroup	Recessive	Ewing's	2	Asian	2 [1.39, 2.89]	0	0.0002	531	664	G/A	AAA	N	INTERM
rs231775	CTLA4	subgroup	Dominant	Ewing's	2	Asian	1.36 [1.07, 1.72]	0	0.01	531	664	G/A	AAA	N	INTERM
rs231775	CTLA4	subgroup	Per allele	Osteo	2	Asian	1.36 [1.13, 1.64]	0	0.001	472	498	G/A	ABA	N	INTERM
rs231775	CTLA4	subgroup	Recessive	Osteo	2	Asian	2 [1.34, 2.98]	0	0.0007	472	498	G/A	ABA	N	INTERM
rs231775	CTLA4	subgroup	Dominant	Osteo	2	Asian	1.35 [1.04, 1.75]	0	0.02	472	498	G/A	ABA	N	INTERM
rs3025039	VEGFA	primary	Per allele	Osteo	5	Asian	1.28 [1.12, 1.47]	0	0.0004	987	1344	C/T	AAA	N	INTERM
rs3025039	VEGFA	primary	Recessive	Osteo	5	Asian	1.65 [1.19, 2.27]	6	0.002	987	1344	C/T	AAA	N	INTERM
rs3025039	VEGFA	primary	Dominant	Osteo	5	Asian	1.24 [1.04, 1.47]	0	0.02	987	1344	C/T	AAA	N	INTERM
rs454006	PRKCG	primary	Recessive	Osteo	2	Asian	1.99 [1.54, 2.58]	0	<0.0001	998	998	T/C	AAA	N	INTERM
rs6599400	FGFR3	primary	Per allele	Osteo	2	Caucasian	1.53 [1.19, 1.97]	0	0.001	164	1522	C/A	AAA	N	INTERM
rs699947	VEGFA	primary	Per allele	Osteo	2	Asian	1.46 [1.19, 1.79]	0	0.0003	347	512	C/A	BAA	N	INTERM
rs699947	VEGFA	primary	Recessive	Osteo	2	Asian	1.73 [1.17, 2.55]	0	0.006	347	512	C/A	BAA	N	INTERM
rs699947	VEGFA	primary	Dominant	Osteo	2	Asian	1.51 [1.14, 2]	0	0.004	347	512	C/A	BAA	N	INTERM
rs820196	RECQL5	primary	Recessive	Osteo	2	Asian	2.15 [1.41, 3.29]	0	0.0004	397	441	T/C	BAA	N	INTERM
rs820196	RECQL5	primary	Dominant	Osteo	2	Asian	1.49 [1.12, 1.98]	0	0.006	397	441	T/C	BAA	N	INTERM
rs861539	XRCC3, KLC1	primary	Per allele	Osteo	2	Asian	1.57 [1.25, 1.97]	0	0.0001	288	440	C/T	BAA	N	INTERM
rs861539	XRCC3, KLC1	primary	Recessive	Osteo	2	Asian	2.23 [1.4, 3.57]	0	0.0008	288	440	C/T	BAA	N	INTERM
rs861539	XRCC3, KLC1	primary	Dominant	Osteo	2	Asian	1.57 [1.16, 2.13]	0	0.003	288	440	C/T	BAA	N	INTERM
deletion	GSTT1	primary	Recessive	Mixed	4	Mixed	1.32 [1.01, 1.73]	4	0.04	355	938	non-null/ null	AAA	N	INTERM
rs1042522	TP53	primary	Per allele	Mixed	6	Mixed	0.6 [0.39, 0.93]	84	0.02	788	950	G/C	ACA	N	LOW
rs1042522	TP53	subgroup	Per allele	Osteo	3	Mixed	0.47 [0.23, 0.95]	93	0.04	509	737	G/C	ACA	N	LOW
rs1129055	CD86	primary	Per allele	Mixed	2	Asian	0.33 [0.11, 1.01]	93	0.05	363	428	A/G	BCA	N	LOW
rs2305089	T	primary	Per allele	Chordoma	3	Mixed	2.87 [1.35, 6.08]	86	0.006	228	1001	G/A	ACA	N	LOW
rs2305089	T	primary	Recessive	Chordoma	2	Mixed	4.16 [1.21, 14.25]	82	0.02	125	841	G/A	BCA	N	LOW
rs6479860	LOC107984012 NRBF2	primary	Per allele	Ewing's	2	Caucasian	1.79 [1.36, 2.34]	66	<0.0001	744	4603	C/T	ACA	N	LOW
rs7591996	GRM4	primary	Per allele	Osteo	2	Mixed	1.28 [1.02, 1.61]	53	0.03	1109	3507	A/C	ACA	N	LOW
deletion	GSTM1	sensitivity	Recessive	Bone tumor	3	Asian	1.69 [1.02, 2.81]	66	0.04	315	578	non-null/ null	BCA	N	LOW

OR [95%CI]: Summary Odds Ratio [95% Confidence Interval]; Ref: reference allele; Alt: alternative allele; Venice criteria: A (high), B (moderate), C (weak) credibility for three parameters (amount of evidence, heterogeneity and bias); FPRP: false positive report probability at a prior probability of 10E-3; Y: noteworthy association (FPRP cut-off value 0.2), N: non noteworthy association; Level of evidence: overall level of summary evidence according to the Venice criteria and FPRP.

In order to provide an estimate of the impact of germline variants on sarcoma risk, the PAR (population attributable risk) was calculated. As an example, we considered the following three independent SNPs with high quality evidence on their relationship with sarcoma risk: rs11599754 of *ZNF365/EGR2* (chromosome 10, risk allele: C, risk allele frequency in European ancestry population: 0.39, meta-analysis OR: 1.48); rs231775 of *CTLA4* (chromosome 2, risk allele: A, risk allele frequency in European ancestry population: 0.65, meta-analysis OR: 1.36); and rs454006 of *PRKCG* (chromosome 19, risk allele: C, risk allele frequency in European ancestry population: 0.25, meta-analysis OR: 1.35). The PAR resulted equal to 37.2%.

### Associations based on single studies

Beside the variations resulted to be statistically significantly associated with sarcoma risk in this meta-analysis, we retrieved from the included articles 906 SNPs statistically significantly associated with sarcoma risk (P-value <0.05) based on single-study analysis. In Table 3 are reported 53 SNPs strongly associated with Ewing's sarcoma or osteosarcoma risk (P-value <E-06), retrieved from the included studies.

**Table 3:** Statistically significant associations based on single studies (P-value threshold E-06)

Reference	Cancer type	Genes	SNP ID	Ref/Alt	Chr	OR [95%CI]	P-value	location	eQTL	eQTL P-value skeletal muscle
Postel-Vinay S. [10]	Ewing's	C1orf127, TARDBP	rs9430161	T/G	1	2.20 [1.80, 2.70]	1.40E-20	intergene		
Postel-Vinay S. [10]	Ewing's	C1orf127	rs2003046	A/C	1	1.80 [1.50, 2.20]	1.30E-14	intron		
Postel-Vinay S. [10]	Ewing's	C1orf127	rs11576658	T/C	1	1.80 [1.40, 2.30]	9.40E-11	intron		
Postel-Vinay S. [10]	Ewing's	SRP14-AS1	rs4924410	C/A	15	1.50 [1.30, 1.70]	6.60E-09	intron	RP11-521C20.2	1.60E-07
Grünewald TG. [29]	Ewing's	ADO, EGR2	rs10995305	G/A	10	1.59 [1.26, 2.00]	4.38E-07	intergene	ADO	1.40E-16
Zhao J. [97]	Osteo	ARHGAP35	rs1052667	C/T	19	2.25 [1.64, 3.09]	4.43E-07	utr 3 prime	ARHGAP35	Other tissue
Grünewald TG. [29]	Ewing's	ADO, EGR2	rs224290	G/C	10	0.55 [0.43, 0.70]	7.80E-07	intergene	ADO	7.50E-14
Grünewald TG. [29]	Ewing's	ADO, EGR2	rs224291	G/A	10	0.55 [0.43, 0.70]	7.80E-07	intergene	ADO	7.20E-14
Grünewald TG. [29]	Ewing's	ADO, EGR2	rs224296	C/T	10	0.55 [0.43, 0.70]	7.80E-07	intergene	ADO	2.90E-14
Grünewald TG. [29]	Ewing's	ADO, EGR2	rs224297	T/C	10	0.55 [0.43, 0.70]	7.80E-07	intergene	ADO	2.80E-14
Grünewald TG. [29]	Ewing's	ADO, EGR2	rs224298	G/A	10	0.55 [0.43, 0.70]	7.80E-07	intergene	ADO	2.90E-14
Grünewald TG. [29]	Ewing's	ADO, EGR2	rs224294	C/T	10	0.54 [0.43, 0.69]	1.01E-06	intergene	ADO	5.60E-14
Grünewald TG. [29]	Ewing's	ADO, EGR2	rs224293	G/A	10	0.55 [0.44, 0.71]	1.02E-06	intergene	ADO	7.20E-14
Grünewald TG. [29]	Ewing's	EGR2, ADO	rs1848796	C/T	10	1.80 [1.42, 2.29]	1.08E-06	intergene	ADO	2.90E-14
Grünewald TG. [29]	Ewing's	ADO, EGR2	rs224282	A/G	10	0.55 [0.44, 0.71]	1.08E-06	intergene	ADO	7.20E-14
Savage SA. [11]	Osteo	ADAMTS17	rs2086452	T/C	15	1.35 [1.19, 1.52]	1.12E-06	intron		
Grünewald TG. [29]	Ewing's	EGR2	rs648746	G/T	10	0.56 [0.44, 0.71]	1.21E-06	upstream	ADO	5.10E-15
Grünewald TG. [29]	Ewing's	EGR2	rs648748	G/A	10	0.56 [0.44, 0.71]	1.21E-06	upstream	ADO	5.10E-15
Grünewald TG. [29]	Ewing's	EGR2	rs7076924	A/G	10	1.79 [1.41, 2.28]	1.21E-06	upstream	ADO	5.50E-15
Grünewald TG. [29]	Ewing's	EGR2	rs224277	T/C	10	0.56 [0.44, 0.71]	1.40E-06	upstream	ADO	3.30E-15
Grünewald TG. [29]	Ewing's	ADO, EGR2	rs224289	T/C	10	0.56 [0.44, 0.71]	1.42E-06	intergene	ADO	7.20E-14
Grünewald TG. [29]	Ewing's	ADO, EGR2	rs7096645	G/T	10	1.78 [1.40, 2.27]	1.54E-06	intergene	ADO	8.60E-14
Grünewald TG. [29]	Ewing's	LOC107984012, NRBF2	rs10740101	A/G	10	2.07 [1.55, 2.76]	2.29E-06	intergene	ADO	4.90E-10
Grünewald TG. [29]	Ewing's	EGR2, LOC107984012	rs7079482	C/T	10	2.06 [1.54, 2.76]	2.69E-06	intergene	ADO	1.70E-10
Grünewald TG. [29]	Ewing's	EGR2, LOC107984012	rs1115705	T/C	10	2.07 [1.55, 2.77]	2.73E-06	intergene	ADO	9.40E-11
Grünewald TG. [29]	Ewing's	EGR2, LOC107984012	rs983319	A/T	10	2.07 [1.55, 2.77]	2.99E-06	intergene	ADO	4.10E-10
Grünewald TG. [29]	Ewing's	EGR2, LOC107984012	rs1571918	A/G	10	2.05 [1.54, 2.74]	3.44E-06	intergene	ADO	2.80E-10
Grünewald TG. [29]	Ewing's	EGR2, LOC107984012	rs1888968	C/T	10	2.05 [1.54, 2.74]	3.44E-06	intergene	ADO	1.90E-10
Grünewald TG. [29]	Ewing's	EGR2, LOC107984012	rs1912369	G/A	10	2.05 [1.54, 2.74]	3.44E-06	intergene	ADO	3.50E-10
Grünewald TG. [29]	Ewing's	EGR2, LOC107984012	rs4147153	A/G	10	2.05 [1.54, 2.74]	3.44E-06	intergene	ADO	3.50E-10
Grünewald TG. [29]	Ewing's	EGR2, LOC107984012	rs4237316	C/T	10	2.05 [1.54, 2.74]	3.44E-06	intergene	ADO	1.90E-10
Grünewald TG. [29]	Ewing's	EGR2, LOC107984012	rs4746746	C/T	10	2.05 [1.54, 2.74]	3.44E-06	intergene	ADO	7.20E-10
Grünewald TG. [29]	Ewing's	EGR2, LOC107984012	rs6479854	C/T	10	2.05 [1.54, 2.74]	3.44E-06	intergene	ADO	1.50E-10
Grünewald TG. [29]	Ewing's	EGR2, LOC107984012	rs7100213	T/C	10	2.05 [1.54, 2.74]	3.44E-06	intergene	ADO	2.10E-10
Grünewald TG. [29]	Ewing's	EGR2, LOC107984012	rs4746745	T/C	10	2.03 [1.52, 2.72]	3.48E-06	intergene	ADO	5.90E-11
Grünewald TG. [29]	Ewing's	ADO, EGR2	rs224301	G/A	10	0.60 [0.47, 0.76]	3.67E-06	intergene	ADO	1.20E-10
Grünewald TG. [29]	Ewing's	ADO, EGR2	rs224302	G/A	10	0.60 [0.47, 0.76]	3.67E-06	intergene	ADO	3.70E-10
Grünewald TG. [29]	Ewing's	ADO, EGR2	rs10822056	C/T	10	1.65 [1.31, 2.09]	3.70E-06	intergene	ADO	3.00E-13
Grünewald TG. [29]	Ewing's	ADO, EGR2	rs224295	A/C	10	0.60 [0.48, 0.76]	4.80E-06	intergene	ADO	1.50E-10
Grünewald TG. [29]	Ewing's	ADO, EGR2	rs224299	T/C	10	0.60 [0.48, 0.76]	4.80E-06	intergene	ADO	1.50E-10
Savage SA. [11]	Osteo	LOC105373401, LOC105373402	rs13403411	C/T	2	1.30 [1.16, 1.46]	5.20E-06	intergene		
Grünewald TG. [29]	Ewing's	EGR2, LOC107984012	rs1509952	C/T	10	2.06 [1.54, 2.76]	5.28E-06	intergene	ADO	3.50E-10
Grünewald TG. [29]	Ewing's	LOC107984012	rs10740095	T/C	10	2.03 [1.52, 2.72]	5.50E-06	intron	ADO	4.20E-11
Grünewald TG. [29]	Ewing's	LOC107984012	rs925307	T/C	10	2.03 [1.52, 2.72]	5.50E-06	intron	ADO	6.00E-11
Grünewald TG. [29]	Ewing's	EGR2, LOC107984012	rs7073383	A/G	10	2.01 [1.50, 2.69]	5.98E-06	intergene	ADO	1.60E-10
Grünewald TG. [29]	Ewing's	EGR2, LOC107984012	rs10733780	G/T	10	2.01 [1.50, 2.69]	6.90E-06	intergene	ADO	2.90E-10
Grünewald TG. [29]	Ewing's	LOC107984012	rs7071512	T/C	10	2.01 [1.50, 2.69]	6.90E-06	intron	ADO	4.20E-11
Savage SA. [11]	Osteo	FAM208B, GDI2	rs2797501	A/G	10	0.62 [0.51, 0.77]	7.88E-06	missense, downstream		
Savage SA. [11]	Osteo	DLEU1, LOC107984568	rs573666	G/A	13	0.77 [0.68, 0.86]	8.59E-06	intergene	EBPL	Other tissue
Grünewald TG. [29]	Ewing's	EGR2, LOC107984012	rs10740097	C/T	10	2.03 [1.51, 2.72]	9.03E-06	intergene	ADO	1.20E-10
Grünewald TG. [29]	Ewing's	LOC107984012	rs6479848	T/C	10	2.01 [1.50, 2.69]	9.16E-06	intron	ADO	2.70E-11
Grünewald TG. [29]	Ewing's	ZNF365, ADO, EGR2	rs224079	C/T	10	1.58 [1.25, 2.01]	9.24E-06	intergene	ADO	5.00E-22
Grünewald TG. [29]	Ewing's	LOC107984012	rs965128	C/T	10	1.99 [1.49, 2.66]	9.48E-06	intron	ADO	3.10E-11

OR [95%CI]: Odds Ratio [95% Confidence Interval]; Ref: reference allele; Alt: alternative allele; eQTL: expression quantitative trait locus.

One dataset was available for each of those genetic variants. Although it was not possible to perform a meta-analysis, a strong association with sarcoma risk was found (P-values range from E-20 to E-06). Ewing's sarcoma associations in European and US European-descendant population mainly involved the candidate risk loci at 1p36.22, 10q21 reported by Postel-Vinay et al [10] GWAS and in the following related study of Grünewald et al [29]. The 1p36.22 variants associated with Ewing's sarcoma are located 25 kb proximal to the *TARDBP* gene. TARDBP (Tat activating regulatory DNA-binding protein, or TDP-43, transactive response DNA-binding protein) is a highly conserved DNA- and RNA-binding protein involved in RNA transcription and splicing. The 10q21 variants strongly associated with Ewing's sarcoma are located in a block containing four genes: *ADO* (encoding cysteamine dioxygenase), *ZNF365* (encoding zinc-finger protein 365), *EGR2* (encoding early growth response protein 2) and LOC107984012 (unknown function).

A further association with osteosarcoma in Guangxi population was studied by Zhao et al [97] regarding the Rho GTPase-activating protein 35 (ARHGAP35), a Rho family GTPase-activating protein. Finally Savage et al [11] GWAS found associations with osteosarcoma and GMR4 (glutamate receptor metabotropic 4), which were part of our meta-analysis and ADAMTS protein family, as ADAM Metallopeptidase with Thrombospondin Type 1 Motif 17. Of note, most statistically significant associations based on single studies did not have a statistically significant eQTL effect.

### Network and pathway analysis findings

**Figure 2:**
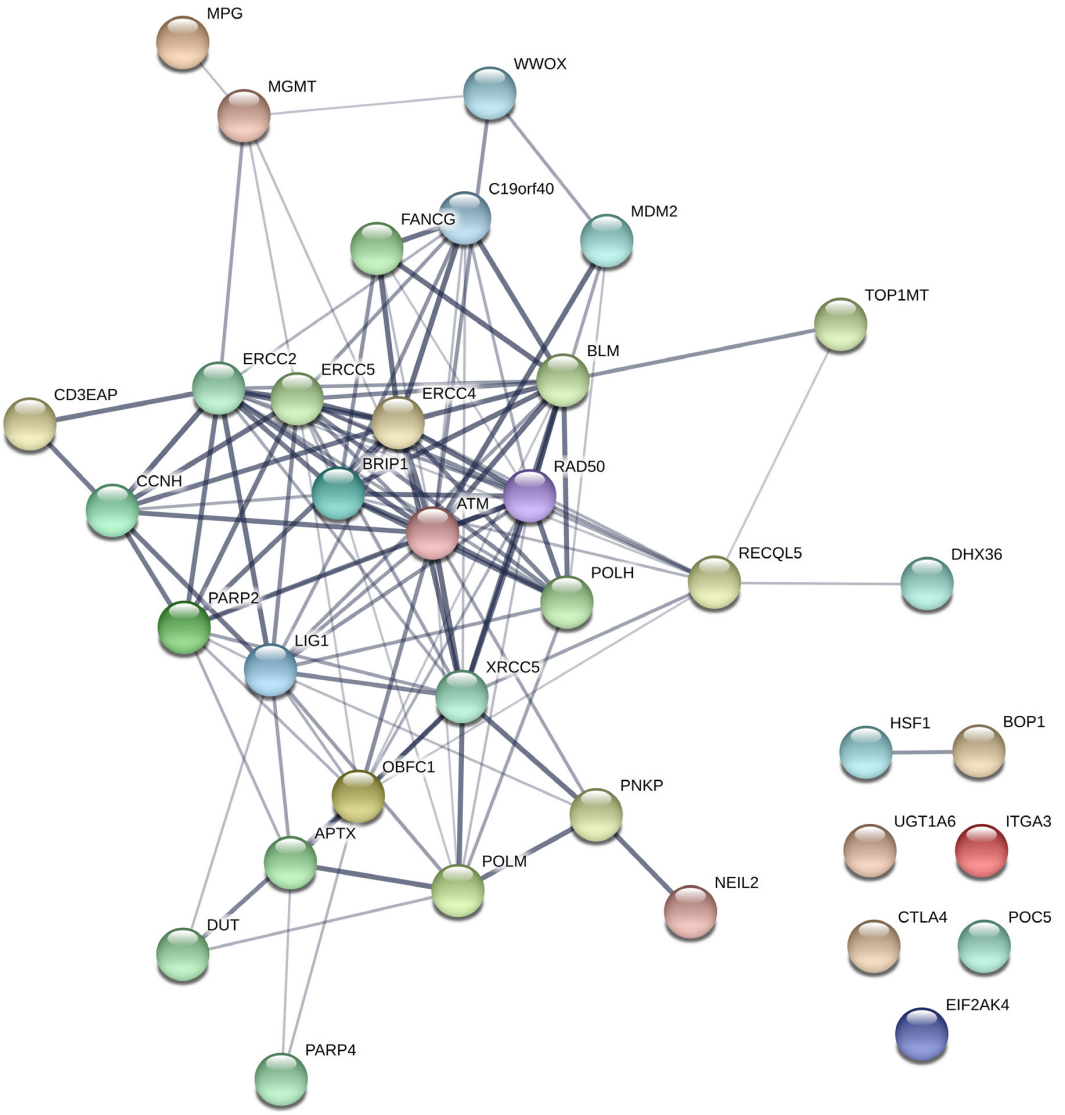
Network analysis of proteins encoded by genes whose variants associated with sarcoma risk and characterized by an expression quantitative trait locus effect (eQTL) The figure illustrates the high degree of connectivity of these proteins, which result to be enriched in DNA repair pathway components.

Using the 36 genes whose SNPs were significantly associated with sarcoma risk (including data from both meta-analysis and single studies) and were also characterized by a significant eQTL effect, we found that the corresponding protein products interact with each other beyond chance (observed edges: 120; expected edges: 12; PPI enrichment P-value <10E-20), with an average node degree equal to 6.7 (see Figure 2). Such enrichment indicates that the input molecules - as a whole group - are at least partially biologically connected. This high connectivity prompted us to conduct pathway analysis, which showed that the identified network is significantly enriched in DNA repair proteins, as shown in Table 4.

**Table 4:** Pathway analysis main findings: gene set enrichment analysis based on 36 sarcoma risk genes. Enrichments with at least ten overlapping genes are shown

Pathway	Overlap	FDR	Genes	Database
Base excision repair (BER)	11/139	0.002374441	BLM;RAD50; PARP4; RECQL5; LIG1; MPG; PARP2; ERCC4; PNKP; FANCG; POLH	GO biol process
DNA 3' dephosphorylation involved in DNA repair	10/120	0.002376199	BLM; RAD50; PARP4; RECQL5; LIG1; PARP2; ERCC4; PNKP; FANCG; POLH	GO biol process
DNA dealkylation involved in DNA repair	12/128	0.000983329	BLM; RAD50; PARP4; RECQL5; LIG1; MPG; MGMT; PARP2; ERCC4; PNKP; FANCG; POLH	GO biol process
DNA ligation involved in DNA repair	11/132	0.002374441	BLM; RAD50; PARP4; RECQL5; LIG1; MGMT; PARP2; ERCC4; PNKP; FANCG; POLH	GO biol process
DNA repair	18/285	8.22494E-05	BLM; LIG1; CCNH; XRCC5; PARP2; MGMT; MPG; POLM; PNKP; FANCG; BRIP1; RAD50; NEIL2; ERCC4; ERCC2; ATM; ERCC5; POLH	Reactome
DNA synthesis involved in DNA repair	12/142	0.001514863	BLM; BRIP1; RAD50; PARP4; RECQL5; LIG1; PARP2; ERCC4; PNKP; ATM; FANCG; POLH	GO biol process
Double-strand break repair (DSBR)	12/164	0.002374441	BLM; BRIP1; RAD50; PARP4; RECQL5; LIG1; XRCC5; PARP2; ERCC4; PNKP; FANCG; POLH	GO biol process
Mismatch repair (MMR)	10/140	0.005835867	BLM; RAD50; PARP4; RECQL5; LIG1; PARP2; ERCC4; PNKP; FANCG; POLH	GO biol process
Mitochondrial DNA repair	10/123	0.002552586	BLM; RAD50; PARP4; RECQL5; LIG1; PARP2; ERCC4; PNKP; FANCG; POLH	GO biol process
Non homologous end joining (NHEJ)	10/120	0.002376199	BLM; RAD50; PARP4; RECQL5; LIG1; PARP2; ERCC4; PNKP; FANCG; POLH	GO biol process
Nucleotide excision repair (NER)	11/138	0.002374441	BLM; RAD50; PARP4; RECQL5; LIG1; PARP2; ERCC4; PNKP; ERCC2; FANCG; POLH	GO biol process
Nucleotide phosphorylation involved in DNA repair	10/120	0.002376199	BLM; RAD50; PARP4; RECQL5; LIG1; PARP2; ERCC4; PNKP; FANCG; POLH	GO biol process
Homologous recombination (HR)	10/132	0.00369711	BLM; RAD50; PARP4; RECQL5; LIG1; PARP2; ERCC4; PNKP; FANCG; POLH	GO biol process
Single strand break repair (SSBR)	11/124	0.001805921	BLM; RAD50; PARP4; RECQL5; LIG1; PARP2; ERCC4; PNKP; APTX; FANCG; POLH	GO biol process
UV-damage excision repair	11/158	0.003533915	BLM; RAD50; PARP4; RECQL5; LIG1; PARP2; ERCC4; PNKP; EIF2AK4; FANCG; POLH	GO biol process
XPC complex (NER)	15/160	8.19637E-06	WWOX; CCNH; XRCC5; MGMT; CD3EAP; FANCG; POC5; ERCC4; ERCC2; MDM2; OBFC1; ATM; ERCC5; POLH; UGT1A6	Jenesen compartments

FDR: false discovery rate.

In particular, many sarcoma risk genes appear to be involved in all main DNA repair pathways, including single strand break repair pathways (base excision repair [BER], nucleotide excision repair [NER], mismatch repair [MMR]) and double strand repair pathways (non homologous end joining [NHEJ], homologous recombination [HR]).

## DISCUSSION

We described the findings of the first field synopsis and meta-analysis dedicated to the relationship between germline DNA variation and risk of developing bone and soft tissue sarcomas, which is based on genotyping data from 90 studies enrolling almost 48,000 people with a control-to-case ratio equal to 2. The resulting knowledgebase will be hosted by our cancer-dedicated website (at www.mmmp.org) [100] as a freely available online data repository that will be annually updated.

Overall, our findings support the hypothesis that genetic polymorphism does contribute to sarcoma susceptibility. This is exemplified by the population attributable risk (PAR=37.2%) calculated for three SNPs associated with the risk of sarcoma at a high level of evidence (rs11599754 of *ZNF365/EGR2*, rs231775 of *CTLA4*, and rs454006 of *PRKCG*), which indicates that more than one third of sarcoma cases would not occur in a hypothetical population where these three risk variants were absent. This remarkable influence of just three SNPs is linked not only to the high frequency of the risk alleles but also to the interesting fact that the risk, defined as odds ratio, associated with single variants ranged between 1.35 and 1.48, which are values higher than those usually observed for other malignancies such as breast [101], colorectal [102], and gastric carcinomas [103], which generally include odds ratios between 1.10 and 1.30. Considering that the mean risk among variants significantly associated with sarcoma predisposition was even higher (approximately 1.70, see Table 2), one might speculate that germline DNA variation is especially important in the determinism of the susceptibility to this family of tumors.

Overall, the quality of the available data, which was thoroughly assessed by means of both Venice criteria and false positive report probability (FPRP), was satisfactory considering that the statistically significant evidence on 47 of 55 variants for which a meta-analysis was feasible was classified as high to moderate level of quality with 10 SNPs considered adequate according to the FPRP (Table 2). A statistically significant association was also demonstrated for additional 906 SNPs, for which only a single data source was available, which pinpoints the urgent need for replication studies in order to validate or refute these findings.

Conventional meta-analysis of single variants led us to identify 55 SNPs significantly associated with sarcoma risk (Table 2), and additional 53 SNPs were reported in single studies (Table 3): these variants are linked to a variety of genes whose protein products are involved in several cell activities. Therefore, we tried to provide readers with a preliminary interpretation of these findings from the functional biology viewpoint. Using modern SNP-to-gene and gene-to-function approaches such as integrative analysis of genetic variation with expression quantitative trait locus (eQTL) data [9] and respectively pathway/network analysis [8], we hypothesize that germline variation of the DNA repair machinery might be of special relevance for the development of this type of cancer (Figure 2). This finding – which has been very recently confirmed in patients with Ewing's sarcoma [104] - is in line with the complex gene and chromosome abnormalities that characterized some sarcoma histologies, as well as with the epidemiological observation that people accidentally [105] or therapeutically [106] exposed to ionizing radiations and thus prone to develop DNA damage are at higher risk of different types of sarcomas. In this regard, it is interesting to note that peripheral blood mononuclear cells of patients diagnosed with sarcomas show a higher sensitivity to mutagens *in vitro* as compared to controls [107], which supports the hypothesis that the genetic background can make the difference on an individual basis in terms of response to environmental carcinogens potentially involved in sarcomagenesis.

Finally, also somatic DNA alterations appear to confer a defective DNA repair capability to some sarcoma types such as Ewing's sarcoma [108], and thus the combinatory study of germline and somatic DNA variations characterizing sarcomas might lead to better understand the cascade of molecular events underlying sarcomagenesis, as recently proposed for the *EWSR1-FLI1* fusion gene and the SNPs near *EGR2* in Ewing's sarcoma patients [29].

Overall, these converging data suggest that more investigation aimed to fully elucidate whether the germline individual capacity of repairing genomic damage can actually affect the predisposition to a complex and heterogeneous trait such as sarcomas might be particularly fruitful.

In our work we also confirmed the association between sarcoma risk and variants of single genes, such as *ZNF365, ADO, EGR2, CTLA4, TP53, CD86, NUDT6, MDM2, ERCC5* and *ADAMTS6* just to mention the top ten by statistical significance. Many of these genes are not known to be involved in DNA repair and thus the relationship between these single gene findings and network/pathway analysis might appear of unclear interpretation and doubtful importance. However, we must remember that current evidence (and thus our analysis) is based on 88 candidate gene studies and only two GWAS: therefore, more extensive investigation is needed on the variation of pathways for which data on single genes are currently available. In this regard, our meta-analysis data can be utilized to inform future studies on candidate pathways whose genetic variation could affect sarcoma susceptibility.

This systematic review also underscores the main limitation of the evidence on the genetic susceptibility of sarcomas. In fact, most of current information is driven by data from studies investigating bone tumors (78 of 90, 86.6%). Studies focusing on soft tissue sarcomas are thus eagerly awaited, the formation of international consortia being advocated in order to overcome the hurdle of disease rarity. Hopefully, technological improvements in direct DNA sequencing such as next generation sequencing (NGS) methods will further accelerate the discovery pace in this field of investigation, as recently reported [104].

Nevertheless, we also recognize some limitations of this synopsis: data from different tumor types and population ethnicity were pooled together to find associations despite the diversity of sarcoma histologies, leading to high level of between-study heterogeneity. To overcome to this limitation we performed subgroup and sensitivity analysis whenever possible. Moreover, despite our efforts to avoid the issue of overlapping series, it is always possible that partial overlaps between multiple series published by the same research groups that cannot be detected by full text reading did remain included in pooled analyses: however, we believe that the influence of this potential residual overlapping on the overall results is reasonably low.

In conclusion, we hope that the creation of the first knowledgebase dedicated to the relationship between germline DNA variation and sarcoma risk can not only represent a valuable reference for investigators involved in sarcoma research but also inform future studies based on the gaps of the current literature.

## MATERIALS AND METHODS

### Search strategy, eligibility criteria, quality score assessment and data extraction

This study followed the principles proposed by the Human Genome Epidemiology Network (HuGeNet) for the systematic review of molecular association studies [109].

We considered eligible all the studies concerning the association between any genetic variant and the predisposition to sarcoma in humans, providing the raw data necessary to calculate risk of developing a sarcoma or the summary data. Exclusion criteria were: virus-induced sarcomas (HHV8 - Kaposi sarcoma); sarcomas secondary to radiation therapy; sarcomas secondary to burns/scars/surgery; associations between mitochondrial DNA variations and sarcomas; gastrointestinal stromal tumors (GIST).

Database search of original articles analyzing the association between any genetic variant and susceptibility to sarcoma was conducted independently by two investigators though the following database: MEDLINE (via the PubMed gateway); The Cochrane Library; Scopus; Web of Science. The search included the following three groups of keywords: 1) sarcoma, solitary fibrous tumor, chordoma, tenosynovitis, fibromatosis, desmoids, myofibroblastic, myopericytoma, myxoma, Ewing, desmoplastic, PEComa, haemangioendothelioma, lymphangioma, myoepithelioma; 2) risk, sarcomagenesis, tumorigenesis, predisposition, susceptibility; 3) polymorphism, SNP, variant, genome wide association study and its acronym GWAS. Searches were conducted using all combinations of at least one keyword from each group. References from eligible articles were also used to refine the literature search.

The quality of the studies was evaluated according to Newcastle-Ottawa quality assessment scale (NOS) [110]. In brief, the following three parameters were evaluated with a “star system”: the selection of the study groups (0 to 4 “stars”), the comparability of the groups (0 to 2 “stars”), and the ascertainment of either the exposure or outcome of interest for case-control or cohort studies respectively (0 to 3 “stars”). The maximum total score was 9 “stars” and represented the highest quality.

Data were extracted independently by two investigators using a template. Every disagreement was resolved by a third investigator in order to reach consensus. Authors were contacted whenever unreported data were potentially useful to enable the inclusion of the study into the systematic review. The data extracted from eligible studies were: authors, journal, year of publication, region or country where the study was conducted, hospital where the patients were diagnosed, number of patients with sarcoma enrolled and healthy control subjects, period of enrolment, prevalent ethnicity (>80%, categorized in Caucasian, Asian, African and mixed), subjects age, genetic polymorphisms and allelic frequency in both cases and controls (if no raw data were available, summary data were collected, i.e. odds ratios and confidence intervals), study design (population-based versus hospital-based), statistical methods used, and sarcoma histology.

We considered data published in different articles by the same Author/s with the same (or similar) number of subjects enrolled in the same period of time in the same hospital, to be derived by the same group of patients. In publications with either overlapping cases or controls, the most recent or largest population was chosen.

For analysis purposes, the search was closed in August 2017.

### Statistical analysis

We calculated summary odds ratios (ORs) and their corresponding 95% confidence intervals (95%CI) starting from raw data to measure the strength of association between each polymorphism and sarcoma risk.

Whenever possible, we calculated the pooled ORs assuming 3 different genetic models: per-allele (additive), dominant and recessive. If the included studies reported exclusively per-allele ORs, as in GWAS, we calculated the pooled OR assuming the per-allele (additive) model.

Random effects meta-analysis based on the inverse variance method was used to calculate summary ORs; this model reduces to a fixed effect meta-analysis if between-study heterogeneity is absent. We chose this model for the large between-study heterogeneity usually expected in genetic association studies. A meta-analysis was performed only if at least two independent data sources were available. In case of GWAS, we considered as data source the joint analysis between the discovery and the validation phases. Subgroup analysis by histological subtype (Ewing's sarcoma vs osteosarcoma) was planned if data permitted.

Regarding ethnicity, analyses were divided in 4 groups: African (if the datasets were all African population-based), Asian (if the datasets were all Asian population-based), Caucasian (if the datasets were all Caucasian population-based), and mixed (if the datasets were African, Asian and Caucasian or if the datasets were from mixed ethnicity). In order to test any dominant study driving effect, sensitivity analysis by ethnicity (Asian vs Caucasian/other) was performed in mixed meta-analyses, with more than two datasets, excluding either the Asian study or the Caucasian study from the meta-analysis.

Between-study heterogeneity was formally assessed by the Cochran Q-test and the I-squared statistic, the latter indicating the proportion of the variability in effect estimates linked to true between-study heterogeneity as opposed to within-study sampling error.

All statistical analyses were performed with RevMan 5 (Review Manager computer program, version 5.3; Copenhagen, The Nordic Cochrane Centre, The Cochrane Collaboration, 2014).

### Assessment of cumulative evidence

With the aim to assess the credibility of statistically significant associations based on the results of data meta-analysis, we used the Venice criteria [111]. In brief, we defined credibility levels based on the strength (classified as A=strong, B=moderate or C=weak) of three following parameters: amount of the evidence, replication of the association and protection from bias. We graded the amount of evidence, which approximately depends on the study sample size, based on the sum of cases and controls. Grade A, B or C was assigned to meta-analyses with total sample size >1000, 100–1000 and <100, respectively. Also, the replication of the association was graded considering the amount of between-study heterogeneity. We assigned grade A, B or C to meta-analyses with I-squared <25%, 25–50% and >50%, respectively. We graded protection from bias as A if no bias was observed, B if bias was potentially present or C if bias was evident. While assessing protection from bias we also considered the magnitude of the association. We assigned a score of C to an association characterized by a summary OR<1.15 or a summary OR>0.87 if the effect of the polymorphism was protective.

In addition to the Venice criteria, we assessed the noteworthiness of significant findings by calculating the false positive report probability (FPRP) [112], which is defined as the probability of no true association between a genetic variant and disease (null hypothesis) given a statistically significant finding. FPRP is based not only on the observed P-value of the association test but also on the statistical power of the test and on the prior probability that the molecular association is real following a Bayesian approach. We calculated FPRP values for two levels of prior probabilities: at a low prior (10E-3) that would be similar to what is expected for a candidate variant, and at a very low prior (10E-6) that would be similar to what would be expected for a random variant. To classify a significant association as ‘noteworthy’, we used a FPRP cut-off value of 0.2.

Overall, we defined the credibility level of the cumulative evidence as high (Venice criteria A grades only coupled with “noteworthy” finding at FPRP analysis), low (one or more C grades combined with lack of noteworthiness), or intermediate (for all other combinations).

To estimate the impact of genetic variation on the risk of sarcomas, we calculated the so called population attributable risk (PAR) using the following formula:

Pr (RR − 1)/[1 + Pr (RR − 1)],

where Pr is the proportion of control subjects exposed to the allele of interest and the relative risk (RR) was estimated using the summary estimates (i.e. ORs) calculated by the meta-analysis. The joint PAR for combinations of polymorphisms was calculated as follows:

1 − (∏_1→n_[1 − PARi]),

where PARi corresponds to the individual PAR of the *i*th polymorphism and *n* is the number of polymorphisms considered [113].

### Network and pathway analysis

In order to explore the mechanisms underlying the pathogenesis of sarcomas, we utilized network and pathway analysis to test the hypothesis that genes whose variations are associated with sarcoma risk interact with each other possibly within the frame of some specific molecular pathways [8].

To this aim, we first selected SNPs significantly associated with sarcoma risk. In case of SNPs located in intergenic regions we selected the first closest and the second closest genes, not necessarily upstream and downstream of the SNPs of interest.

Since most SNPs are intergenic or intronic and thus no obvious functional effect can be inferred, expression quantitative trait locus (eQTL) analysis was used to identify genes whose expression is affected by DNA variants [114]. The resulting gene list was the input for both network and pathway analysis.

For the former, the STRING web server was employed to study protein-protein interaction (PPI) across the selected genes [115], the confidence score being set >0.4. As a measure of across network connectivity STRING provides the average node degree, where degree is the conceptually simplest centrality measure as it measures the number of edges between protein connections attached to a protein; moreover, STRING computes the PPI enrichment P-value, which is significant when input proteins have more interactions among themselves than what would be expected for a random set of proteins of similar size, drawn from the genome.

As regards pathway analysis, the Enrichr web server was utilized to identify in our list over-representation of genes involved in specific pathways described in dedicated databases [116]. Hypergeometric distribution with Fisher's exact test was used to calculate the statistical significance of gene overlapping, followed by correction for multiple hypotheses testing using the false discovery rate [FDR] method.

### Declarations

Ethics approval and consent to participate: Not applicable

Consent for publication: Not applicable

Availability of data and material: All data generated or analysed during this study are included in this published article [and its supplementary information files].

## SUPPLEMENTARY MATERIALS AND TABLES








