# Supplementary material for: Genetic susceptibility to bone and soft tissue sarcomas: a field synopsis and meta-analysis
Source: Oncotarget. 2018 Apr 6;9(26):18607–26. doi: 10.18632/oncotarget.24719 (PMC5915097; doi:10.18632/oncotarget.24719)
Supplement: Supplementary file 1 [file oncotarget-09-18607-s001.pdf]

Genetic susceptibility to bone and soft tissue sarcomas: a field synopsis and meta-analysis

SUPPLEMENTARY MATERIALS

Supplementary Table 1: Polymorphisms characteristics. In blue: variation analyzed by different Authors in different datasets

See Supplementary File 1

Supplementary Table 2: Meta-analysis results

See Supplementary File 2

Supplementary Table 3: Polymorphisms rs IDs for variations defined in the original papers (abstract or text) with the only nucleotide change or Aminoacid change

See Supplementary File 3
