# Supplementary material for: Genetic susceptibility to bone and soft tissue sarcomas: a field synopsis and meta-analysis
Source: Oncotarget. 2018 Apr 6;9(26):18607–26. doi: 10.18632/oncotarget.24719 (PMC5915097; doi:10.18632/oncotarget.24719)
Supplement: Supplementary file 2 [file oncotarget-09-18607-s002.docx]

| **First Author and year** | **Journal** | **Gene** | **Chrom** | **Polymorphism ID** | **P-value** | **Polymorphism position** |
| --- | --- | --- | --- | --- | --- | --- |
| Mirabello L., 2011 | BMC Cancer | COMMD4 | 15 | rs11072542 | 0,0437 | downstream variant 500B |
| Mirabello L., 2011 | BMC Cancer | CD79B | 17 | rs11079515 | 0,0016 | downstream variant 500B |
| Savage SA., 2013 | Nat Genet. | EIF3H | 8 | rs1347626 | 0,0112 | downstream variant 500B |
| Savage SA., 2007 | Pediat Blood Cancer | TP53 | 17 | rs1614984 | 0,0142 | downstream variant 500B |
| Savage SA., 2013 | Nat Genet. | MPG, NPRL3 | 16 | rs2541622 | 0,0494 | downstream variant 500B |
| Feng D., 2013 | Genet Test Mol Biomarkers | CTLA4 | 2 | rs3087243 | 0,7488 | downstream variant 500B |
| Liu Y., 2011 | DNA Cell Biol. | CTLA4 | 2 | rs3087243 | 0,6597 | downstream variant 500B |
| Mirabello L., 2011 | BMC Cancer | PARP2 | 14 | rs3093942 | 0,0452 | downstream variant 500B |
| Savage SA., 2013 | Nat Genet. | EEF1D, NAPRT | 8 | rs4874156 | 0,0417 | downstream variant 500B |
| Walsh KM., 2016 | Carcinogenesis. | NAF1 | 4 | rs7675998 | 0,3443 | downstream variant 500B |
| Wu Y., 2015 | Tumor Biol. | B9D2, TGFB1 | 19 | rs1800469 | 0,7681 | downstream variant 500B,upstream variant 2KB |
| Xu S., 2014 | DNA Cell Biol. | B9D2, TGFB1 | 19 | rs1800469 | 0,6142 | downstream variant 500B,upstream variant 2KB |
| Savage SA., 2013 | Nat Genet. | LOC107986976 | 8 | rs10092594 | 0,0162 | intergene |
| Savage SA., 2013 | Nat Genet. | LOC105375760 | 8 | rs10103835 | 0,0477 | intergene |
| Savage SA., 2013 | Nat Genet. | SNTB1 | 8 | rs10110670 | 0,0366 | intergene |
| Savage SA., 2013 | Nat Genet. | LOC101927845 | 8 | rs10111298 | 0,0184 | intergene |
| Mirabello L., 2011 | BMC Cancer | PARP2 | 14 | rs10147163 | 0,0331 | intergene |
| Jiang C., 2014 | Med Oncol. | LINC01247, LOC105373402 | 2 | rs10208273 | 0,0375 | intergene |
| Savage SA., 2013 | Nat Genet. | LINC01247, LOC105373402 | 2 | rs10208273 | 2,93E-07 | intergene |
| Grünewald TG., 2015 | Nat Genet. | EGR2 | 10 | rs1040875 | 1,5060E-04 | intergene |
| Savage SA., 2013 | Nat Genet. | KCNK9 | 8 | rs10435688 | 0,044 | intergene |
| Savage SA., 2013 | Nat Genet. | KHDRBS3, LOC107986978 | 8 | rs10464971 | 0,0086 | intergene |
| Grünewald TG., 2015 | Nat Genet. | EGR2, ADO, LOC107984012 | 10 | rs10509173 | 9,9440E-04 | intergene |
| Grünewald TG., 2015 | Nat Genet. | EGR2, LOC107984012 | 10 | rs10733780 | 6,8970E-06 | intergene |
| Grünewald TG., 2015 | Nat Genet. | EGR2 | 10 | rs10740087 | 1,3170E-03 | intergene |
| Grünewald TG., 2015 | Nat Genet. | EGR2 | 10 | rs10740088 | 7,9820E-04 | intergene |
| Grünewald TG., 2015 | Nat Genet. | EGR2 | 10 | rs10740089 | 7,9820E-04 | intergene |
| Grünewald TG., 2015 | Nat Genet. | EGR2 | 10 | rs10740090 | 6,5310E-04 | intergene |
| Grünewald TG., 2015 | Nat Genet. | EGR2, ADO, LOC107984012 | 10 | rs10740091 | 9,9440E-04 | intergene |
| Grünewald TG., 2015 | Nat Genet. | LOC107984012 | 10 | rs10740092 | 1,8370E-04 | intergene |
| Grünewald TG., 2015 | Nat Genet. | EGR2, LOC107984012 | 10 | rs10740097 | 9,0330E-06 | intergene |
| Grünewald TG., 2015 | Nat Genet. | LOC107984012 | 10 | rs10740098 | 6,2310E-03 | intergene |
| Grünewald TG., 2015 | Nat Genet. | LOC107984012 | 10 | rs10740099 | 6,4020E-03 | intergene |
| Grünewald TG., 2015 | Nat Genet. | LOC107984012 | 10 | rs10740100 | 7,5470E-03 | intergene |
| Grünewald TG., 2015 | Nat Genet. | LOC107984012, NRBF2 | 10 | rs10740101 | 2,2850E-06 | intergene |
| Grünewald TG., 2015 | Nat Genet. | ADO, EGR2 | 10 | rs10761660 | 6,2970E-05 | intergene |
| Postel-Vinay S., 2012 | Nat Genet. | ADO, EGR2 | 10 | rs10761660 | 1,70E-10 | intergene |
| Grünewald TG., 2015 | Nat Genet. | ZNF365, ADO, EGR2 | 10 | rs10761661 | 2,1800E-05 | intergene |
| Grünewald TG., 2015 | Nat Genet. | EGR2, ADO, LOC107984012 | 10 | rs10761663 | 1,2700E-03 | intergene |
| Grünewald TG., 2015 | Nat Genet. | EGR2, ADO, LOC107984012 | 10 | rs10761664 | 2,9720E-03 | intergene |
| Grünewald TG., 2015 | Nat Genet. | EGR2, ADO, LOC107984012 | 10 | rs10761665 | 1,1800E-03 | intergene |
| Grünewald TG., 2015 | Nat Genet. | EGR2, ADO, LOC107984012 | 10 | rs10761666 | 7,1650E-05 | intergene |
| Grünewald TG., 2015 | Nat Genet. | EGR2, ADO, LOC107984012 | 10 | rs10761667 | 7,0410E-05 | intergene |
| Grünewald TG., 2015 | Nat Genet. | EGR2, ADO, LOC107984012 | 10 | rs10761668 | 9,9440E-04 | intergene |
| Grünewald TG., 2015 | Nat Genet. | EGR2, ADO, LOC107984012 | 10 | rs10761669 | 9,9440E-04 | intergene |
| Grünewald TG., 2015 | Nat Genet. | EGR2, ADO, LOC107984012 | 10 | rs10761670 | 9,9440E-04 | intergene |
| Grünewald TG., 2015 | Nat Genet. | EGR2, ADO, LOC107984012 | 10 | rs10761671 | 9,9440E-04 | intergene |
| Grünewald TG., 2015 | Nat Genet. | EGR2, ADO, LOC107984012 | 10 | rs10761673 | 9,5120E-04 | intergene |
| Grünewald TG., 2015 | Nat Genet. | EGR2, ADO, LOC107984012 | 10 | rs10761674 | 9,9440E-04 | intergene |
| Grünewald TG., 2015 | Nat Genet. | EGR2, ADO, LOC107984012 | 10 | rs10761675 | 2,3370E-03 | intergene |
| Grünewald TG., 2015 | Nat Genet. | EGR2, ADO, LOC107984012 | 10 | rs10761676 | 1,6700E-03 | intergene |
| Grünewald TG., 2015 | Nat Genet. | ZNF365 | 10 | rs10822052 | 3,9790E-05 | intergene |
| Grünewald TG., 2015 | Nat Genet. | ZNF365 | 10 | rs10822053 | 3,6190E-05 | intergene |
| Grünewald TG., 2015 | Nat Genet. | ZNF365, ADO, EGR2 | 10 | rs10822054 | 3,6770E-05 | intergene |
| Grünewald TG., 2015 | Nat Genet. | ADO, EGR2 | 10 | rs10822056 | 3,6950E-06 | intergene |
| Grünewald TG., 2015 | Nat Genet. | ZNF365, ADO, EGR2 | 10 | rs10822057 | 6,6620E-05 | intergene |
| Grünewald TG., 2015 | Nat Genet. | ZNF365, ADO, EGR2 | 10 | rs10822058 | 5,7860E-05 | intergene |
| Grünewald TG., 2015 | Nat Genet. | ZNF365, ADO, EGR2 | 10 | rs10822059 | 1,6870E-04 | intergene |
| Grünewald TG., 2015 | Nat Genet. | EGR2, ADO, LOC107984012 | 10 | rs10822060 | 1,2950E-04 | intergene |
| Grünewald TG., 2015 | Nat Genet. | EGR2, ADO, LOC107984012 | 10 | rs10822062 | 2,9590E-03 | intergene |
| Grünewald TG., 2015 | Nat Genet. | EGR2, ADO, LOC107984012 | 10 | rs10822065 | 2,9720E-03 | intergene |
| Grünewald TG., 2015 | Nat Genet. | EGR2, ADO, LOC107984012 | 10 | rs10822066 | 1,1830E-03 | intergene |
| Grünewald TG., 2015 | Nat Genet. | EGR2, ADO, LOC107984012 | 10 | rs10822067 | 9,9440E-04 | intergene |
| Grünewald TG., 2015 | Nat Genet. | EGR2, ADO, LOC107984012 | 10 | rs10822068 | 9,9440E-04 | intergene |
| Grünewald TG., 2015 | Nat Genet. | EGR2, ADO, LOC107984012 | 10 | rs10822069 | 7,9820E-04 | intergene |
| Grünewald TG., 2015 | Nat Genet. | EGR2, ADO, LOC107984012 | 10 | rs10822070 | 7,9820E-04 | intergene |
| Grünewald TG., 2015 | Nat Genet. | EGR2, ADO, LOC107984012 | 10 | rs10822071 | 9,5120E-04 | intergene |
| Grünewald TG., 2015 | Nat Genet. | EGR2, ADO, LOC107984012 | 10 | rs10822072 | 1,1080E-03 | intergene |
| Grünewald TG., 2015 | Nat Genet. | EGR2, ADO, LOC107984012 | 10 | rs10822075 | 4,1240E-03 | intergene |
| Grünewald TG., 2015 | Nat Genet. | LOC107984012 | 10 | rs10822078 | 2,3980E-03 | intergene |
| Grünewald TG., 2015 | Nat Genet. | LOC107984012 | 10 | rs10822079 | 1,3690E-05 | intergene |
| Grünewald TG., 2015 | Nat Genet. | LOC107984012 | 10 | rs10822083 | 4,5540E-03 | intergene |
| Grünewald TG., 2015 | Nat Genet. | LOC107984012 | 10 | rs10822086 | 5,5030E-03 | intergene |
| Grünewald TG., 2015 | Nat Genet. | LOC107984012 | 10 | rs10822087 | 6,4020E-03 | intergene |
| Grünewald TG., 2015 | Nat Genet. | LOC107984012 | 10 | rs10822091 | 6,4020E-03 | intergene |
| Grünewald TG., 2015 | Nat Genet. | LOC107984012 | 10 | rs10822092 | 5,3830E-03 | intergene |
| Grünewald TG., 2015 | Nat Genet. | LOC107984012, NRBF2 | 10 | rs10822102 | 3,1340E-03 | intergene |
| Savage SA., 2013 | Nat Genet. | TRMT12 | 8 | rs10956183 | 0,0023 | intergene |
| Grünewald TG., 2015 | Nat Genet. | ZNF365 | 10 | rs10995276 | 3,4120E-05 | intergene |
| Grünewald TG., 2015 | Nat Genet. | ZNF365 | 10 | rs10995278 | 3,4120E-05 | intergene |
| Grünewald TG., 2015 | Nat Genet. | ZNF365 | 10 | rs10995281 | 4,8490E-05 | intergene |
| Grünewald TG., 2015 | Nat Genet. | ZNF365 | 10 | rs10995282 | 4,7910E-05 | intergene |
| Grünewald TG., 2015 | Nat Genet. | ZNF365 | 10 | rs10995294 | 3,0630E-05 | intergene |
| Grünewald TG., 2015 | Nat Genet. | ZNF365, ADO, EGR2 | 10 | rs10995299 | 4,9360E-05 | intergene |
| Grünewald TG., 2015 | Nat Genet. | ADO, EGR2 | 10 | rs10995305 | 4,3830E-07 | intergene |
| Grünewald TG., 2015 | Nat Genet. | ADO, EGR2 | 10 | rs10995307 | 5,7980E-05 | intergene |
| Grünewald TG., 2015 | Nat Genet. | EGR2, ADO, LOC107984012 | 10 | rs10995324 | 5,6220E-05 | intergene |
| Grünewald TG., 2015 | Nat Genet. | EGR2, ADO, LOC107984012 | 10 | rs10995327 | 1,1080E-03 | intergene |
| Grünewald TG., 2015 | Nat Genet. | EGR2, ADO, LOC107984012 | 10 | rs10995328 | 8,7240E-05 | intergene |
| Grünewald TG., 2015 | Nat Genet. | EGR2, ADO, LOC107984012 | 10 | rs10995331 | 9,9440E-04 | intergene |
| Grünewald TG., 2015 | Nat Genet. | LOC107984012 | 10 | rs10995344 | 7,4480E-04 | intergene |
| Grünewald TG., 2015 | Nat Genet. | LOC107984012 | 10 | rs10995376 | 6,5620E-03 | intergene |
| Grünewald TG., 2015 | Nat Genet. | LOC107984012 | 10 | rs10995377 | 6,5620E-03 | intergene |
| Grünewald TG., 2015 | Nat Genet. | LOC107984012 | 10 | rs10995378 | 6,4070E-03 | intergene |
| Savage SA., 2013 | Nat Genet. | MAFA, ZC3H3 | 8 | rs11136295 | 0,005 | intergene |
| Grünewald TG., 2015 | Nat Genet. | EGR2, LOC107984012 | 10 | rs1115705 | 2,7280E-06 | intergene |
| Savage SA., 2013 | Nat Genet. | LOC101927822 | 8 | rs1125719 | 0,0074 | intergene |
| Grünewald TG., 2015 | Nat Genet. | ZNF365, ADO, EGR2 | 10 | rs11592442 | 1,7260E-05 | intergene |
| Grünewald TG., 2015 | Nat Genet. | ZNF365, ADO, EGR2 | 10 | rs11596699 | 4,5540E-03 | intergene |
| Grünewald TG., 2015 | Nat Genet. | ZNF365, ADO, EGR2 | 10 | rs11597299 | 4,7920E-05 | intergene |
| Grünewald TG., 2015 | Nat Genet. | ZNF365, ADO | 10 | rs11599754 | 5,7340E-05 | intergene |
| Postel-Vinay S., 2012 | Nat Genet. | ZNF365, ADO | 10 | rs11599754 | 2,20E-11 | intergene |
| Savage SA., 2013 | Nat Genet. | LOC642635, LOC107985951 | 2 | rs11673716 | 2,06E-05 | intergene |
| Grünewald TG., 2015 | Nat Genet. | LOC107984012 | 10 | rs117066695 | 3,6930E-03 | intergene |
| Savage SA., 2013 | Nat Genet. | COL22A1, KCNK9 | 8 | rs11775898 | 0,0352 | intergene |
| Savage SA., 2013 | Nat Genet. | LOC107986905 | 8 | rs11780467 | 0,012 | intergene |
| Grünewald TG., 2015 | Nat Genet. | ZNF365 | 10 | rs11815005 | 6,6680E-05 | intergene |
| Mirabello L., 2011 | BMC Cancer | FANCL, LOC105374744 | 2 | rs11894186 | 0,0307 | intergene |
| Savage SA., 2013 | Nat Genet. | COL22A1, KCNK9 | 8 | rs11997682 | 0,0451 | intergene |
| Savage SA., 2013 | Nat Genet. | KHDRBS3, LOC107986978 | 8 | rs12056444 | 0,0077 | intergene |
| Mirabello L., 2011 | BMC Cancer | IGF2R | 6 | rs12202350 | 0,0245 | intergene |
| Savage SA., 2013 | Nat Genet. | MROH5, LOC105375791 | 8 | rs12547611 | 0,0367 | intergene |
| Mirabello L., 2011 | BMC Cancer | FGFR2, LOC107984183 | 10 | rs12572779 | 0,0260 | intergene |
| Savage SA., 2013 | Nat Genet. | LOC105375792, TSNARE1 | 8 | rs12676273 | 0,0306 | intergene |
| Savage SA., 2013 | Nat Genet. | LOC101927822 | 8 | rs12677718 | 0,0359 | intergene |
| Mirabello L., 2011 | BMC Cancer | IGFBP2, LINC01280 | 2 | rs12694392 | 0,0202 | intergene |
| Mirabello L., 2011 | BMC Cancer | BMP6, LOC105374906 | 6 | rs1322239 | 0,0303 | intergene |
| Savage SA., 2013 | Nat Genet. | MROH5, LOC105375789 | 8 | rs13248484 | 0,0254 | intergene |
| Savage SA., 2013 | Nat Genet. | COL22A1, KCNK9 | 8 | rs13259487 | 0,0465 | intergene |
| Savage SA., 2013 | Nat Genet. | ST3GAL1, LOC105375773 | 8 | rs13264573 | 0,0402 | intergene |
| Savage SA., 2013 | Nat Genet. | LOC101927822 | 8 | rs13267601 | 0,0428 | intergene |
| Savage SA., 2013 | Nat Genet. | EXT1, SAMD12 | 8 | rs13271779 | 0,0452 | intergene |
| Savage SA., 2013 | Nat Genet. | ZFAT, LOC102723694 | 8 | rs13272667 | 0,0276 | intergene |
| Savage SA., 2013 | Nat Genet. | TNFRSF11B, COLEC10, LOC105375723 | 8 | rs13279492 | 0,0467 | intergene |
| Savage SA., 2013 | Nat Genet. | EIF3H, UTP23, RAD21 | 8 | rs13279543 | 0,0049 | intergene |
| Grünewald TG., 2015 | Nat Genet. | LOC107984012 | 10 | rs1332532 | 1,2710E-05 | intergene |
| Savage SA., 2013 | Nat Genet. | LOC105373401, LOC105373402 | 2 | rs13403411 | 5,20E-06 | intergene |
| Savage SA., 2013 | Nat Genet. | LOC101927822 | 8 | rs1354365 | 0,0012 | intergene |
| Savage SA., 2013 | Nat Genet. | LINC00824, LINC00977 | 8 | rs1356761 | 0,0179 | intergene |
| Grünewald TG., 2015 | Nat Genet. | LOC107984012, NRBF2 | 10 | rs1397028 | 5,8930E-03 | intergene |
| Grünewald TG., 2015 | Nat Genet. | EGR2, ADO, LOC107984012 | 10 | rs1397030 | 8,7240E-05 | intergene |
| Grünewald TG., 2015 | Nat Genet. | EGR2, ADO, LOC107984013 | 10 | rs1412554 | 9,9380E-04 | intergene |
| Savage SA., 2013 | Nat Genet. | LOC105375760, LOC105375763, LOC107986976 | 8 | rs1420484 | 0,0061 | intergene |
| Grünewald TG., 2015 | Nat Genet. | EGR2, ADO, LOC107984012 | 10 | rs1475082 | 9,5120E-04 | intergene |
| Grünewald TG., 2015 | Nat Genet. | EGR2, ADO, LOC107984012 | 10 | rs1475083 | 7,9820E-04 | intergene |
| Grünewald TG., 2015 | Nat Genet. | EGR2, ADO, LOC107984012 | 10 | rs1475084 | 7,9820E-04 | intergene |
| Grünewald TG., 2015 | Nat Genet. | EGR2, LOC107984012 | 10 | rs1509952 | 5,2780E-06 | intergene |
| Grünewald TG., 2015 | Nat Genet. | EGR2, ADO, LOC107984012 | 10 | rs1509955 | 5,6220E-05 | intergene |
| Grünewald TG., 2015 | Nat Genet. | EGR2, ADO, LOC107984012 | 10 | rs1509956 | 7,9830E-04 | intergene |
| Grünewald TG., 2015 | Nat Genet. | EGR2, ADO, LOC107984012 | 10 | rs1509957 | 7,9820E-04 | intergene |
| Grünewald TG., 2015 | Nat Genet. | EGR2, ADO, LOC107984012 | 10 | rs1509958 | 1,5780E-03 | intergene |
| Grünewald TG., 2015 | Nat Genet. | LOC107984012 | 10 | rs1509961 | 5,1890E-03 | intergene |
| Grünewald TG., 2015 | Nat Genet. | LOC107984012 | 10 | rs1509962 | 4,7840E-03 | intergene |
| Grünewald TG., 2015 | Nat Genet. | ADO, EGR2 | 10 | rs1509966 | 6,1820E-05 | intergene |
| Postel-Vinay S., 2012 | Nat Genet. | ADO, EGR2 | 10 | rs1509966 | 2,90E-15 | intergene |
| Savage SA., 2013 | Nat Genet. | KCNQ3, LOC105375765, LRRC6 | 8 | rs1515509 | 0,0125 | intergene |
| Savage SA., 2013 | Nat Genet. | LOC105375792, TSNARE1 | 8 | rs1542758 | 0,0262 | intergene |
| Grünewald TG., 2015 | Nat Genet. | EGR2, ADO, LOC107984012 | 10 | rs1546479 | 2,6560E-04 | intergene |
| Grünewald TG., 2015 | Nat Genet. | EGR2, ADO, LOC107984012 | 10 | rs1546480 | 1,3260E-04 | intergene |
| Grünewald TG., 2015 | Nat Genet. | EGR2, LOC107984012 | 10 | rs1571918 | 3,4370E-06 | intergene |
| Grünewald TG., 2015 | Nat Genet. | EGR2, ADO, LOC107984012 | 10 | rs1571919 | 4,1710E-03 | intergene |
| Grünewald TG., 2015 | Nat Genet. | EGR2, ADO, LOC107984012 | 10 | rs1571921 | 7,1650E-05 | intergene |
| Grünewald TG., 2015 | Nat Genet. | EGR2, ADO, LOC107984012 | 10 | rs1571922 | 9,9440E-04 | intergene |
| Savage SA., 2013 | Nat Genet. | GSDMC, CCDC26 | 8 | rs16904143 | 0,0397 | intergene |
| Savage SA., 2013 | Nat Genet. | LOC105375763, LOC107986976, EFR3A | 8 | rs16904505 | 0,0149 | intergene |
| Savage SA., 2013 | Nat Genet. | ST3GAL1, LOC105375773 | 8 | rs16904960 | 0,0213 | intergene |
| Savage SA., 2013 | Nat Genet. | LOC105375775, LOC107986979 | 8 | rs16905528 | 0,0384 | intergene |
| Savage SA., 2013 | Nat Genet. | LOC107986905 | 8 | rs16906838 | 0,0058 | intergene |
| Grünewald TG., 2015 | Nat Genet. | EGR2, ADO, LOC107984025 | 10 | rs16917768 | 4,6910E-05 | intergene |
| Mirabello L., 2011 | BMC Cancer | SDR42E1, HSD17B2 | 16 | rs16956238 | 0,0217 | intergene |
| Mirabello L., 2011 | BMC Cancer | HSD17B2 | 16 | rs16956419 | 0,0497 | intergene |
| Mirabello L., 2011 | BMC Cancer | NOG | 17 | rs16957413 | 0,0211 | intergene |
| Savage SA., 2013 | Nat Genet. | SNTB1 | 8 | rs17196271 | 0,0235 | intergene |
| DuBois SG., 2012 | Pediatr Blood Cancer | EWSR1, GAS2L1 | 22 | rs174760 | 0,7627 | intergene |
| Mirabello L., 2011 | BMC Cancer | NOG | 17 | rs17822219 | 0,0312 | intergene |
| Grünewald TG., 2015 | Nat Genet. | EGR2, ADO, LOC107984012 | 10 | rs1831643 | 1,2260E-03 | intergene |
| Grünewald TG., 2015 | Nat Genet. | EGR2, ADO, LOC107984012 | 10 | rs1831644 | 1,1900E-03 | intergene |
| Grünewald TG., 2015 | Nat Genet. | EGR2, ADO | 10 | rs1848796 | 1,0750E-06 | intergene |
| Grünewald TG., 2015 | Nat Genet. | ADO, EGR2 | 10 | rs1848797 | 8,7980E-06 | intergene |
| Postel-Vinay S., 2012 | Nat Genet. | ADO, EGR2 | 10 | rs1848797 | 9,20E-15 | intergene |
| Savage SA., 2013 | Nat Genet. | COL22A1 | 8 | rs1876201 | 0,0084 | intergene |
| Grünewald TG., 2015 | Nat Genet. | EGR2, LOC107984012 | 10 | rs1888968 | 3,4370E-06 | intergene |
| Savage SA., 2013 | Nat Genet. | KCNQ3, LOC105375765, LRRC6 | 8 | rs1901090 | 0,0112 | intergene |
| Grünewald TG., 2015 | Nat Genet. | ZNF365, ADO, EGR2 | 10 | rs190113 | 7,7770E-04 | intergene |
| Grünewald TG., 2015 | Nat Genet. | EGR2, LOC107984012 | 10 | rs1912369 | 3,4370E-06 | intergene |
| Grünewald TG., 2015 | Nat Genet. | ZNF365, ADO, EGR2 | 10 | rs192004 | 1,1850E-05 | intergene |
| Grünewald TG., 2015 | Nat Genet. | EGR2, ADO, LOC107984012 | 10 | rs192007 | 5,7620E-05 | intergene |
| Grünewald TG., 2015 | Nat Genet. | EGR2, ADO, LOC107984012 | 10 | rs1967747 | 7,9820E-04 | intergene |
| Savage SA., 2013 | Nat Genet. | KCNK9, LOC105375777, TRAPPC9 | 8 | rs199219 | 0,0183 | intergene |
| Grünewald TG., 2015 | Nat Genet. | LOC107984012 | 10 | rs1999477 | 1,0520E-05 | intergene |
| Grünewald TG., 2015 | Nat Genet. | EGR2, ADO, LOC107984012 | 10 | rs200008082 | 9,9000E-04 | intergene |
| Tie Z., 2014 | Int J Clin Exp Pathol. | LOC105374054 | 6 | rs2010965 | 0,0038 | intergene |
| Grünewald TG., 2015 | Nat Genet. | LOC107984012 | 10 | rs2026407 | 1,3210E-05 | intergene |
| Grünewald TG., 2015 | Nat Genet. | LOC107984012 | 10 | rs2026408 | 9,2460E-03 | intergene |
| Savage SA., 2013 | Nat Genet. | MROH5, LOC105375791 | 8 | rs2085841 | 0,0385 | intergene |
| Martinelli M., 2016 | Oncotarget | CD99 | Y | rs2109560 | 0,5437 | intergene |
| Savage SA., 2013 | Nat Genet. | KCNK9 | 8 | rs2111573 | 0,0477 | intergene |
| Mirabello L., 2011 | BMC Cancer | GDF2, RBP3 | 10 | rs2125064 | 0,0346 | intergene |
| Savage SA., 2013 | Nat Genet. | COL22A1 | 8 | rs2135455 | 0,0034 | intergene |
| Grünewald TG., 2015 | Nat Genet. | EGR2, ADO, LOC107984012 | 10 | rs2136613 | 5,1520E-05 | intergene |
| Savage SA., 2013 | Nat Genet. | KCNQ3, LOC105375765, LRRC6 | 8 | rs2138248 | 0,0318 | intergene |
| Grünewald TG., 2015 | Nat Genet. | ZNF365, ADO, EGR2 | 10 | rs224028 | 1,4370E-05 | intergene |
| Grünewald TG., 2015 | Nat Genet. | ZNF365, ADO, EGR2 | 10 | rs224029 | 1,7840E-05 | intergene |
| Grünewald TG., 2015 | Nat Genet. | ZNF365, ADO, EGR2 | 10 | rs224030 | 2,7550E-05 | intergene |
| Grünewald TG., 2015 | Nat Genet. | ZNF365, ADO, EGR2 | 10 | rs224031 | 6,7470E-04 | intergene |
| Grünewald TG., 2015 | Nat Genet. | ZNF365, ADO, EGR2 | 10 | rs224032 | 6,7470E-04 | intergene |
| Grünewald TG., 2015 | Nat Genet. | ZNF365, ADO, EGR2 | 10 | rs224033 | 6,7470E-04 | intergene |
| Grünewald TG., 2015 | Nat Genet. | ZNF365, ADO, EGR2 | 10 | rs224042 | 2,4950E-05 | intergene |
| Grünewald TG., 2015 | Nat Genet. | ZNF365, ADO, EGR2 | 10 | rs224043 | 1,8040E-05 | intergene |
| Grünewald TG., 2015 | Nat Genet. | ZNF365, ADO, EGR2 | 10 | rs224044 | 1,4120E-05 | intergene |
| Grünewald TG., 2015 | Nat Genet. | ZNF365, ADO, EGR2 | 10 | rs224045 | 1,0110E-05 | intergene |
| Grünewald TG., 2015 | Nat Genet. | ZNF365, ADO, EGR2 | 10 | rs224046 | 2,0700E-04 | intergene |
| Grünewald TG., 2015 | Nat Genet. | ZNF365, ADO, EGR2 | 10 | rs224048 | 4,3980E-04 | intergene |
| Grünewald TG., 2015 | Nat Genet. | ZNF365, ADO, EGR2 | 10 | rs224049 | 3,6770E-05 | intergene |
| Grünewald TG., 2015 | Nat Genet. | ZNF365, ADO, EGR2 | 10 | rs224050 | 4,7220E-05 | intergene |
| Grünewald TG., 2015 | Nat Genet. | ZNF365, ADO, EGR2 | 10 | rs224052 | 7,7770E-04 | intergene |
| Grünewald TG., 2015 | Nat Genet. | ZNF365, ADO, EGR2 | 10 | rs224055 | 1,1850E-05 | intergene |
| Grünewald TG., 2015 | Nat Genet. | ZNF365, ADO, EGR2 | 10 | rs224056 | 1,1850E-05 | intergene |
| Grünewald TG., 2015 | Nat Genet. | ZNF365, ADO, EGR2 | 10 | rs224057 | 4,3980E-04 | intergene |
| Grünewald TG., 2015 | Nat Genet. | ZNF365, ADO, EGR2 | 10 | rs224058 | 7,7770E-04 | intergene |
| Grünewald TG., 2015 | Nat Genet. | ZNF365, ADO, EGR2 | 10 | rs224061 | 2,0400E-05 | intergene |
| Grünewald TG., 2015 | Nat Genet. | ZNF365, ADO, EGR2 | 10 | rs224062 | 3,5940E-05 | intergene |
| Grünewald TG., 2015 | Nat Genet. | ZNF365, ADO, EGR2 | 10 | rs224063 | 1,5350E-05 | intergene |
| Grünewald TG., 2015 | Nat Genet. | ZNF365, ADO, EGR2 | 10 | rs224064 | 3,3240E-05 | intergene |
| Grünewald TG., 2015 | Nat Genet. | ZNF365, ADO, EGR2 | 10 | rs224065 | 1,1850E-05 | intergene |
| Grünewald TG., 2015 | Nat Genet. | ZNF365, ADO, EGR2 | 10 | rs224066 | 1,1850E-05 | intergene |
| Grünewald TG., 2015 | Nat Genet. | ZNF365, ADO, EGR2 | 10 | rs224067 | 1,1850E-05 | intergene |
| Grünewald TG., 2015 | Nat Genet. | ZNF365, ADO, EGR2 | 10 | rs224068 | 1,3860E-05 | intergene |
| Grünewald TG., 2015 | Nat Genet. | ZNF365, ADO, EGR2 | 10 | rs224069 | 3,3340E-05 | intergene |
| Grünewald TG., 2015 | Nat Genet. | ZNF365, ADO, EGR2 | 10 | rs224070 | 1,2330E-05 | intergene |
| Grünewald TG., 2015 | Nat Genet. | ZNF365, ADO, EGR2 | 10 | rs224071 | 5,0060E-04 | intergene |
| Grünewald TG., 2015 | Nat Genet. | ZNF365, ADO, EGR2 | 10 | rs224072 | 3,3340E-05 | intergene |
| Grünewald TG., 2015 | Nat Genet. | ZNF365, ADO, EGR2 | 10 | rs224073 | 2,5280E-05 | intergene |
| Grünewald TG., 2015 | Nat Genet. | ZNF365, ADO, EGR2 | 10 | rs224074 | 2,2590E-05 | intergene |
| Grünewald TG., 2015 | Nat Genet. | ZNF365, ADO, EGR2 | 10 | rs224076 | 2,4270E-05 | intergene |
| Grünewald TG., 2015 | Nat Genet. | ZNF365, ADO, EGR2 | 10 | rs224077 | 2,4270E-05 | intergene |
| Grünewald TG., 2015 | Nat Genet. | ZNF365, ADO, EGR2 | 10 | rs224078 | 3,5220E-05 | intergene |
| Grünewald TG., 2015 | Nat Genet. | ZNF365, ADO, EGR2 | 10 | rs224079 | 9,2350E-06 | intergene |
| Grünewald TG., 2015 | Nat Genet. | ZNF365, ADO, EGR2 | 10 | rs224090 | 4,7970E-05 | intergene |
| Grünewald TG., 2015 | Nat Genet. | ZNF365, ADO, EGR2 | 10 | rs224091 | 8,0570E-03 | intergene |
| Grünewald TG., 2015 | Nat Genet. | ZNF365, ADO, EGR2 | 10 | rs224092 | 4,7970E-05 | intergene |
| Grünewald TG., 2015 | Nat Genet. | ZNF365, ADO, EGR2 | 10 | rs224109 | 4,7970E-05 | intergene |
| Grünewald TG., 2015 | Nat Genet. | ZNF365, ADO, EGR2 | 10 | rs224111 | 1,9980E-05 | intergene |
| Grünewald TG., 2015 | Nat Genet. | ZNF365, ADO, EGR2 | 10 | rs224123 | 3,5730E-05 | intergene |
| Grünewald TG., 2015 | Nat Genet. | ZNF365, ADO, EGR2 | 10 | rs224125 | 2,4470E-05 | intergene |
| Grünewald TG., 2015 | Nat Genet. | ZNF365, ADO, EGR2 | 10 | rs224127 | 4,5270E-05 | intergene |
| Grünewald TG., 2015 | Nat Genet. | ZNF365, ADO, EGR2 | 10 | rs224130 | 3,5700E-05 | intergene |
| Grünewald TG., 2015 | Nat Genet. | ZNF365, ADO, EGR2 | 10 | rs224131 | 3,5700E-05 | intergene |
| Grünewald TG., 2015 | Nat Genet. | ZNF365, ADO, EGR2 | 10 | rs224133 | 3,5700E-05 | intergene |
| Grünewald TG., 2015 | Nat Genet. | ZNF365, ADO, EGR2 | 10 | rs224134 | 4,5090E-05 | intergene |
| Grünewald TG., 2015 | Nat Genet. | ZNF365, ADO, EGR2 | 10 | rs224135 | 4,5080E-05 | intergene |
| Grünewald TG., 2015 | Nat Genet. | ZNF365, ADO, EGR2 | 10 | rs224143 | 5,8590E-05 | intergene |
| Grünewald TG., 2015 | Nat Genet. | ZNF365, ADO, EGR2 | 10 | rs224145 | 3,5700E-05 | intergene |
| Grünewald TG., 2015 | Nat Genet. | ZNF365, ADO, EGR2 | 10 | rs224146 | 2,3730E-05 | intergene |
| Grünewald TG., 2015 | Nat Genet. | ZNF365, ADO, EGR2 | 10 | rs224147 | 2,1680E-05 | intergene |
| Grünewald TG., 2015 | Nat Genet. | ZNF365, ADO, EGR2 | 10 | rs224148 | 7,7770E-04 | intergene |
| Grünewald TG., 2015 | Nat Genet. | ZNF365, ADO, EGR2 | 10 | rs224149 | 7,7770E-04 | intergene |
| Grünewald TG., 2015 | Nat Genet. | ZNF365, ADO, EGR2 | 10 | rs224150 | 7,7770E-04 | intergene |
| Grünewald TG., 2015 | Nat Genet. | ADO, EGR2 | 10 | rs224282 | 1,0750E-06 | intergene |
| Grünewald TG., 2015 | Nat Genet. | ZNF365, ADO, EGR2 | 10 | rs224285 | 4,4600E-04 | intergene |
| Grünewald TG., 2015 | Nat Genet. | ADO, EGR2 | 10 | rs224289 | 1,4150E-06 | intergene |
| Grünewald TG., 2015 | Nat Genet. | ADO, EGR2 | 10 | rs224290 | 7,7950E-07 | intergene |
| Grünewald TG., 2015 | Nat Genet. | ADO, EGR2 | 10 | rs224291 | 7,7950E-07 | intergene |
| Grünewald TG., 2015 | Nat Genet. | ADO, EGR2 | 10 | rs224292 | 4,3800E-05 | intergene |
| Postel-Vinay S., 2012 | Nat Genet. | ADO, EGR2 | 10 | rs224292 | 1,20E-08 | intergene |
| Grünewald TG., 2015 | Nat Genet. | ADO, EGR2 | 10 | rs224293 | 1,0200E-06 | intergene |
| Grünewald TG., 2015 | Nat Genet. | ADO, EGR2 | 10 | rs224294 | 1,0100E-06 | intergene |
| Grünewald TG., 2015 | Nat Genet. | ADO, EGR2 | 10 | rs224295 | 4,7970E-06 | intergene |
| Grünewald TG., 2015 | Nat Genet. | ADO, EGR2 | 10 | rs224296 | 7,7950E-07 | intergene |
| Grünewald TG., 2015 | Nat Genet. | ADO, EGR2 | 10 | rs224297 | 7,7950E-07 | intergene |
| Grünewald TG., 2015 | Nat Genet. | ADO, EGR2 | 10 | rs224298 | 7,7950E-07 | intergene |
| Grünewald TG., 2015 | Nat Genet. | ADO, EGR2 | 10 | rs224299 | 4,7970E-06 | intergene |
| Grünewald TG., 2015 | Nat Genet. | ADO, EGR2 | 10 | rs224301 | 3,6700E-06 | intergene |
| Grünewald TG., 2015 | Nat Genet. | ADO, EGR2 | 10 | rs224302 | 3,6700E-06 | intergene |
| Grünewald TG., 2015 | Nat Genet. | ZNF365, ADO, EGR2 | 10 | rs224307 | 5,6220E-05 | intergene |
| Grünewald TG., 2015 | Nat Genet. | ZNF365, ADO, EGR2 | 10 | rs224308 | 8,7240E-05 | intergene |
| Grünewald TG., 2015 | Nat Genet. | ZNF365, ADO, EGR2 | 10 | rs224309 | 8,9300E-05 | intergene |
| Grünewald TG., 2015 | Nat Genet. | ZNF365, ADO, EGR2 | 10 | rs224310 | 8,9650E-05 | intergene |
| Grünewald TG., 2015 | Nat Genet. | ZNF365, ADO, EGR2 | 10 | rs224311 | 8,3640E-05 | intergene |
| Grünewald TG., 2015 | Nat Genet. | ZNF365, ADO, EGR2 | 10 | rs224313 | 5,5480E-05 | intergene |
| Savage SA., 2013 | Nat Genet. | COL22A1 | 8 | rs2318832 | 0,0024 | intergene |
| Savage SA., 2013 | Nat Genet. | MAL2, NOV | 8 | rs2326244 | 0,0358 | intergene |
| Cong Y., 2014 | Tumor Biol. | BMP2 | 20 | rs235770 | 0,9195 | intergene |
| Grünewald TG., 2015 | Nat Genet. | EGR2, ADO, LOC107984012 | 10 | rs2393933 | 1,2570E-05 | intergene |
| Savage SA., 2013 | Nat Genet. | MAL2, NOV | 8 | rs2447186 | 0,0281 | intergene |
| Savage SA., 2013 | Nat Genet. | LOC105375752 | 8 | rs2466024 | 0,0204 | intergene |
| Savage SA., 2013 | Nat Genet. | LOC105375752 | 8 | rs2466035 | 0,0087 | intergene |
| Savage SA., 2013 | Nat Genet. | SLC8A1-AS1 | 2 | rs2888638 | 1,03E-05 | intergene |
| Savage SA., 2013 | Nat Genet. | ST3GAL1, LOC105375773 | 8 | rs2978056 | 0,0092 | intergene |
| Mirabello L., 2011 | BMC Cancer | KIAA1468, TNFRSF11A | 18 | rs2981006 | 0,0148 | intergene |
| Martinelli M., 2016 | Oncotarget | CD99 | Y | rs311097 | 0,3356 | intergene |
| Martinelli M., 2016 | Oncotarget | CD99 | Y | rs311100 | 0,8258 | intergene |
| Savage SA., 2013 | Nat Genet. | NOV | 8 | rs34112166 | 0,0387 | intergene |
| Grünewald TG., 2015 | Nat Genet. | ZNF365, ADO, EGR2 | 10 | rs377859 | 2,3980E-05 | intergene |
| Grünewald TG., 2015 | Nat Genet. | ZNF365, ADO, EGR2 | 10 | rs382412 | 3,6720E-05 | intergene |
| Grünewald TG., 2015 | Nat Genet. | ZNF365, ADO, EGR2 | 10 | rs382424 | 3,6720E-05 | intergene |
| Savage SA., 2013 | Nat Genet. | ST3GAL1, LOC105375773 | 8 | rs3958181 | 0,0332 | intergene |
| Mirabello L., 2011 | BMC Cancer | ENDOV, NPTX1 | 17 | rs4074302 | 0,0214 | intergene |
| Mirabello L., 2011 | BMC Cancer | ENDOV, NPTX1 | 17 | rs4074303 | 0,0090 | intergene |
| Grünewald TG., 2015 | Nat Genet. | EGR2, ADO, LOC107984012 | 10 | rs4141614 | 5,6220E-05 | intergene |
| Grünewald TG., 2015 | Nat Genet. | EGR2, ADO, LOC107984012 | 10 | rs4141617 | 9,9440E-04 | intergene |
| Grünewald TG., 2015 | Nat Genet. | EGR2, LOC107984012 | 10 | rs4147153 | 3,4370E-06 | intergene |
| Grünewald TG., 2015 | Nat Genet. | EGR2, ADO, LOC107984012 | 10 | rs4237315 | 6,4020E-03 | intergene |
| Grünewald TG., 2015 | Nat Genet. | EGR2, LOC107984012 | 10 | rs4237316 | 3,4370E-06 | intergene |
| Savage SA., 2013 | Nat Genet. | RHPN1, ZC3H3 | 8 | rs4336628 | 0,0367 | intergene |
| Grünewald TG., 2015 | Nat Genet. | EGR2, ADO, LOC107984012 | 10 | rs4400683 | 3,1340E-03 | intergene |
| Mirabello L., 2011 | BMC Cancer | HSD17B2, SDR42E1 |  | rs4404064 | 0,0273 | intergene |
| Grünewald TG., 2015 | Nat Genet. | EGR2, ADO, LOC107984012 | 10 | rs4414110 | 2,9370E-04 | intergene |
| Grünewald TG., 2015 | Nat Genet. | EGR2, ADO, LOC107984012 | 10 | rs442309 | 6,2080E-04 | intergene |
| Grünewald TG., 2015 | Nat Genet. | EGR2, ADO, LOC107984012 | 10 | rs448355 | 7,9580E-05 | intergene |
| Grünewald TG., 2015 | Nat Genet. | EGR2, ADO, LOC107984012 | 10 | rs4523588 | 4,3460E-03 | intergene |
| Savage SA., 2013 | Nat Genet. | LOC105375754 | 8 | rs4551307 | 3,59E-05 | intergene |
| Savage SA., 2013 | Nat Genet. | LOC105375754 | 8 | rs4551307 | 8,22E-04 | intergene |
| Savage SA., 2013 | Nat Genet. | TMEM65, TRMT12 | 8 | rs4620270 | 0,0117 | intergene |
| Savage SA., 2013 | Nat Genet. | GSDMC | 8 | rs4733723 | 0,049 | intergene |
| Mirabello L., 2011 | BMC Cancer | CYP11B1 | 8 | rs4736349 | 0,0225 | intergene |
| Savage SA., 2013 | Nat Genet. | NDRG1, LOC105375771 | 8 | rs4736648 | 0,0439 | intergene |
| Grünewald TG., 2015 | Nat Genet. | LOC107984012 | 10 | rs4746728 | 7,6460E-03 | intergene |
| Grünewald TG., 2015 | Nat Genet. | EGR2, LOC107984012 | 10 | rs4746745 | 3,4820E-06 | intergene |
| Grünewald TG., 2015 | Nat Genet. | EGR2, LOC107984012 | 10 | rs4746746 | 3,4370E-06 | intergene |
| Grünewald TG., 2015 | Nat Genet. | LOC107984012 | 10 | rs4746747 | 2,0040E-05 | intergene |
| Grünewald TG., 2015 | Nat Genet. | LOC107984012 | 10 | rs4746783 | 3,1340E-03 | intergene |
| Savage SA., 2013 | Nat Genet. | UTP23, RAD21 | 8 | rs4876683 | 0,0414 | intergene |
| Mirabello L., 2011 | BMC Cancer | CTGF | 6 | rs4897555 | 0,0163 | intergene |
| Savage SA., 2013 | Nat Genet. | MROH5, LOC105375791 | 8 | rs4907333 | 0,0399 | intergene |
| Savage SA., 2013 | Nat Genet. | KHDRBS3, LOC107986978 | 8 | rs4909528 | 0,032 | intergene |
| Savage SA., 2013 | Nat Genet. | LOC107986978, LOC107986980 | 8 | rs4909593 | 0,026 | intergene |
| Savage SA., 2013 | Nat Genet. | FAM135B, COL22A1 | 8 | rs4909806 | 0,0041 | intergene |
| Mirabello L., 2011 | BMC Cancer | PARP2, TEP1 | 14 | rs4981998 | 0,0468 | intergene |
| Savage SA., 2013 | Nat Genet. | DLEU1, LOC107984568 | 13 | rs573666 | 8,59E-06 | intergene |
| Grünewald TG., 2015 | Nat Genet. | EGR2, ADO, LOC107984012 | 10 | rs58329391 | 9,7070E-03 | intergene |
| Martinelli M., 2016 | Oncotarget | CD99, XG | Y | rs5939312 | 0,7663 | intergene |
| Mirabello L., 2011 | BMC Cancer | GNRH2, MRPS26 | 20 | rs6076466 | 0,0304 | intergene |
| Grünewald TG., 2015 | Nat Genet. | EGR2, ADO, LOC107984012 | 10 | rs61863928 | 2,9050E-04 | intergene |
| Savage SA., 2013 | Nat Genet. | ST3GAL1, LOC105375773 | 8 | rs6471136 | 0,0303 | intergene |
| Grünewald TG., 2015 | Nat Genet. | EGR2, ADO, LOC107984012 | 10 | rs6479842 | 1,3210E-05 | intergene |
| Grünewald TG., 2015 | Nat Genet. | EGR2, LOC107984012 | 10 | rs6479854 | 3,4370E-06 | intergene |
| Grünewald TG., 2015 | Nat Genet. | EGR2, ADO, LOC107984012 | 10 | rs6479855 | 2,9370E-04 | intergene |
| Grünewald TG., 2015 | Nat Genet. | EGR2, ADO, LOC107984012 | 10 | rs6479858 | 6,1940E-03 | intergene |
| Grünewald TG., 2015 | Nat Genet. | LOC107984012, NRBF2 | 10 | rs6479860 | 2,2830E-06 | intergene |
| Postel-Vinay S., 2012 | Nat Genet. | LOC107984012, NRBF2 | 10 | rs6479860 | 1,70E-12 | intergene |
| Mirabello L., 2011 | BMC Cancer | SRD5A2 | 2 | rs6543631 | 0,0344 | intergene |
| Mirabello L., 2011 | BMC Cancer | FGFR3 | 4 | rs6599400 | 0,0069 | intergene |
| Naumov VA., 2012 | Bull Exp Biol Med. | FGFR3 | 4 | rs6599400 | 0,0585 | intergene |
| Mirabello L., 2011 | BMC Cancer | SRD5A2 | 2 | rs6732223 | 0,0372 | intergene |
| Savage SA., 2013 | Nat Genet. | LOC101927822 | 8 | rs6982411 | 0,0107 | intergene |
| Savage SA., 2013 | Nat Genet. | LOC105375771 | 8 | rs6986484 | 0,0303 | intergene |
| Savage SA., 2013 | Nat Genet. | LY6L | 8 | rs6988480 | 0,0456 | intergene |
| Savage SA., 2013 | Nat Genet. | LOC101927845 | 8 | rs6992707 | 0,0119 | intergene |
| Savage SA., 2013 | Nat Genet. | LOC105375792, TSNARE1 | 8 | rs6993344 | 0,0268 | intergene |
| Savage SA., 2013 | Nat Genet. | GSDMC | 8 | rs6995598 | 0,0442 | intergene |
| Savage SA., 2013 | Nat Genet. | LOC105375776, LOC401478 | 8 | rs6996902 | 0,0058 | intergene |
| Savage SA., 2013 | Nat Genet. | LOC101927822 | 8 | rs6999388 | 0,008 | intergene |
| Savage SA., 2013 | Nat Genet. | ZFAT | 8 | rs7012883 | 0,0427 | intergene |
| Mirabello L., 2011 | BMC Cancer | RPS6, ACER2 |  | rs7048650 | 0,0478 | intergene |
| Grünewald TG., 2015 | Nat Genet. | EGR2, ADO, LOC107984012 | 10 | rs7068658 | 5,0860E-03 | intergene |
| Grünewald TG., 2015 | Nat Genet. | EGR2, LOC107984012 | 10 | rs7073383 | 5,9760E-06 | intergene |
| Grünewald TG., 2015 | Nat Genet. | EGR2, ADO, LOC107984012 | 10 | rs7073414 | 2,3590E-04 | intergene |
| Grünewald TG., 2015 | Nat Genet. | ZNF365, ADO, EGR2 | 10 | rs7073430 | 4,5630E-05 | intergene |
| Grünewald TG., 2015 | Nat Genet. | EGR2, ADO, LOC107984012 | 10 | rs7076055 | 2,4140E-05 | intergene |
| Grünewald TG., 2015 | Nat Genet. | EGR2, ADO, LOC107984012 | 10 | rs7076256 | 6,4110E-04 | intergene |
| Grünewald TG., 2015 | Nat Genet. | EGR2, ADO, LOC107984012 | 10 | rs7076363 | 1,4660E-03 | intergene |
| Grünewald TG., 2015 | Nat Genet. | EGR2, ADO, LOC107984012 | 10 | rs7076790 | 6,4700E-03 | intergene |
| Grünewald TG., 2015 | Nat Genet. | EGR2, ADO, LOC107984012 | 10 | rs7078554 | 1,6830E-03 | intergene |
| Grünewald TG., 2015 | Nat Genet. | EGR2, LOC107984012 | 10 | rs7079482 | 2,6870E-06 | intergene |
| Grünewald TG., 2015 | Nat Genet. | EGR2, ADO, LOC107984012 | 10 | rs7080482 | 3,1340E-03 | intergene |
| Grünewald TG., 2015 | Nat Genet. | EGR2, ADO, LOC107984012 | 10 | rs7082943 | 2,0650E-04 | intergene |
| Grünewald TG., 2015 | Nat Genet. | EGR2, ADO, LOC107984012 | 10 | rs7084016 | 3,0630E-05 | intergene |
| Grünewald TG., 2015 | Nat Genet. | ZNF365, ADO, EGR2 | 10 | rs7088066 | 7,9820E-04 | intergene |
| Grünewald TG., 2015 | Nat Genet. | ZNF365, ADO, EGR2 | 10 | rs7088592 | 3,6380E-05 | intergene |
| Grünewald TG., 2015 | Nat Genet. | EGR2, ADO, LOC107984012 | 10 | rs7088601 | 7,9820E-04 | intergene |
| Grünewald TG., 2015 | Nat Genet. | EGR2, ADO, LOC107984012 | 10 | rs7088733 | 5,6860E-03 | intergene |
| Grünewald TG., 2015 | Nat Genet. | EGR2, ADO, LOC107984012 | 10 | rs7089121 | 7,9820E-04 | intergene |
| Grünewald TG., 2015 | Nat Genet. | EGR2, ADO, LOC107984012 | 10 | rs7093716 | 9,9440E-04 | intergene |
| Mirabello L., 2011 | BMC Cancer | GDF10, GDF2 | 10 | rs7093975 | 0,0328 | intergene |
| Grünewald TG., 2015 | Nat Genet. | EGR2, ADO, LOC107984012 | 10 | rs7096063 | 4,4080E-03 | intergene |
| Grünewald TG., 2015 | Nat Genet. | EGR2, ADO | 10 | rs7096645 | 1,5390E-06 | intergene |
| Grünewald TG., 2015 | Nat Genet. | EGR2, ADO, LOC107984012 | 10 | rs7097383 | 7,9820E-04 | intergene |
| Grünewald TG., 2015 | Nat Genet. | EGR2, ADO, LOC107984012 | 10 | rs7100204 | 1,0220E-05 | intergene |
| Grünewald TG., 2015 | Nat Genet. | EGR2, ADO, LOC107984012 | 10 | rs7100213 | 3,4370E-06 | intergene |
| Grünewald TG., 2015 | Nat Genet. | EGR2, ADO, LOC107984012 | 10 | rs7100320 | 1,4980E-05 | intergene |
| Savage SA., 2013 | Nat Genet. | LOC105375791 | 8 | rs7386137 | 0,0479 | intergene |
| Mirabello L., 2010 | Carcinogenesis | LOC105375754 | 8 | rs7386167 | 0,0327 | intergene |
| Savage SA., 2013 | Nat Genet. | TSNARE1 | 8 | rs7387733 | 0,0484 | intergene |
| Savage SA., 2013 | Nat Genet. | LOC101927845 | 8 | rs7462529 | 0,0336 | intergene |
| Savage SA., 2013 | Nat Genet. | LOC101927845 | 8 | rs7462577 | 0,0051 | intergene |
| Savage SA., 2013 | Nat Genet. | TSNARE1 | 8 | rs7463711 | 0,0472 | intergene |
| Grünewald TG., 2015 | Nat Genet. | EGR2, ADO, LOC107984012 | 10 | rs7477765 | 4,3660E-04 | intergene |
| Grünewald TG., 2015 | Nat Genet. | EGR2, ADO, LOC107984012 | 10 | rs752602 | 1,5060E-04 | intergene |
| Jiang C., 2014 | Med Oncol. | LOC105373401 | 2 | rs7591996 | 0,6084 | intergene |
| Savage SA., 2013 | Nat Genet. | LOC105373401 | 2 | rs7591996 | 1,04E-08 | intergene |
| Grünewald TG., 2015 | Nat Genet. | ZNF365 | 10 | rs77029323 | 3,0560E-04 | intergene |
| Savage SA., 2013 | Nat Genet. | LOC105375754 | 8 | rs7814563 | 0,0313 | intergene |
| Savage SA., 2013 | Nat Genet. | COL22A1 | 8 | rs7817046 | 0,0018 | intergene |
| Savage SA., 2013 | Nat Genet. | KHDRBS3 | 8 | rs7818542 | 0,0146 | intergene |
| Savage SA., 2013 | Nat Genet. | SLC45A4, GPR20 | 8 | rs7818687 | 0,0268 | intergene |
| Savage SA., 2013 | Nat Genet. | LOC105375776 | 8 | rs7822502 | 0,0197 | intergene |
| Savage SA., 2013 | Nat Genet. | NSMCE2, TRIB1 | 8 | rs7822730 | 0,0199 | intergene |
| Savage SA., 2013 | Nat Genet. | CCDC26 | 8 | rs7823113 | 0,0347 | intergene |
| Savage SA., 2013 | Nat Genet. | LOC105375754 | 8 | rs7824074 | 0,0256 | intergene |
| Savage SA., 2013 | Nat Genet. | SNTB1 | 8 | rs7824584 | 0,0327 | intergene |
| Savage SA., 2013 | Nat Genet. | LOC107986980, LOC107986905 | 8 | rs7827430 | 0,0481 | intergene |
| Savage SA., 2013 | Nat Genet. | SNTB1 | 8 | rs7828438 | 0,0186 | intergene |
| Savage SA., 2013 | Nat Genet. | ST3GAL1, LOC105375773 | 8 | rs7830798 | 0,0277 | intergene |
| Savage SA., 2013 | Nat Genet. | ZFAT, LOC102723694 | 8 | rs7832498 | 0,0291 | intergene |
| Savage SA., 2013 | Nat Genet. | MROH5 | 8 | rs7835431 | 0,0431 | intergene |
| Savage SA., 2013 | Nat Genet. | ZFAT, LOC102723694 | 8 | rs7842889 | 0,0351 | intergene |
| Savage SA., 2013 | Nat Genet. | ZFAT, LOC102723694 | 8 | rs7844529 | 0,0011 | intergene |
| Savage SA., 2013 | Nat Genet. | MROH5 | 8 | rs7845615 | 0,0337 | intergene |
| Grünewald TG., 2015 | Nat Genet. | EGR2, ADO, LOC107984012 | 10 | rs7893375 | 8,8650E-05 | intergene |
| Grünewald TG., 2015 | Nat Genet. | EGR2, ADO, LOC107984012 | 10 | rs7895364 | 9,7440E-05 | intergene |
| Grünewald TG., 2015 | Nat Genet. | ZNF365, ADO, EGR2 | 10 | rs7897856 | 2,8800E-04 | intergene |
| Grünewald TG., 2015 | Nat Genet. | ZNF365, ADO, EGR2 | 10 | rs7905229 | 1,0890E-05 | intergene |
| Grünewald TG., 2015 | Nat Genet. | LOC107984012 | 10 | rs7905789 | 1,0370E-05 | intergene |
| Grünewald TG., 2015 | Nat Genet. | LOC107984012 | 10 | rs7909584 | 9,9770E-03 | intergene |
| Grünewald TG., 2015 | Nat Genet. | EGR2, ADO, LOC107984012 | 10 | rs7912385 | 2,8800E-04 | intergene |
| Grünewald TG., 2015 | Nat Genet. | EGR2, ADO, LOC107984012 | 10 | rs7912700 | 2,8800E-04 | intergene |
| Grünewald TG., 2015 | Nat Genet. | EGR2, ADO, LOC107984012 | 10 | rs7913336 | 6,2120E-04 | intergene |
| Grünewald TG., 2015 | Nat Genet. | LOC107984012 | 10 | rs7923409 | 4,5540E-03 | intergene |
| Postel-Vinay S., 2012 | Nat Genet. | LOC107984012 | 10 | rs79965208 |  | intergene |
| Savage SA., 2013 | Nat Genet. | LOC105370773, TMCO5A | 15 | rs8036460 | 5,83E-05 | intergene |
| Mirabello L., 2011 | BMC Cancer | HSD17B2, SDR42E1 | 16 | rs8058561 | 0,0218 | intergene |
| Mirabello L., 2011 | BMC Cancer | TMEM169, XRCC5 | 2 | rs828918 | 0,0072 | intergene |
| Savage SA., 2013 | Nat Genet. | ASAP1 | 8 | rs868104 | 0,0191 | intergene |
| Savage SA., 2013 | Nat Genet. | LOC105375791 | 8 | rs873225 | 0,0497 | intergene |
| Mirabello L., 2011 | BMC Cancer | CTGF | 6 | rs928501 | 0,0048 | intergene |
| Savage SA., 2013 | Nat Genet. | MAL2 | 8 | rs9297594 | 2,24E-04 | intergene |
| Savage SA., 2013 | Nat Genet. | LOC105375765, LRRC6 | 8 | rs9297852 | 0,0104 | intergene |
| Mirabello L., 2011 | BMC Cancer | DDR1, GTF2H4 | 6 | rs9378150 | 0,0388 | intergene |
| Mirabello L., 2011 | BMC Cancer | GDF10, GDF2 | 10 | rs9421731 | 0,0157 | intergene |
| Postel-Vinay S., 2012 | Nat Genet. | C1orf127, TARDBP | 1 | rs9430161 | 1,40E-20 | intergene |
| Mirabello L., 2011 | BMC Cancer | MSH4, ASB17 | 1 | rs946163 | 0,0291 | intergene |
| Grünewald TG., 2015 | Nat Genet. | EGR2, ADO, LOC107984012 | 10 | rs949566 | 1,7110E-03 | intergene |
| Grünewald TG., 2015 | Nat Genet. | EGR2, LOC107984012 | 10 | rs9633562 | 7,9130E-04 | intergene |
| Postel-Vinay S., 2012 | Nat Genet. | EGR2, LOC107984012 | 10 | rs9633562 | 3,90E-11 | intergene |
| Savage SA., 2013 | Nat Genet. | CCDC26 | 8 | rs9643245 | 0,0132 | intergene |
| Savage SA., 2013 | Nat Genet. | LOC107986905 | 8 | rs9644431 | 0,0487 | intergene |
| Mirabello L., 2011 | BMC Cancer | ERCC4 | 16 | rs9646271 | 0,0306 | intergene |
| Savage SA., 2013 | Nat Genet. | LOC105375794, LOC100133669 | 8 | rs9692931 | 0,018 | intergene |
| Grünewald TG., 2015 | Nat Genet. | EGR2, LOC107984012 | 10 | rs983319 | 2,9860E-06 | intergene |
| Grünewald TG., 2015 | Nat Genet. | EGR2, ADO, LOC107984012 | 10 | rs983320 | 3,0110E-05 | intergene |
| Savage SA., 2007 | Pediat Blood Cancer | TP53 | 17 | rs9894946 | 0,5347 | intergene |
| Savage SA., 2013 | Nat Genet. | KCNK9 | 8 | rs994769 | 0,0341 | intergene |
| Grünewald TG., 2015 | Nat Genet. | EGR2, ADO, LOC107984012 | 10 | rs9988771 | 1,6550E-04 | intergene |
| Grünewald TG., 2015 | Nat Genet. | ADO | 10 | rs12220700 | 9,0680E-05 | intregene |
| Grünewald TG., 2015 | Nat Genet. | EGR2, ADO, LOC107984012 | 10 | rs12258444 | 1,5330E-03 | intregene |
| Savage SA., 2013 | Nat Genet. | LOC105374557 | 4 | rs10025742 | 2,79E-05 | intron |
| Cong Y., 2014 | Tumor Biol. | BMP2 | 20 | rs1005464 | 0,004 | intron |
| Savage SA., 2013 | Nat Genet. | CCAT1 | 8 | rs10087719 | 0,0085 | intron |
| Savage SA., 2013 | Nat Genet. | LOC107986905 | 8 | rs10088280 | 0,0249 | intron |
| Savage SA., 2013 | Nat Genet. | PVT1, LINC00824 | 8 | rs10089519 | 3,87E-04 | intron |
| Savage SA., 2013 | Nat Genet. | LOC401478 | 8 | rs10101858 | 0,0309 | intron |
| Savage SA., 2013 | Nat Genet. | TSNARE1 | 8 | rs10102944 | 0,0495 | intron |
| Savage SA., 2013 | Nat Genet. | ZFAT | 8 | rs10103826 | 0,0098 | intron |
| Savage SA., 2013 | Nat Genet. | KHDRBS3 | 8 | rs10104847 | 0,0328 | intron |
| Mirabello L., 2011 | BMC Cancer | MPG | 16 | rs1013358 | 0,0300 | intron |
| Mirabello L., 2011 | BMC Cancer | UGT1A8 | 2 | rs10179091 | 0,0276 | intron |
| Savage SA., 2013 | Nat Genet. | KCNQ3 | 8 | rs1020740 | 0,0491 | intron |
| Savage SA., 2013 | Nat Genet. | ST3GAL1 | 8 | rs1040339 | 0,0199 | intron |
| Savage SA., 2013 | Nat Genet. | LOC105375716 | 8 | rs10505309 | 0,0438 | intron |
| Savage SA., 2013 | Nat Genet. | ENPP2 | 8 | rs10505371 | 0,0263 | intron |
| Mirabello L., 2011 | BMC Cancer | CDKN2B | 9 | rs10738604 | 0,0366 | intron |
| Savage SA., 2013 | Nat Genet. | TGFBR1 | 9 | rs10739778 | 0,0339 | intron |
| Grünewald TG., 2015 | Nat Genet. | LOC107984012 | 10 | rs10740094 | 6,2750E-03 | intron |
| Grünewald TG., 2015 | Nat Genet. | LOC107984012 | 10 | rs10740095 | 5,4960E-06 | intron |
| Mirabello L., 2011 | BMC Cancer | MGMT | 10 | rs10741191 | 0,0156 | intron |
| Zhang N., 2016 | Onco Targets Ther. | WWOX | 16 | rs1074963 | 0,9933 | intron |
| Grünewald TG., 2015 | Nat Genet. | LOC107984012 | 10 | rs10761678 | 9,2930E-03 | intron |
| Grünewald TG., 2015 | Nat Genet. | LOC107984012 | 10 | rs10761679 | 9,5580E-03 | intron |
| Grünewald TG., 2015 | Nat Genet. | LOC107984012 | 10 | rs10761680 | 9,5580E-03 | intron |
| Savage SA., 2013 | Nat Genet. | KCNMA1 | 10 | rs10762759 | 1,23E-04 | intron |
| Savage SA., 2013 | Nat Genet. | LINC00977, CCDC26 | 8 | rs10808577 | 0,0059 | intron |
| Grünewald TG., 2015 | Nat Genet. | LOC107984012 | 10 | rs10822085 | 5,9490E-03 | intron |
| Mirabello L., 2011 | BMC Cancer | MGMT | 10 | rs10829619 | 0,0023 | intron |
| Mirabello L., 2011 | BMC Cancer | IGF1 | 12 | rs10860864 | 0,0039 | intron |
| Mirabello L., 2011 | BMC Cancer | IGF1 | 12 | rs10860869 | 0,0184 | intron |
| Savage SA., 2013 | Nat Genet. | TRAPPC9 | 8 | rs10875447 | 0,0273 | intron |
| Mirabello L., 2011 | BMC Cancer | POLL | 10 | rs10883663 | 0,0179 | intron |
| Savage SA., 2013 | Nat Genet. | ASAP1 | 8 | rs10956511 | 0,0187 | intron |
| Savage SA., 2013 | Nat Genet. | LOC105375760 | 8 | rs10956576 | 0,0081 | intron |
| Mirabello L., 2011 | BMC Cancer | APTX | 9 | rs10971259 | 0,0076 | intron |
| Mirabello L., 2011 | BMC Cancer | APTX | 9 | rs10971263 | 0,0174 | intron |
| Mirabello L., 2011 | BMC Cancer | FANCG | 9 | rs10972310 | 0,0406 | intron |
| Mirabello L., 2011 | BMC Cancer | RAD23B | 9 | rs10978792 | 0,0369 | intron |
| Mirabello L., 2011 | BMC Cancer | RPS24 | 10 | rs11002385 | 0,0260 | intron |
| Mirabello L., 2011 | BMC Cancer | MGMT | 10 | rs11016811 | 0,0440 | intron |
| Walsh KM., 2016 | Carcinogenesis. | ACYP2 | 2 | rs11125529 | 0,91 | intron |
| Mirabello L., 2011 | BMC Cancer | HSD17B2 | 16 | rs11150436 | 0,0347 | intron |
| Mirabello L., 2011 | BMC Cancer | FANCM | 14 | rs11157432 | 0,0405 | intron |
| Grochola LF., 2009 | Clin. Cancer Res. | PPP2R5E | 14 | rs11158941 | 0,1209 | intron |
| Mirabello L., 2011 | BMC Cancer | MSH4 | 1 | rs11161848 | 0,0235 | intron |
| Mirabello L., 2011 | BMC Cancer | MSH4 | 1 | rs11161887 | 0,0323 | intron |
| Savage SA., 2013 | Nat Genet. | FAM13 | 8 | rs11166797 | 0,0067 | intron |
| Savage SA., 2013 | Nat Genet. | TRAPPC9 | 8 | rs11166964 | 0,0382 | intron |
| Mirabello L., 2011 | BMC Cancer | POLL | 10 | rs11191064 | 0,0255 | intron |
| Mirabello L., 2011 | BMC Cancer | AKR1C3 | 10 | rs11252932 | 0,0237 | intron |
| Mirabello L., 2011 | BMC Cancer | GC | 4 | rs1155563 | 0,0353 | intron |
| Mirabello L., 2011 | BMC Cancer | RAD23B | 9 | rs11573709 | 0,0097 | intron |
| Postel-Vinay S., 2012 | Nat Genet. | C1orf127 | 1 | rs11576658 | 9,40E-11 | intron |
| Mirabello L., 2011 | BMC Cancer | MGMT | 10 | rs11592922 | 0,0028 | intron |
| Savage SA., 2013 | Nat Genet. | KCNK9 | 8 | rs1159873 | 0,0037 | intron |
| Savage SA., 2013 | Nat Genet. | WWOX | 16 | rs11648121 | 1,67E-02 | intron |
| Mirabello L., 2011 | BMC Cancer | HSD17B2 | 16 | rs11648233 | 0,0331 | intron |
| Mirabello L., 2011 | BMC Cancer | HIP1 | 7 | rs1167795 | 0,0319 | intron |
| DuBois SG., 2012 | Pediatr Blood Cancer | EWSR1 | 22 | rs11703595 | 0,29 | intron |
| Mirabello L., 2011 | BMC Cancer | NUDT6 | 4 | rs11737764 | 0,0016 | intron |
| Naumov VA., 2012 | Bull Exp Biol Med. | NUDT6 | 4 | rs11737764 | 0,5962 | intron |
| Savage SA., 2013 | Nat Genet. | LOC105375751 | 8 | rs11784545 | 0,0116 | intron |
| Mirabello L., 2011 | BMC Cancer | FANCM | 14 | rs11845507 | 0,0033 | intron |
| DuBois SG., 2012 | Pediatr Blood Cancer | EWSR1 | 22 | rs11913870 | 0,88 | intron |
| Mirabello L., 2011 | BMC Cancer | MDM2 | 12 | rs1196334 | 0,0081 | intron |
| Savage SA., 2013 | Nat Genet. | TRAPPC9 | 8 | rs11991065 | 0,0049 | intron |
| Savage SA., 2013 | Nat Genet. | TRAPPC9 | 8 | rs11992718 | 0,0146 | intron |
| Savage SA., 2013 | Nat Genet. | FER1L6 | 8 | rs11993947 | 0,0385 | intron |
| Savage SA., 2013 | Nat Genet. | LOC107986976 | 8 | rs11997238 | 0,037 | intron |
| Mirabello L., 2011 | BMC Cancer | BMP6 | 6 | rs12210175 | 0,0432 | intron |
| Mirabello L., 2011 | BMC Cancer | FRS2 | 12 | rs12371904 | 0,0244 | intron |
| Mirabello L., 2011 | BMC Cancer | MBD4 | 3 | rs12485319 | 0,0484 | intron |
| Savage SA., 2013 | Nat Genet. | GPR149 | 3 | rs12488867 | 3,78E-04 | intron |
| Savage SA., 2013 | Nat Genet. | CREB5 | 7 | rs12531253 | 2,11E-04 | intron |
| Savage SA., 2013 | Nat Genet. | ARHGEF10 | 8 | rs12545104 | 1,86E-05 | intron |
| Savage SA., 2013 | Nat Genet. | TSNARE1 | 8 | rs12547214 | 0,0392 | intron |
| Savage SA., 2013 | Nat Genet. | TRAPPC9 | 8 | rs12548239 | 0,0415 | intron |
| Mirabello L., 2011 | BMC Cancer | HSD17B3 | 9 | rs12552648 | 0,0399 | intron |
| Mirabello L., 2011 | BMC Cancer | ESR2 | 14 | rs1256064 | 0,0399 | intron |
| Ru JY., 2015 | Int J Clin Exp Pathol. | TP53 | 17 | rs12602273 | 0,1449 | intron |
| Mirabello L., 2011 | BMC Cancer | LHCGR | 2 | rs12618729 | 0,0396 | intron |
| Mirabello L., 2011 | BMC Cancer | DDR1 | 6 | rs1264320 | 0,0340 | intron |
| Savage SA., 2013 | Nat Genet. | ZHX2 | 8 | rs12675789 | 0,003 | intron |
| Savage SA., 2013 | Nat Genet. | VDR, LOC107984503 | 12 | rs12721364 | 0,0039 | intron |
| Mirabello L., 2011 | BMC Cancer | PARP4 | 13 | rs12863638 | 0,0072 | intron |
| Savage SA., 2013 | Nat Genet. | COL22A1 | 8 | rs1320270 | 0,0257 | intron |
| Savage SA., 2013 | Nat Genet. | COLEC10 | 8 | rs13254750 | 0,0282 | intron |
| Savage SA., 2013 | Nat Genet. | TRAPPC9 | 8 | rs13259162 | 0,022 | intron |
| Savage SA., 2013 | Nat Genet. | ZHX1, C8orf76 | 8 | rs13261992 | 0,0157 | intron |
| Savage SA., 2013 | Nat Genet. | CCDC26 | 8 | rs13267039 | 0,0256 | intron |
| Savage SA., 2013 | Nat Genet. | LOC107986971 | 8 | rs13267240 | 0,0201 | intron |
| Savage SA., 2013 | Nat Genet. | KCNQ3 | 8 | rs13267466 | 0,0022 | intron |
| Savage SA., 2013 | Nat Genet. | CCDC26 | 8 | rs13269801 | 0,0307 | intron |
| Savage SA., 2013 | Nat Genet. | LOC105375759 | 8 | rs13270165 | 0,0406 | intron |
| Savage SA., 2013 | Nat Genet. | AGO2 | 8 | rs13276958 | 0,0322 | intron |
| Savage SA., 2013 | Nat Genet. | TRAPPC9 | 8 | rs13282606 | 0,0362 | intron |
| Mirabello L., 2011 | BMC Cancer | HSD17B2 | 16 | rs13337293 | 0,0379 | intron |
| DuBois SG., 2012 | Pediatr Blood Cancer | EWSR1 | 22 | rs140062 | 0,4388 | intron |
| DuBois SG., 2012 | Pediatr Blood Cancer | EWSR1 | 22 | rs140065 | 0,9247 | intron |
| Zhang N., 2016 | Onco Targets Ther. | WWOX | 16 | rs1424110 | 0,242 | intron |
| Savage SA., 2013 | Nat Genet. | KCNQ3 | 8 | rs1457785 | 0,034 | intron |
| Savage SA., 2013 | Nat Genet. | THSD7A | 7 | rs1467345 | 4,64E-03 | intron |
| Savage SA., 2013 | Nat Genet. | LOC105375751 | 8 | rs1487232 | 0,0181 | intron |
| Savage SA., 2013 | Nat Genet. | SLC30A8 | 8 | rs1505521 | 0,0383 | intron |
| Mirabello L., 2011 | BMC Cancer | IGF1R | 15 | rs1546713 | 0,0437 | intron |
| Savage SA., 2013 | Nat Genet. | LINC00861 | 8 | rs1602511 | 0,0298 | intron |
| Savage SA., 2007 | Pediat Blood Cancer | TP53 | 17 | rs1625895 | 0,8459 | intron |
| Martinelli M., 2016 | Oncotarget | CD99 | Y | rs167453 | 0,7384 | intron |
| Mirabello L., 2011 | BMC Cancer | XRCC5 | 2 | rs16855489 | 0,0221 | intron |
| Mirabello L., 2011 | BMC Cancer | XRCC5 | 2 | rs16855552 | 0,0016 | intron |
| Savage SA., 2013 | Nat Genet. | LOC105375725 | 8 | rs16892216 | 0,0485 | intron |
| Savage SA., 2013 | Nat Genet. | ZHX2 | 8 | rs16897499 | 0,0326 | intron |
| Savage SA., 2013 | Nat Genet. | CCDC26 | 8 | rs16904064 | 0,0291 | intron |
| Savage SA., 2013 | Nat Genet. | GSDMC | 8 | rs16904150 | 0,013 | intron |
| Savage SA., 2013 | Nat Genet. | ASAP1 | 8 | rs16904239 | 0,0418 | intron |
| Savage SA., 2013 | Nat Genet. | ST3GAL1 | 8 | rs16904945 | 0,024 | intron |
| Yang W., 2014 | Med Oncol. | ITGA3 | 17 | rs16948627 | 0,2824 | intron |
| Miao C., 2015 | Sci. Rep. | MDM2 | 12 | rs1695147 | 0,0213 | intron |
| Mirabello L., 2011 | BMC Cancer | MDM2 | 12 | rs1695147 | 0,0103 | intron |
| Mirabello L., 2011 | BMC Cancer | HSD17B2 | 2 | rs16956274 | 0,0217 | intron |
| Mirabello L., 2011 | BMC Cancer | HSD17B2 | 2 | rs16956406 | 0,0113 | intron |
| Mirabello L., 2011 | BMC Cancer | CEP89 | 19 | rs16967668 | 0,0321 | intron |
| Mirabello L., 2011 | BMC Cancer | MAPK8IP3 | 16 | rs17135510 | 0,0438 | intron |
| Mirabello L., 2011 | BMC Cancer | COL1A2 | 7 | rs17166206 | 0,0178 | intron |
| Savage SA., 2013 | Nat Genet. | COLEC10 | 8 | rs17179583 | 0,0158 | intron |
| Jiang C., 2014 | Med Oncol. | ADAMTS6 | 5 | rs17206779 | 0,6392 | intron |
| Savage SA., 2013 | Nat Genet. | ADAMTS6 | 6 | rs17206779 | 5,07E-07 | intron |
| Savage SA., 2013 | Nat Genet. | FER1L6 | 8 | rs17270769 | 0,0193 | intron |
| Mirabello L., 2011 | BMC Cancer | UMAD1, RPA3 | 7 | rs17482927 | 0,0277 | intron |
| Mirabello L., 2011 | BMC Cancer | COL1A1 | 17 | rs17639446 | 0,0422 | intron |
| Mirabello L., 2011 | BMC Cancer | IGF1 | 12 | rs17796225 | 0,0040 | intron |
| Martinelli M., 2016 | Oncotarget | CD99 | Y | rs178127 | 0,5534 | intron |
| Savage SA., 2013 | Nat Genet. | COL14A1 | 8 | rs17833457 | 0,0476 | intron |
| Mirabello L., 2011 | BMC Cancer | UGT1A8 | 2 | rs17862866 | 0,0377 | intron |
| Savage SA., 2007 | Pediat Blood Cancer | TP53 | 17 | rs17880604 | 0,1181 | intron |
| Savage SA., 2007 | Pediat Blood Cancer | TP53 | 17 | rs17887200 | 0,7729 | intron |
| Wu Y., 2015 | Tumor Biol. | TGFB1 | 19 | rs1800473 | 0,0107 | intron |
| Savage SA., 2013 | Nat Genet. | TG | 8 | rs180204 | 0,0223 | intron |
| Mirabello L., 2011 | BMC Cancer | PARP4 | 13 | rs1807271 | 0,0221 | intron |
| Savage SA., 2013 | Nat Genet. | EEF1D | 8 | rs1809148 | 0,0384 | intron |
| Xin DJ., 2015 | Int J Clin Exp Pathol. | HER2 | 17 | rs1810132 | 0,0895 | intron |
| Mirabello L., 2011 | BMC Cancer | MDM2 | 12 | rs1846402 | 0,0226 | intron |
| Mirabello L., 2011 | BMC Cancer | RPS24 | 10 | rs1863898 | 0,0371 | intron |
| Savage SA., 2013 | Nat Genet. | SLC45A4 | 8 | rs1865246 | 0,0046 | intron |
| Jiang C., 2014 | Med Oncol. | GRM4 | 6 | rs1906953 | 0,0153 | intron |
| Savage SA., 2013 | Nat Genet. | GRM4 | 6 | rs1906953 | 8,05E-09 | intron |
| Wang K., 2016 | Tumour Biol. | GRM4 | 6 | rs1906953 | 0,0099 | intron |
| Savage SA., 2013 | Nat Genet. | LOC401478 | 8 | rs1949141 | 0,0498 | intron |
| Savage SA., 2013 | Nat Genet. | CREB5 | 7 | rs1964240 | 3,12E-03 | intron |
| Postel-Vinay S., 2012 | Nat Genet. | C1orf127 | 1 | rs2003046 | 1,30E-14 | intron |
| Mirabello L., 2011 | BMC Cancer | MGMT | 10 | rs2026975 | 0,0222 | intron |
| Savage SA., 2013 | Nat Genet. | PVT1 | 8 | rs2033098 | 0,0363 | intron |
| Savage SA., 2013 | Nat Genet. | FAM49B | 8 | rs2060983 | 0,0237 | intron |
| Miao C., 2015 | Sci. Rep. | CDK4 | 12 | rs2069502 | 0,5101 | intron |
| He M., 2013 | Tumor Biol. | COL1A1 | 17 | rs2075559 | 0,0089 | intron |
| Mirabello L., 2011 | BMC Cancer | XRCC5 | 2 | rs207876 | 0,0194 | intron |
| Savage SA., 2013 | Nat Genet. | ADAMTS17 | 15 | rs2086452 | 1,12E-06 | intron |
| Savage SA., 2013 | Nat Genet. | ESR1,FAS/ER | 6 | rs2144025 | 0,0163 | intron |
| Savage SA., 2013 | Nat Genet. | FAM91A1 | 8 | rs2163534 | 0,01 | intron |
| Mirabello L., 2011 | BMC Cancer | MPG |  | rs216614 | 0,0036 | intron |
| Savage SA., 2013 | Nat Genet. | AGO2 | 8 | rs2176397 | 0,0224 | intron |
| Chen Y., 2016 | Tumour Biol. | CXCL8 | 4 | rs2227306 | 0,6357 | intron |
| Savage SA., 2013 | Nat Genet. | TRAPPC9 | 8 | rs2233230 | 0,0014 | intron |
| Ruza E., 2003 | J Pediatr Hematol Oncol | ER | 6 | rs2234693 | 0,6034 | intron |
| Xu H., 2016 | Med Sci Monit. | RASSF1A | 3 | rs2236947 | 0,1181 | intron |
| Mirabello L., 2011 | BMC Cancer | XRCC5 | 2 | rs2241320 | 0,0018 | intron |
| Lu H., 2015 | Tumor Biol. | PRKCG | 19 | rs2242245 | 0,8335 | intron |
| Zhang Y., 2014 | Tumor Biol. | PRKCG | 19 | rs2242245 | 0,3106 | intron |
| Savage SA., 2013 | Nat Genet. | RAD50 | 5 | rs2244012 | 8,94E-04 | intron |
| Mirabello L., 2011 | BMC Cancer | FGFR2 | 10 | rs2247088 | 0,0318 | intron |
| Savage SA., 2013 | Nat Genet. | TG, SLA, SLAP | 8 | rs2252553 | 0,0444 | intron |
| Savage SA., 2013 | Nat Genet. | EXT1 | 8 | rs2255931 | 0,0092 | intron |
| Savage SA., 2013 | Nat Genet. | ZFP41 | 8 | rs2272631 | 0,0267 | intron |
| Savage SA., 2013 | Nat Genet. | DENND3 | 8 | rs2278448 | 0,0441 | intron |
| Savage SA., 2007 | Cancer Epidemiol Biomarkers Prev. | IGFR2 | 6 | rs2282140 | 0,4705 | intron |
| Mirabello L., 2011 | BMC Cancer | IGF2R | 6 | rs2282141 | 0,0082 | intron |
| Mirabello L., 2011 | BMC Cancer | ATM | 11 | rs228606 | 0,0290 | intron |
| Mirabello L., 2011 | BMC Cancer | RPA1 | 17 | rs2287320 | 0,0322 | intron |
| Mirabello L., 2011 | BMC Cancer | IGF1 | 12 | rs2288378 | 0,0439 | intron |
| Mirabello L., 2011 | BMC Cancer | IGF2R | 6 | rs2297372 | 0,0237 | intron |
| Mirabello L., 2011 | BMC Cancer | PNKP | 19 | rs2305922 | 0,0245 | intron |
| Savage SA., 2013 | Nat Genet. | LOC401478 | 8 | rs2326130 | 0,0139 | intron |
| Cong Y., 2014 | Tumor Biol. | BMP2 | 20 | rs235764 | 0,6637 | intron |
| Savage SA., 2013 | Nat Genet. | EXT1 | 8 | rs2451157 | 0,008 | intron |
| Savage SA., 2013 | Nat Genet. | HHLA1 | 8 | rs2469608 | 0,035 | intron |
| Mirabello L., 2011 | BMC Cancer | CYP19A1 | 15 | rs2470150 | 0,0384 | intron |
| Savage SA., 2013 | Nat Genet. | SAMD12 | 8 | rs2514734 | 0,0274 | intron |
| DuBois SG., 2012 | Pediatr Blood Cancer | EWSR1 | 22 | rs2518683 | 0,1317 | intron |
| Mirabello L., 2011 | BMC Cancer | BLM | 15 | rs2518968 | 0,0419 | intron |
| Mirabello L., 2011 | BMC Cancer | COL1A2 | 7 | rs2521205 | 0,0084 | intron |
| Mirabello L., 2011 | BMC Cancer | BLM | 15 | rs2532105 | 0,0374 | intron |
| Savage SA., 2013 | Nat Genet. | KCNK9 | 8 | rs2545457 | 0,0439 | intron |
| Savage SA., 2013 | Nat Genet. | LOC107986980 | 8 | rs2582475 | 0,0452 | intron |
| Savage SA., 2013 | Nat Genet. | KCNQ3 | 8 | rs2597356 | 0,0374 | intron |
| Savage SA., 2013 | Nat Genet. | LOC107986980 | 8 | rs2610104 | 0,046 | intron |
| Savage SA., 2013 | Nat Genet. | MROH5 | 8 | rs2613636 | 0,0444 | intron |
| Savage SA., 2013 | Nat Genet. | TRAPPC9 | 8 | rs2614754 | 0,036 | intron |
| Savage SA., 2013 | Nat Genet. | PVT1 | 8 | rs2648902 | 0,0198 | intron |
| Savage SA., 2013 | Nat Genet. | LOC107986980 | 8 | rs2660666 | 0,0372 | intron |
| Savage SA., 2013 | Nat Genet. | TRAPPC9 | 8 | rs2665917 | 7,98E-04 | intron |
| Savage SA., 2013 | Nat Genet. | TRAPPC9 | 8 | rs2665919 | 0,0437 | intron |
| Savage SA., 2013 | Nat Genet. | TRAPPC9 | 8 | rs2665926 | 0,0094 | intron |
| Savage SA., 2013 | Nat Genet. | TRAPPC9 | 8 | rs2665931 | 0,0295 | intron |
| Savage SA., 2013 | Nat Genet. | TRAPPC9 | 8 | rs2665945 | 0,006 | intron |
| Savage SA., 2013 | Nat Genet. | TRAPPC9 | 8 | rs2665946 | 0,0166 | intron |
| Mirabello L., 2011 | BMC Cancer | BMP6 | 6 | rs267174 | 0,0452 | intron |
| Savage SA., 2013 | Nat Genet. | KCNQ3 | 8 | rs2673557 | 0,0169 | intron |
| Savage SA., 2013 | Nat Genet. | KCNQ3 | 8 | rs2673562 | 0,0231 | intron |
| Savage SA., 2013 | Nat Genet. | KCNQ3 | 8 | rs2673567 | 0,0053 | intron |
| Mirabello L., 2011 | BMC Cancer | GHRL | 3 | rs26802 | 0,0450 | intron |
| Mirabello L., 2011 | BMC Cancer | IGF1R | 15 | rs2684777 | 0,0141 | intron |
| Mirabello L., 2011 | BMC Cancer | IGF1R | 15 | rs2684806 | 0,0128 | intron |
| Savage SA., 2013 | Nat Genet. | GPR149 | 3 | rs2689326 | 1,89E-03 | intron |
| Savage SA., 2013 | Nat Genet. | TG, SLA, SLAP | 8 | rs2703004 | 0,0467 | intron |
| Mirabello L., 2011 | BMC Cancer | BMP6 | 6 | rs270398 | 0,0443 | intron |
| Mirabello L., 2011 | BMC Cancer | BMP6 | 6 | rs270407 | 0,0202 | intron |
| Savage SA., 2013 | Nat Genet. | PVT1 | 8 | rs2720685 | 0,0057 | intron |
| Walsh KM., 2016 | Carcinogenesis. | TERT | 5 | rs2736100 | 0,1476 | intron |
| Mirabello L., 2011 | BMC Cancer | UGT1A8 | 2 | rs2741042 | 0,0424 | intron |
| Mirabello L., 2011 | BMC Cancer | UGT1A8 | 2 | rs2741048 | 0,0486 | intron |
| Mirabello L., 2011 | BMC Cancer | LIG1 | 19 | rs274875 | 0,0211 | intron |
| DuBois SG., 2012 | Pediatr Blood Cancer | EWSR1 | 22 | rs2857461 | 0,07 | intron |
| Miao C., 2015 | Sci. Rep. | MDM2 | 12 | rs2870820 | 0,0181 | intron |
| Xin DJ., 2015 | Int J Clin Exp Pathol. | HER2 | 17 | rs2952155 | 0,1507 | intron |
| Xin DJ., 2015 | Int J Clin Exp Pathol. | HER2 | 17 | rs2952156 | 0,9726 | intron |
| Savage SA., 2013 | Nat Genet. | AGO2 | 8 | rs2977469 | 0,0418 | intron |
| Savage SA., 2013 | Nat Genet. | LOC107986980 | 8 | rs305262 | 0,0262 | intron |
| Savage SA., 2013 | Nat Genet. | FGF2 | 4 | rs308439 | 0,002 | intron |
| Savage SA., 2013 | Nat Genet. | FGF2 | 4 | rs308443 | 0,0036 | intron |
| Mirabello L., 2011 | BMC Cancer | CCNH | 5 | rs3093816 | 0,0357 | intron |
| Mirabello L., 2011 | BMC Cancer | PARP2 | 14 | rs3093919 | 0,0113 | intron |
| Mirabello L., 2011 | BMC Cancer | PARP2 | 14 | rs3093938 | 0,0039 | intron |
| Martinelli M., 2016 | Oncotarget | CD99 | Y | rs311057 | 0,6905 | intron |
| Martinelli M., 2016 | Oncotarget | CD99 | Y | rs311059 | 0,5072 | intron |
| Martinelli M., 2016 | Oncotarget | CD99 | Y | rs311060 | 0,2953 | intron |
| Martinelli M., 2016 | Oncotarget | CD99 | Y | rs311074 | 0,5343 | intron |
| Martinelli M., 2016 | Oncotarget | CD99 | Y | rs311077 | 0,9901 | intron |
| Martinelli M., 2016 | Oncotarget | CD99 | Y | rs311083 | 0,6048 | intron |
| Martinelli M., 2016 | Oncotarget | CD99 | Y | rs312257 | 0,94 | intron |
| Martinelli M., 2016 | Oncotarget | CD99 | Y | rs312258 | 0,0753 | intron |
| Mirabello L., 2011 | BMC Cancer | ERCC4 | 14 | rs3136189 | 0,0411 | intron |
| Mirabello L., 2011 | BMC Cancer | RAD54B | 8 | rs3136405 | 0,0288 | intron |
| Mirabello L., 2011 | BMC Cancer | XRCC3 | 14 | rs3212092 | 0,0119 | intron |
| Mirabello L., 2011 | BMC Cancer | PARP1 | 1 | rs3219123 | 0,0322 | intron |
| Hu YS., 2011 | Med Oncol. | TGFBR1 | 19 | rs334354 | 0,045 | intron |
| Savage SA., 2013 | Nat Genet. | ZC3H3 | 8 | rs369051 | 1,16E-04 | intron |
| Miao C., 2015 | Sci. Rep. | MDM2 | 12 | rs3730536 | 0,0134 | intron |
| Mirabello L., 2011 | BMC Cancer | LIG1 | 19 | rs3730912 | 0,0295 | intron |
| Mirabello L., 2011 | BMC Cancer | BRCA1 | 17 | rs3737559 | 0,0420 | intron |
| Mirabello L., 2011 | BMC Cancer | FGFR2 | 10 | rs3750815 | 0,0176 | intron |
| Mirabello L., 2011 | BMC Cancer | TDG | 12 | rs3751206 | 0,0477 | intron |
| DuBois SG., 2012 | Pediatr Blood Cancer | EWSR1 | 22 | rs3761426 | 0,4696 | intron |
| Savage SA., 2013 | Nat Genet. | ESR1,FAS/ER | 6 | rs3778099 | 0,0092 | intron |
| Savage SA., 2013 | Nat Genet. | TG | 8 | rs3779951 | 0,046 | intron |
| Savage SA., 2013 | Nat Genet. | KCNK9 | 8 | rs3780040 | 0,0444 | intron |
| Mirabello L., 2011 | BMC Cancer | FKBP3, FANCM | 14 | rs3783702 | 0,0086 | intron |
| Mirabello L., 2011 | BMC Cancer | DUT | 15 | rs3784619 | 0,0346 | intron |
| Mirabello L., 2011 | BMC Cancer | LIG1 | 19 | rs3786763 | 0,0370 | intron |
| Mirabello L., 2011 | BMC Cancer | FGF2 | 4 | rs3789138 | 0,0370 | intron |
| Mirabello L., 2011 | BMC Cancer | LIG1 | 19 | rs380359 | 0,0345 | intron |
| Mirabello L., 2011 | BMC Cancer | ERCC6 | 10 | rs3810944 | 0,0425 | intron |
| Mirabello L., 2011 | BMC Cancer | IGF2R | 6 | rs384167 | 0,0210 | intron |
| Savage SA., 2013 | Nat Genet. | LOC107986980 | 8 | rs3850500 | 0,0422 | intron |
| Mirabello L., 2011 | BMC Cancer | TDG | 12 | rs4135128 | 0,0160 | intron |
| Ma X., 2016 | Genet Mol Res. | ERCC3 | 2 | rs4150441 | 0,3318 | intron |
| Ma X., 2016 | Genet Mol Res. | ERCC3 | 2 | rs4150506 | 0,1749 | intron |
| Savage SA., 2013 | Nat Genet. | C8orfK32, FAM13 | 8 | rs4243888 | 0,0036 | intron |
| Mirabello L., 2011 | BMC Cancer | COL1A2 | 12 | rs42521 | 0,0111 | intron |
| Mirabello L., 2011 | BMC Cancer | COL1A2 | 12 | rs42527 | 0,0041 | intron |
| Mirabello L., 2011 | BMC Cancer | ERCC6 | 10 | rs4253099 | 0,0441 | intron |
| Mirabello L., 2011 | BMC Cancer | ERCC6 | 10 | rs4253200 | 0,0249 | intron |
| Mirabello L., 2011 | BMC Cancer | UGT1A8 | 2 | rs4294999 | 0,0279 | intron |
| Savage SA., 2013 | Nat Genet. | TG, SLA, SLAP | 8 | rs4301434 | 0,0284 | intron |
| Savage SA., 2013 | Nat Genet. | COL22A1 | 8 | rs4301456 | 0,0099 | intron |
| Savage SA., 2013 | Nat Genet. | ZFAT | 8 | rs4424215 | 0,0276 | intron |
| Mirabello L., 2011 | BMC Cancer | ENDOV, FLJ35220 | 17 | rs4424945 | 0,0390 | intron |
| Mirabello L., 2011 | BMC Cancer | ENDOV, FLJ35220 | 17 | rs4491586 | 0,0086 | intron |
| Savage SA., 2013 | Nat Genet. | GSDMC | 8 | rs4517087 | 5,00E-04 | intron |
| Lu H., 2015 | Tumor Biol. | PRKCG | 19 | rs454006 | 0,0077 | intron |
| Zhang Y., 2014 | Tumor Biol. | PRKCG | 19 | rs454006 | 0,0005 | intron |
| Savage SA., 2013 | Nat Genet. | COL22A1 | 8 | rs4549820 | 0,0467 | intron |
| Savage SA., 2013 | Nat Genet. | LOC101927755 | 17 | rs4632189 | 2,55E-05 | intron |
| Mirabello L., 2011 | BMC Cancer | UGT1A8 | 2 | rs4663963 | 0,0356 | intron |
| Mirabello L., 2011 | BMC Cancer | UGT1A8 | 2 | rs4663969 | 0,0465 | intron |
| Mirabello L., 2011 | BMC Cancer | EIF2AK2 | 2 | rs4670185 | 0,0464 | intron |
| Mirabello L., 2011 | BMC Cancer | POLQ | 2 | rs4676727 | 0,0445 | intron |
| Xu H., 2016 | Med Sci Monit. | RASSF1A | 3 | rs4688728 | 0,6236 | intron |
| Savage SA., 2013 | Nat Genet. | COL22A1 | 8 | rs4736293 | 0,0375 | intron |
| Savage SA., 2013 | Nat Genet. | LOC105375759 | 8 | rs4736741 | 0,0077 | intron |
| Grünewald TG., 2015 | Nat Genet. | LOC107984012 | 10 | rs4746725 | 7,1550E-03 | intron |
| Mirabello L., 2011 | BMC Cancer | PARP4 | 13 | rs4770687 | 0,0465 | intron |
| Mirabello L., 2011 | BMC Cancer | CEP89 | 19 | rs4805834 | 0,0402 | intron |
| DuBois SG., 2012 | Pediatr Blood Cancer | EWSR1 | 22 | rs4820803 | 0,6866 | intron |
| Silva DSBS., 2012 | Gene | EWSR1 | 22 | rs4820803 | 0,7384 | intron |
| Silva DSBS., 2012 | Gene | EWSR1 | 22 | rs4820804 | 0,2109 | intron |
| Savage SA., 2013 | Nat Genet. | FBXO3 | 8 | rs4870855 | 0,0339 | intron |
| Savage SA., 2013 | Nat Genet. | SNTB1 | 8 | rs4871072 | 0,0154 | intron |
| Savage SA., 2013 | Nat Genet. | LOC105375734 | 8 | rs4871275 | 0,0453 | intron |
| Savage SA., 2013 | Nat Genet. | ZHX2 | 8 | rs4871320 | 0,0251 | intron |
| Savage SA., 2013 | Nat Genet. | MTSS1 | 8 | rs4871506 | 0,008 | intron |
| Savage SA., 2013 | Nat Genet. | MTSS1 | 8 | rs4871507 | 0,0164 | intron |
| Savage SA., 2013 | Nat Genet. | MTSS1 | 8 | rs4871510 | 0,0221 | intron |
| Mirabello L., 2011 | BMC Cancer | FANCM | 14 | rs4900664 | 0,0035 | intron |
| Savage SA., 2013 | Nat Genet. | FLJ43860 | 8 | rs4907398 | 8,30E-04 | intron |
| Savage SA., 2013 | Nat Genet. | LOC401478 | 8 | rs4909709 | 0,0232 | intron |
| Postel-Vinay S., 2012 | Nat Genet. | SRP14-AS1 | 15 | rs4924410 | 6,60E-09 | intron |
| Mirabello L., 2011 | BMC Cancer | TNFRSF11A | 18 | rs4941129 | 0,0075 | intron |
| Mirabello L., 2011 | BMC Cancer | IGF1R | 15 | rs4966017 | 0,0431 | intron |
| Mirabello L., 2011 | BMC Cancer | SQSTM1 | 5 | rs515110 | 0,0433 | intron |
| Mirabello L., 2011 | BMC Cancer | TGFA | 2 | rs559567 | 0,0305 | intron |
| Mirabello L., 2011 | BMC Cancer | FOSL1 | 11 | rs568617 | 0,0412 | intron |
| Mirabello L., 2011 | BMC Cancer | IGF1 | 12 | rs5742692 | 0,0129 | intron |
| DuBois SG., 2012 | Pediatr Blood Cancer | EWSR1 | 22 | rs5752899 | 0,5743 | intron |
| DuBois SG., 2012 | Pediatr Blood Cancer | EWSR1 | 22 | rs5763131 | 0,4132 | intron |
| Martinelli M., 2016 | Oncotarget | CD99 | Y | rs5939113 | 0,8608 | intron |
| Martinelli M., 2016 | Oncotarget | CD99 | Y | rs5939307 | 0,9893 | intron |
| Martinelli M., 2016 | Oncotarget | CD99 | Y | rs5982572 | 0,5899 | intron |
| Mirabello L., 2011 | BMC Cancer | IGF2R | 6 | rs600324 | 0,0361 | intron |
| Mirabello L., 2011 | BMC Cancer | ATM | 11 | rs618499 | 0,0493 | intron |
| Savage SA., 2013 | Nat Genet. | SLC30A8 | 8 | rs6469674 | 0,0321 | intron |
| Savage SA., 2013 | Nat Genet. | ASAP1 | 8 | rs6470816 | 0,006 | intron |
| Savage SA., 2013 | Nat Genet. | LOC105375760 | 8 | rs6470909 | 0,0482 | intron |
| Grünewald TG., 2015 | Nat Genet. | LOC107984012 | 10 | rs6479846 | 1,0520E-05 | intron |
| Grünewald TG., 2015 | Nat Genet. | LOC107984012 | 10 | rs6479848 | 9,1550E-06 | intron |
| Grünewald TG., 2015 | Nat Genet. | LOC107984012 | 10 | rs6479852 | 1,0520E-05 | intron |
| Martinelli M., 2016 | Oncotarget | CD99 | Y | rs6567640 | 0,6231 | intron |
| Savage SA., 2013 | Nat Genet. | TRAPPC9 | 8 | rs6578056 | 0,0193 | intron |
| Savage SA., 2013 | Nat Genet. | TRAPPC9 | 8 | rs6578065 | 0,0076 | intron |
| Savage SA., 2013 | Nat Genet. | TSNARE1 | 8 | rs6583623 | 0,0358 | intron |
| Mirabello L., 2011 | BMC Cancer | FAF1, CDKN2C | 1 | rs6668495 | 0,0221 | intron |
| Mirabello L., 2011 | BMC Cancer | LHCGR | 2 | rs6711321 | 0,0136 | intron |
| Mirabello L., 2011 | BMC Cancer | FOSL1 | 11 | rs694994 | 0,0402 | intron |
| Mirabello L., 2011 | BMC Cancer | IGFBP3 | 7 | rs6953668 | 0,0091 | intron |
| Mirabello L., 2011 | BMC Cancer | COL1A2 | 12 | rs6970279 | 0,0333 | intron |
| He ML., 2013 | Asian Pac J Cancer Prev. | NPSR1-AS1 | 7 | rs6973569 | 0,4472 | intron |
| Savage SA., 2013 | Nat Genet. | AGO2 | 8 | rs6985156 | 0,0024 | intron |
| Savage SA., 2013 | Nat Genet. | CCDC26 | 8 | rs6985166 | 0,0355 | intron |
| Savage SA., 2013 | Nat Genet. | NDUFB9 | 8 | rs6987343 | 0,0473 | intron |
| Savage SA., 2013 | Nat Genet. | ADCY8 | 8 | rs6988204 | 0,0202 | intron |
| Savage SA., 2013 | Nat Genet. | LOC105375725 | 8 | rs6990281 | 0,0172 | intron |
| Savage SA., 2013 | Nat Genet. | SLC45A4 | 8 | rs6992286 | 0,0147 | intron |
| Savage SA., 2013 | Nat Genet. | NSMCE2 | 8 | rs6992545 | 0,0322 | intron |
| Savage SA., 2013 | Nat Genet. | LOC107986980 | 8 | rs6995884 | 0,0077 | intron |
| Savage SA., 2013 | Nat Genet. | TRAPPC9 | 8 | rs6995955 | 0,0382 | intron |
| Savage SA., 2013 | Nat Genet. | LOC105375730 | 8 | rs6998748 | 0,0227 | intron |
| Savage SA., 2013 | Nat Genet. | SAMD12 | 8 | rs7005426 | 0,038 | intron |
| Savage SA., 2013 | Nat Genet. | TRAPPC9 | 8 | rs7005840 | 0,0111 | intron |
| Savage SA., 2013 | Nat Genet. | TRAPPC9 | 8 | rs7005952 | 0,0141 | intron |
| Savage SA., 2013 | Nat Genet. | TRAPPC9 | 8 | rs7006969 | 0,0273 | intron |
| Savage SA., 2013 | Nat Genet. | SNTB1 | 8 | rs7008488 | 0,0071 | intron |
| Savage SA., 2013 | Nat Genet. | SLC30A8 | 8 | rs7011057 | 0,0195 | intron |
| Savage SA., 2013 | Nat Genet. | LOC105375751 | 8 | rs7012323 | 0,0261 | intron |
| Savage SA., 2013 | Nat Genet. | LOC105375716 | 8 | rs7015627 | 0,0178 | intron |
| Savage SA., 2013 | Nat Genet. | MTSS1 | 8 | rs7016031 | 0,0472 | intron |
| Savage SA., 2013 | Nat Genet. | SNTB1 | 8 | rs7017780 | 0,0202 | intron |
| Mirabello L., 2011 | BMC Cancer | GC | 4 | rs705117 | 0,0153 | intron |
| Martinelli M., 2016 | Oncotarget | CD99 | Y | rs7051896 | 0,5339 | intron |
| Grünewald TG., 2015 | Nat Genet. | LOC107984012 | 10 | rs7071512 | 6,8980E-06 | intron |
| Mirabello L., 2011 | BMC Cancer | FANCM | 14 | rs7141145 | 0,0039 | intron |
| Mirabello L., 2011 | BMC Cancer | HSD17B2 | 16 | rs7192075 | 0,0217 | intron |
| Mirabello L., 2011 | BMC Cancer | TNFRSF11A | 18 | rs7239261 | 0,0466 | intron |
| Savage SA., 2013 | Nat Genet. | LOC105375746 | 8 | rs729005 | 0,0358 | intron |
| Xu H., 2016 | Med Sci Monit. | RASSF1A | 3 | rs72932987 | 0,8405 | intron |
| Savage SA., 2013 | Nat Genet. | TRAPPC9 | 8 | rs729855 | 0,042 | intron |
| Wang K., 2016 | Tumour Biol. | GRM4 | 6 | rs733457 | 0,6897 | intron |
| Savage SA., 2013 | Nat Genet. | ENPP2 | 8 | rs7341607 | 0,034 | intron |
| Mirabello L., 2011 | BMC Cancer | LHCGR | 2 | rs7371084 | 0,0150 | intron |
| Savage SA., 2013 | Nat Genet. | LOC401478 | 8 | rs7386095 | 0,0386 | intron |
| Mirabello L., 2011 | BMC Cancer | IGFBP5 | 2 | rs7420849 | 0,0410 | intron |
| Mirabello L., 2011 | BMC Cancer | GDF2 | 10 | rs743509 | 0,0409 | intron |
| Mirabello L., 2011 | BMC Cancer | PARP4 | 13 | rs750391 | 0,0274 | intron |
| Mirabello L., 2011 | BMC Cancer | UGT1A8 | 2 | rs7556676 | 0,0232 | intron |
| Savage SA., 2013 | Nat Genet. | LOC105375751 | 8 | rs756099 | 0,0254 | intron |
| Savage SA., 2013 | Nat Genet. | KCNK9 | 8 | rs759656 | 0,01 | intron |
| Mirabello L., 2011 | BMC Cancer | COL1A2 | 12 | rs760043 | 0,0014 | intron |
| He ML., 2013 | Asian Pac J Cancer Prev. | PIK3CA | 3 | rs7646409 | 0,0054 | intron |
| Savage SA., 2013 | Nat Genet. | FGF2 | 4 | rs7694627 | 0,0315 | intron |
| Mirabello L., 2011 | BMC Cancer | CCNH | 5 | rs7702564 | 0,0459 | intron |
| Mirabello L., 2011 | BMC Cancer | COL1A2 | 12 | rs7788014 | 0,0495 | intron |
| Savage SA., 2013 | Nat Genet. | COL1A2 | 7 | rs7805430 | 0,0447 | intron |
| Savage SA., 2013 | Nat Genet. | MTSS1 | 8 | rs7813190 | 2,50E-04 | intron |
| Savage SA., 2013 | Nat Genet. | TRAPPC9 | 8 | rs7819785 | 0,0059 | intron |
| Savage SA., 2013 | Nat Genet. | NSMCE2 | 8 | rs7820471 | 0,0361 | intron |
| Savage SA., 2013 | Nat Genet. | TRAPPC9 | 8 | rs7823724 | 0,0482 | intron |
| Savage SA., 2013 | Nat Genet. | AGO2 | 8 | rs7824304 | 0,007 | intron |
| Savage SA., 2013 | Nat Genet. | SNTB1 | 8 | rs7827807 | 0,008 | intron |
| Mirabello L., 2011 | BMC Cancer | CYP11B1 | 8 | rs7833415 | 0,0315 | intron |
| Savage SA., 2013 | Nat Genet. | NSMCE2 | 8 | rs7837996 | 0,0146 | intron |
| Savage SA., 2013 | Nat Genet. | EIF3H | 8 | rs7846316 | 0,034 | intron |
| Grünewald TG., 2015 | Nat Genet. | LOC107984012 | 10 | rs7915755 | 6,7750E-03 | intron |
| Mirabello L., 2011 | BMC Cancer | IGF1 | 12 | rs7956547 | 0,0019 | intron |
| Naumov VA., 2012 | Bull Exp Biol Med. | IGF1 | 12 | rs7956547 | 0,8951 | intron |
| Ruza E., 2003 | J Pediatr Hematol Oncol | VDR | 12 | rs7975232 | 0,5214 | intron |
| Mirabello L., 2011 | BMC Cancer | TDP1 | 14 | rs8010627 | 0,0233 | intron |
| Postel-Vinay S., 2012 | Nat Genet. | SRP14-AS1 | 15 | rs8026641 | 3,70E-04 | intron |
| Mirabello L., 2011 | BMC Cancer | DUT | 15 | rs8037626 | 0,0231 | intron |
| Savage SA., 2013 | Nat Genet. | LOC105370978, FAM174B | 15 | rs8043510 | 4,73E-04 | intron |
| Mirabello L., 2011 | BMC Cancer | HSD17B2 | 16 | rs8045494 | 0,0042 | intron |
| Mirabello L., 2011 | BMC Cancer | HSD17B2 | 16 | rs8048090 | 0,0439 | intron |
| Mirabello L., 2011 | BMC Cancer | HSD17B2 | 16 | rs8049423 | 0,0217 | intron |
| Mirabello L., 2011 | BMC Cancer | HSD17B2 | 16 | rs8052451 | 0,0351 | intron |
| Savage SA., 2013 | Nat Genet. | WWOX | 16 | rs8057015 | 3,71E-03 | intron |
| Mirabello L., 2011 | BMC Cancer | HSD17B2 | 16 | rs8059915 | 0,0364 | intron |
| Mirabello L., 2011 | BMC Cancer | RPA1 | 17 | rs8067195 | 0,0175 | intron |
| Lu H., 2015 | Tumor Biol. | PRKCG | 19 | rs8103851 | 0,645 | intron |
| Zhang Y., 2014 | Tumor Biol. | PRKCG | 19 | rs8103851 | <0.00001 | intron |
| Mirabello L., 2011 | BMC Cancer | HSD17B3 | 9 | rs8190534 | 0,0497 | intron |
| Mirabello L., 2011 | BMC Cancer | HSD17B2 | 16 | rs8191167 | 0,0347 | intron |
| Mirabello L., 2011 | BMC Cancer | NEIL2 | 8 | rs8191604 | 0,0123 | intron |
| Savage SA., 2013 | Nat Genet. | ESR1 | 6 | rs851982 | 0,0389 | intron |
| Mirabello L., 2011 | BMC Cancer | GLDN, CYP19A1 | 15 | rs868475 | 0,0259 | intron |
| Mirabello L., 2011 | BMC Cancer | UGT1A8 | 2 | rs871514 | 0,0264 | intron |
| Savage SA., 2013 | Nat Genet. | TRAPPC9 | 8 | rs876745 | 0,0212 | intron |
| Savage SA., 2013 | Nat Genet. | LOC107986980 | 8 | rs894087 | 0,0462 | intron |
| Savage SA., 2013 | Nat Genet. | LOC401478 | 8 | rs907461 | 0,0246 | intron |
| Oliveira ID., 2007 | J Pediatr Hematol Oncol. | LOC100287329, LTA | 6 | rs909253 | 0,4902 | intron |
| Mirabello L., 2011 | BMC Cancer | BMP6 | 6 | rs911749 | 0,0371 | intron |
| Savage SA., 2013 | Nat Genet. | SLC45A4 | 8 | rs919873 | 0,0212 | intron |
| Grünewald TG., 2015 | Nat Genet. | LOC107984012 | 10 | rs925307 | 5,4960E-06 | intron |
| Savage SA., 2013 | Nat Genet. | EXT1 | 8 | rs9297575 | 0,0114 | intron |
| Savage SA., 2013 | Nat Genet. | ADCY8 | 8 | rs9297816 | 0,0319 | intron |
| Ruza E., 2003 | J Pediatr Hematol Oncol | ER | 6 | rs9340799 | 0,4739 | intron |
| Mirabello L., 2011 | BMC Cancer | IGFBP2 | 2 | rs9341105 | 0,0122 | intron |
| Walsh KM., 2016 | Carcinogenesis. | OBF-C1, STN1 | 10 | rs9420907 | 0,0094 | intron |
| Grünewald TG., 2015 | Nat Genet. | LOC107984012 | 10 | rs944684 | 6,8980E-06 | intron |
| Postel-Vinay S., 2012 | Nat Genet. | LOC107984012 | 10 | rs944684 | 2,10E-12 | intron |
| Mirabello L., 2011 | BMC Cancer | IGF2R | 6 | rs9456484 | 0,0124 | intron |
| Grünewald TG., 2015 | Nat Genet. | LOC107984012 | 10 | rs953026 | 1,0520E-05 | intron |
| Grünewald TG., 2015 | Nat Genet. | LOC107984012 | 10 | rs965128 | 9,4780E-06 | intron |
| Mirabello L., 2011 | BMC Cancer | IGF1 | 12 | rs9651925 | 0,0194 | intron |
| Savage SA., 2013 | Nat Genet. | PVT1 | 8 | rs9656968 | 0,0462 | intron |
| Savage SA., 2013 | Nat Genet. | LOC401478 | 8 | rs9657451 | 0,0341 | intron |
| Savage SA., 2013 | Nat Genet. | CREB5 | 7 | rs9691873 | 9,61E-05 | intron |
| Savage SA., 2013 | Nat Genet. | AGO2 | 8 | rs9694342 | 0,0138 | intron |
| Savage SA., 2013 | Nat Genet. | TRAPPC9 | 8 | rs9774091 | 0,0039 | intron |
| Mirabello L., 2011 | BMC Cancer | PTEN | 10 | rs9783238 | 0,0155 | intron |
| Mirabello L., 2011 | BMC Cancer | POLK | 5 | rs979182 | 0,0478 | intron |
| Mirabello L., 2011 | BMC Cancer | IGF2BP2 | 2 | rs9850770 | 0,0320 | intron |
| He ML., 2013 | Asian Pac J Cancer Prev. | PIK3CA | 3 | rs9866361 | 0,3767 | intron |
| Savage SA., 2013 | Nat Genet. | PHF20L1 | 8 | rs988068 | 0,0492 | intron |
| Savage SA., 2007 | Cancer Epidemiol Biomarkers Prev. | IGFR2 | 6 | rs998074 | 0,002 | intron |
| Mirabello L., 2011 | BMC Cancer | BRIP1 | 17 | rs12937080 | 0,0273 | intron |
| Mirabello L., 2011 | BMC Cancer | FLJ35220 | 17 | rs12938834 | 0,0059 | intron |
| Savage SA., 2007 | Pediat Blood Cancer | TP53 | 17 | rs12947788 | 0,3515 | intron |
| Ru JY., 2015 | Int J Clin Exp Pathol. | TP53 | 17 | rs12951053 | 0,0252 | intron |
| DuBois SG., 2012 | Pediatr Blood Cancer | EWSR1 | 22 | rs131184 | 0,4485 | intron |
| Mirabello L., 2011 | BMC Cancer | FGFR2 | 10 | rs3135819 | 0,0132 | intron variant, downstream variant 500B |
| Mirabello L., 2011 | BMC Cancer | DUT | 15 | rs16960758 | 0,0172 | intron variant,nc transcript variant |
| Mirabello L., 2011 | BMC Cancer | IGF1 | 12 | rs2195240 | 0,0462 | intron variant,nc transcript variant |
| Mirabello L., 2011 | BMC Cancer | HSD17B1 | 17 | rs2676530 | 0,0378 | intron variant,nc transcript variant |
| Walsh KM., 2016 | Carcinogenesis. | CTC1 | 17 | rs3027234 | 0,7759 | intron variant,nc transcript variant |
| Mirabello L., 2011 | BMC Cancer | LIG1 | 19 | rs3730872 | 0,0276 | intron variant,nc transcript variant |
| Mirabello L., 2011 | BMC Cancer | HSD17B1 | 17 | rs676387 | 0,0176 | intron variant,nc transcript variant |
| Qi Y., 2016 | Tumour Biol. | IL6 | 7 | rs1800796 | 0,567 | intron variant,nc transcript variant,upstream variant 2KB |
| Mirabello L., 2011 | BMC Cancer | CDKN1B | 12 | rs10492237 | 0,0196 | intron variant,upstream variant 2KB |
| Mirabello L., 2011 | BMC Cancer | PARP2 | 14 | rs11622655 | 0,0341 | intron variant,upstream variant 2KB |
| Tang YJ., 2014 | Medicine | IL27 | 16 | rs153109 | 0,3476 | intron variant,upstream variant 2KB |
| Savage SA., 2013 | Nat Genet. | LOC105370978, FAM174B | 15 | rs17523778 | 2,91E-03 | intron variant,upstream variant 2KB |
| Koshkina NV., 2007 | [J Pediatr Hematol Oncol.](http://journals.lww.com/jpho-online/pages/default.aspx?desktopMode=true) | FAS | 10 | rs1800682 | 0,3917 | intron variant,upstream variant 2KB |
| Oliveira ID., 2007 | J Pediatr Hematol Oncol. | IL6 | 7 | rs1800795 | 0,3391 | intron variant,upstream variant 2KB |
| Qi Y., 2016 | Tumour Biol. | IL6 | 7 | rs1800795 | 0,0002 | intron variant,upstream variant 2KB |
| Mirabello L., 2011 | BMC Cancer | CYP19A1 | 15 | rs1902584 | 0,0197 | intron variant,upstream variant 2KB |
| Xu H., 2016 | Med Sci Monit. | RASSF1A | 3 | rs1989839 | 0,0034 | intron variant,upstream variant 2KB |
| Mirabello L., 2011 | BMC Cancer | RPA3 | 7 | rs2024374 | 0,0227 | intron variant,upstream variant 2KB |
| Wang J., 2013 | DNA Cell Biol. | IL12A | 3 | rs2243115 | 0,7419 | intron variant,upstream variant 2KB |
| Alhopuro P., 2005 | J Med Genet. | MDM2 | 12 | rs2279744 | 0,9044 | intron variant,upstream variant 2KB |
| Ito M., 2010 | Clin Cancer Res. | MDM2 | 12 | rs2279744 | 0,1667 | intron variant,upstream variant 2KB |
| Thurow HS., 2013 | Mol Biol Rep . | MDM2 | 12 | rs2279744 | 0,1111 | intron variant,upstream variant 2KB |
| Toffoli G., 2009 | Clin. Cancer Res. | MDM2 | 12 | rs2279744 | 0,0021 | intron variant,upstream variant 2KB |
| Mirabello L., 2011 | BMC Cancer | RHBDF1, MPG | 16 | rs2288490 | 0,0346 | intron variant,upstream variant 2KB |
| DuBois SG., 2012 | Pediatr Blood Cancer | EWSR1 | 22 | rs2301291 | 0,4386 | intron variant,upstream variant 2KB |
| Silva DSBS., 2012 | Gene | EWSR1 | 22 | rs2301291 | 0,5223 | intron variant,upstream variant 2KB |
| Mirabello L., 2011 | BMC Cancer | UGT1A8 | 2 | rs3755319 | 0,0322 | intron variant,upstream variant 2KB |
| Mirabello L., 2011 | BMC Cancer | GNRH2 | 20 | rs3761243 | 0,0020 | intron variant,upstream variant 2KB |
| Naumov VA., 2012 | Bull Exp Biol Med. | GNRH2 | 20 | rs3761243 | 0,1362 | intron variant,upstream variant 2KB |
| Mirabello L., 2011 | BMC Cancer | UGT1A8 | 2 | rs3806597 | 0,0259 | intron variant,upstream variant 2KB |
| Mirabello L., 2011 | BMC Cancer | HSD17B8, RXRB | 6 | rs383711 | 0,0210 | intron variant,upstream variant 2KB |
| Savage SA., 2013 | Nat Genet. | LOC105370978, FAM174B | 15 | rs432364 | 9,93E-05 | intron variant,upstream variant 2KB |
| Mirabello L., 2011 | BMC Cancer | ENDOV, FLJ35220 | 17 | rs4603608 | 0,0074 | intron variant,upstream variant 2KB |
| Mirabello L., 2011 | BMC Cancer | POLM | 7 | rs4640970 | 0,0359 | intron variant,upstream variant 2KB |
| Tian Q., 2013 | Eur J Surg Oncol. | miR-34b/c | 11 | rs4938723 | 0,0036 | intron variant,upstream variant 2KB |
| Mirabello L., 2011 | BMC Cancer | DRAXIN, MAD2L2 | 1 | rs6694489 | 0,0434 | intron variant,upstream variant 2KB |
| Mirabello L., 2011 | BMC Cancer | POLM | 7 | rs6955679 | 0,0162 | intron variant,upstream variant 2KB |
| Mirabello L., 2011 | BMC Cancer | FGFR1 | 8 | rs6983315 | 0,0153 | intron variant,upstream variant 2KB |
| Ru JY., 2015 | Int J Clin Exp Pathol. | TP53 | 17 | rs8064946 | 0,5417 | intron variant,upstream variant 2KB |
| Savage SA., 2007 | Pediat Blood Cancer | TP53 | 17 | rs8079544 | 0,3611 | intron variant,upstream variant 2KB |
| Mirabello L., 2011 | BMC Cancer | PARP4 | 13 | rs9511308 | 0,0099 | intron variant,upstream variant 2KB |
| Zhi LQ., 2013 | Tumor Biol. | RECQL5 | 17 | rs820200 | 0,3741 | intron variant,upstream variant 2KB,utr variant 3 prime |
| Savage SA., 2007 | Pediat Blood Cancer | TP53 | 17 | rs1642785 | 0,5731 | intron variant,upstream variant 2KB,utr variant 5 prime |
| Mirabello L., 2011 | BMC Cancer | EIF2AK2 | 2 | rs2307466 | 0,0104 | intron variant,upstream variant 2KB,utr variant 5 prime |
| Savage SA., 2013 | Nat Genet. | ESR1 | 6 | rs3020333 | 0,049 | intron variant,upstream variant 2KB,utr variant 5 prime |
| Zhi LQ., 2013 | Tumor Biol. | RECQL5 | 17 | rs4789223 | <0.00001 | intron variant,upstream variant 2KB,utr variant 5 prime |
| Mirabello L., 2011 | BMC Cancer | FGF2, NUDT6 | 4 | rs3804158 | 0,0441 | intron variant,utr variant 3 prime |
| Wang J., 2013 | DNA Cell Biol. | IL12A | 3 | rs568408 | 0,0663 | intron variant,utr variant 3 prime |
| Mirabello L., 2011 | BMC Cancer | IGF1 | 12 | rs5742714 | 0,0037 | intron variant,utr variant 3 prime |
| Savage SA., 2007 | Pediat Blood Cancer | TP53 | 17 | rs2909430 | 0,7304 | intron variant,utr variant 5 prime |
| DuBois SG., 2012 | Pediatr Blood Cancer | EWSR1 | 22 | rs3788410 | 0,19 | intron variant,utr variant 5 prime |
| DuBois SG., 2012 | Pediatr Blood Cancer | EWSR1 | 22 | rs9613855 | 0,55 | intron variant,utr variant 5 prime |
| Ru JY., 2015 | Int J Clin Exp Pathol. | TP53 | 17 | rs9895829 | 0,8667 | intron variant,utr variant 5 prime |
| Savage SA., 2007 | Pediat Blood Cancer | TP53 | 17 | rs9895829 | 0,5007 | intron variant,utr variant 5 prime |
| Mirabello L., 2011 | BMC Cancer | BRIP1 | 17 | rs9908659 | 0,0048 | intron variant,utr variant 5 prime |
| Savage SA., 2013 | Nat Genet. | BOP1, HSF1 | 8 | rs11998675 | 0,043 | intron, near-gene-5' |
| Savage SA., 2013 | Nat Genet. | BOP1, HSF1 | 8 | rs4977203 | 0,0214 | intron, near-gene-5' |
| Savage SA., 2013 | Nat Genet. | TATDN1, NDUFB9, MTSS1 | 8 | rs7007115 | 0,034 | intron, near-gene-5' |
| Barnette P., 2004 | Cancer Epidemiol Biomarkers Prev. | GSTP1 | 11 | rs1138272 |  | missense |
| Li L., 2016 | Genet Mol Res. | CYP1A1 | 15 | rs1048943 | 0,008 | missense |
| Grünewald TG., 2015 | Nat Genet. | ADO | 10 | rs10995311 | 3,3030E-05 | missense |
| Wang J., 2011 | DNA Cell Biol. | CD86 | 3 | rs1129055 | 0,0172 | missense |
| Wang W., 2011 | DNA Cell Biol. | CD86 | 3 | rs1129055 | 0,0001 | missense |
| Savage SA., 2013 | Nat Genet. | KLHL38 | 8 | rs11784175 | 0,0232 | missense |
| Aoyama T., 2002 | Cancer Letters | NFAT1 | 20 | rs12479626 | 0,2243 | missense |
| Zhang N., 2016 | Onco Targets Ther. | WWOX | 16 | rs12918952 | 0,8853 | missense |
| He J., 2013 | Endocrine | CTLA4 | 2 | rs144988077 | 0,0102 | missense |
| Barnette P., 2004 | Cancer Epidemiol Biomarkers Prev. | GSTP1 | 11 | rs1695 |  | missense |
| Qu WR., 2016 | Genet Mol Res | GSTP1 | 11 | rs1695 |  | missense |
| Biason P., 2016 | Pharmacogenomics J. | ERCC5 | 13 | rs17655 | 0,4528 | missense |
| Le Morvan V., 2006 | Int. J. Cancer | ERCC5 | 13 | rs17655 | 0,0001 | missense |
| Le Morvan V., 2006 | Int. J. Cancer | ERCC5 | 13 | rs17655 | 0,0031 | missense |
| Le Morvan V., 2006 | Int. J. Cancer | ERCC5 | 13 | rs17655 | 0,0465 | missense |
| Le Morvan V., 2006 | Int. J. Cancer | ERCC5 | 13 | rs17655 | 0,3157 | missense |
| Le Morvan V., 2006 | Int. J. Cancer | ERCC5 | 13 | rs17655 | 0,3882 | missense |
| Tang YJ., 2014 | Medicine | IL27 | 16 | rs17855750 | 0,9024 | missense |
| Nakayama R., 2008 | Cancer Science | XRCC1 | 19 | rs1799782 | 0,073 | missense |
| Xu S., 2014 | DNA Cell Biol. | TGFB1 | 19 | rs1800470 | 0,004 | missense |
| Ozger H., 2008 | Folia Biologica | MTHFR | 1 | rs1801133 | 0,8608 | missense |
| Ozger H., 2008 | Folia Biologica | MTHFR | 1 | rs1801133 | 0,6728 | missense |
| Tang YJ., 2014 | Medicine | IL27 | 16 | rs181206 | 0,8194 | missense |
| Wu Y., 2015 | Tumor Biol. | TGFB1 | 19 | rs1982073 | 0,4825 | missense |
| Ruza E., 2003 | J Pediatr Hematol Oncol | VDR | 12 | rs2228570 | 0,9047 | missense |
| Grünewald TG., 2015 | Nat Genet. | ADO | 10 | rs2236295 | 2,6280E-05 | missense |
| Kelley MJ., 2014 | Hum. Genet. | T | 6 | rs2305089 | <0.0001 | missense |
| Pillay N., 2012 | Nat Genet. | T | 6 | rs2305089 | 2.8E10-4 | missense |
| Pillay N., 2012 | Nat Genet. | T | 6 | rs2305089 | 4.4E10-9 | missense |
| Wu Z., 2013 | Int. J. Mol. Sci. | T | 6 | rs2305089 | 0,7124 | missense |
| Feng D., 2013 | Genet Test Mol Biomarkers | CTLA4 | 2 | rs231775 | 0,0045 | missense |
| Liu Y., 2011 | DNA Cell Biol. | CTLA4 | 2 | rs231775 | 0,0289 | missense |
| Wang W., 2011 | Genet Test Mol Biomarkers | CTLA4 | 2 | rs231775 | 0,0147 | missense |
| Yang S., 2012 | Genet Test Mol Biomarkers | CTLA4 | 2 | rs231775 | 0,0271 | missense |
| Cong Y., 2014 | Tumor Biol. | BMP2 | 20 | rs235768 | 0,7279 | missense |
| Savage SA., 2013 | Nat Genet. | FLJ43860 | 8 | rs2748416 | 0,0393 | missense |
| Oliveira ID., 2007 | J Pediatr Hematol Oncol. | PECAM-1 | 17 | rs281865545 | 0,307 | missense |
| Mirabello L., 2011 | BMC Cancer | CYP19A1 | 15 | rs28757184 | 0,0064 | missense |
| Kelley MJ., 2014 | Hum. Genet. | T | 6 | rs3127328 | 0,003 | missense |
| Zhang N., 2016 | Onco Targets Ther. | WWOX | 16 | rs3764340 | 0,0015 | missense |
| Savage SA., 2013 | Nat Genet. | TRMT12 | 8 | rs3812475 | 0,0166 | missense |
| Kelley MJ., 2014 | Hum. Genet. | T | 6 | rs3816300 | 0,69 | missense |
| Mirabello L., 2011 | BMC Cancer | ERCC6 | 10 | rs4253211 | 0,0464 | missense |
| Savage SA., 2013 | Nat Genet. | BC042052, MYC | 8 | rs4645959 | 0,0199 | missense |
| Savage SA., 2007 | Cancer Epidemiol Biomarkers Prev. | IGFR2 | 6 | rs629849 | 0,154 | missense |
| Ergen A., 2010 | Mol Biol Rep . | PON1 | 7 | rs662 | 0,0679 | missense |
| Adiguzel M., 2016 | Indian J Exp Biol. | MMP3 | 11 | rs679620 | 0,1722 | missense |
| Miao C., 2015 | Sci. Rep. | PRAMEF13 | 1 | rs71183793 | 0,0586 | missense |
| Ergen A., 2010 | Mol Biol Rep . | PON1 | 7 | rs854560 | 0,1785 | missense |
| Nakayama R., 2008 | Cancer Science | WRN | 8 | rs1346044 | 0,005 | missense, downstream variant 500B |
| Wang K., 2014 | Biomedical Reports | WRN | 8 | rs1346044 | 0,4364 | missense, downstream variant 500B |
| Wang K., 2014 | Biomedical Reports | WRN | 8 | rs1346044 | 0,6103 | missense, downstream variant 500B |
| Savage SA., 2013 | Nat Genet. | FAM208B, GDI2 | 10 | rs2797501 | 7,88E-06 | missense, downstream variant 500B |
| Biason P., 2012 | Pharmacogenomics J. | ERCC2 | 19 | rs13181 | 0,6089 | missense, downstream variant 500B, nc transcript variant |
| Le Morvan V., 2006 | Int. J. Cancer | ERCC2 | 19 | rs13181 | 0,1798 | missense, downstream variant 500B, nc transcript variant |
| Le Morvan V., 2006 | Int. J. Cancer | ERCC2 | 19 | rs13181 | 0,4928 | missense, downstream variant 500B, nc transcript variant |
| Le Morvan V., 2006 | Int. J. Cancer | ERCC2 | 19 | rs13181 | 0,3292 | missense, downstream variant 500B, nc transcript variant |
| Le Morvan V., 2006 | Int. J. Cancer | ERCC2 | 19 | rs13181 | 0,4928 | missense, downstream variant 500B, nc transcript variant |
| Le Morvan V., 2006 | Int. J. Cancer | ERCC2 | 19 | rs13181 | 0,5572 | missense, downstream variant 500B, nc transcript variant |
| Ma X., 2016 | Genet Mol Res. | ERCC2 | 19 | rs13181 | 0,1643 | missense, downstream variant 500B, nc transcript variant |
| Hu Z., 2015 | Genet Test Mol Biomarkers | MDM2 | 12 | rs11177386 | 0,0374 | missense, intron variant |
| Nakayama R., 2008 | Cancer Science | MBD4 | 3 | rs140693 | 0,286 | missense, intron variant |
| Saito T., 2000 | Int. J. Cancer | CX37 | 1 | rs1764391 | 0,7389 | missense, intron variant |
| Bilbao-Aldaiturriaga N., 2015 | Pediat Blood Cancer | CNOT4 | 7 | rs3812265 | 0,0752 | missense, intron variant |
| Aoyama T., 2002 | Cancer Letters | NFAT1 | 20 | rs55980737 | 0,7889 | missense, intron variant |
| Guo J., 2015 | Genet Mol Res. | XRCC3, KLC1 | 14 | rs861539 | 0,0092 | missense, intron variant |
| Yang LB., 2015 | Int J Clin Exp Pathol. | XRCC3, KLC1 | 14 | rs861539 | 0,0039 | missense, intron variant |
| Koshkina NV., 2007 | [J Pediatr Hematol Oncol.](http://journals.lww.com/jpho-online/pages/default.aspx?desktopMode=true) | FAS | 10 | rs2234767 | 0,5984 | missense, intron variant, nc transcript variant,upstream variant 2KB |
| Mirabello L., 2011 | BMC Cancer | LIG3 | 17 | rs931196 | 0,0453 | missense, intron variant, upstream variant 2KB |
| Xin DJ., 2015 | Int J Clin Exp Pathol. | HER2 | 17 | rs1136201 | 0,0135 | missense,nc transcript variant |
| Miao C., 2015 | Sci. Rep. | IGFN1 | 1 | rs11803067 | 0,5774 | missense,nc transcript variant |
| Mirabello L., 2011 | BMC Cancer | FANCM | 14 | rs1367580 | 0,0031 | missense,nc transcript variant |
| Biason P., 2013 | Pharmacogenomics J. | ERCC2 | 19 | rs1799793 | 0,0116 | missense,nc transcript variant |
| Ma X., 2016 | Genet Mol Res. | ERCC2 | 19 | rs1799793 | 0,2777 | missense,nc transcript variant |
| Yang W., 2014 | Med Oncol. | ITGA3 | 17 | rs2230392 | 0,0031 | missense,nc transcript variant |
| Miao C., 2015 | Sci. Rep. | CD27 | 12 | rs74348171 | 0,5792 | missense,nc transcript variant |
| Miao C., 2015 | Sci. Rep. | FPGS | 9 | rs10760502 | 0,0007 | missense,nc transcript variant,upstream variant 2KB,utr variant 5 prime |
| Xin DJ., 2015 | Int J Clin Exp Pathol. | HER2 | 17 | rs1058808 | 0,0032 | missense,nc transcript variant,utr variant 3 prime |
| Biason P., 2014 | Pharmacogenomics J. | CD3EAP, ERCC1 | 19 | rs3212986 | 0,1835 | missense,nc transcript variant,utr variant 3 prime |
| Zhang N., 2016 | Onco Targets Ther. | WWOX | 16 | rs383362 | 0,0108 | missense,nc transcript variant,utr variant 3 prime |
| Nakayama R., 2008 | Cancer Science | REV1 | 2 | rs3087399 | 0,049 | missense,nc transcript variant,utr variant 5 prime |
| Almeida PSR., 2008 | Gen Mol Res. | TP53 | 17 | rs1042522 | 0,2208 | missense,upstream variant 2KB |
| Ru JY., 2015 | Int J Clin Exp Pathol. | TP53 | 17 | rs1042522 | 0,0062 | missense,upstream variant 2KB |
| Savage SA., 2007 | Pediat Blood Cancer | TP53 | 17 | rs1042522 | 0,5906 | missense,upstream variant 2KB |
| Thurow HS., 2013 | Mol Biol Rep . | TP53 | 17 | rs1042522 | 0,4738 | missense,upstream variant 2KB |
| Toffoli G., 2009 | Clin. Cancer Res. | TP53 | 17 | rs1042522 | 0,0453 | missense,upstream variant 2KB |
| Ito M., 2010 | Clin Cancer Res. | TP53 | 17 | rs1042522 | 0,9648 | missense,upstream variant 2KB |
| Liu Y., 2012 | PLoS ONE | LOX | 5 | rs1800449 | 0,0129 | missense,upstream variant 2KB |
| Nakayama R., 2008 | Cancer Science | MSH6 | 2 | rs1042821 | 0,75 | missense,upstream variant 2KB,utr variant 5 prime |
| Dong YZ., 2015 | Genet Mol Res. | RECQL5 | 17 | rs820196 | 0,0253 | missense,upstream variant 2KB,utr variant 5 prime |
| Zhi LQ., 2013 | Tumor Biol. | RECQL5 | 17 | rs820196 | 0,0632 | missense,upstream variant 2KB,utr variant 5 prime |
| He J., 2013 | Endocrine Journal | MDM2 | 12 | rs201821879 | 0,0034 | missense,utr variant 5 prime |
| Mirabello L., 2010 | Carcinogenesis | CASC8, CCAT2 | 8 | rs6983267 | 0,4528 | nc transciprt intron variant,nc transcript variant |
| Savage SA., 2013 | Nat Genet. | CASC8 | 8 | rs10086608 | 0,0158 | nc transcript intron variant |
| Savage SA., 2013 | Nat Genet. | CASC8 | 8 | rs10505473 | 0,0161 | nc transcript intron variant |
| Mirabello L., 2010 | Carcinogenesis | CASC8 | 8 | rs10505477 | 0,5257 | nc transcript intron variant |
| Mirabello L., 2010 | Carcinogenesis | CASC8 | 8 | rs10808555 | 0,1756 | nc transcript intron variant |
| Savage SA., 2013 | Nat Genet. | CASC21 | 8 | rs11777807 | 4,60E-05 | nc transcript intron variant |
| Mirabello L., 2010 | Carcinogenesis | CASC21, CASC9 | 8 | rs13281615 | 0,2993 | nc transcript intron variant |
| Mirabello L., 2010 | Carcinogenesis | CASC8 | 8 | rs1447295 | 0,4944 | nc transcript intron variant |
| Savage SA., 2013 | Nat Genet. | LINC00824 | 8 | rs1516980 | 0,031 | nc transcript intron variant |
| Savage SA., 2013 | Nat Genet. | CASC21 | 8 | rs17378189 | 8,16E-04 | nc transcript intron variant |
| Savage SA., 2013 | Nat Genet. | LINC01108 | 6 | rs17435970 | 6,97E-03 | nc transcript intron variant |
| Savage SA., 2013 | Nat Genet. | CASC8, CASC21 | 8 | rs17464492 | 0,0381 | nc transcript intron variant |
| Mirabello L., 2010 | Carcinogenesis | CASC21 | 8 | rs185852 | 0,119 | nc transcript intron variant |
| Savage SA., 2013 | Nat Genet. | CASC21 | 8 | rs412835 | 8,50E-04 | nc transcript intron variant |
| Savage SA., 2013 | Nat Genet. | CASC8 | 8 | rs4871799 | 8,53E-04 | nc transcript intron variant |
| Mirabello L., 2010 | Carcinogenesis | CASC21, CASC8 | 8 | rs620861 | 0,0883 | nc transcript intron variant |
| Savage SA., 2013 | Nat Genet. | CASC21, CASC8 | 8 | rs6984900 | 0,0048 | nc transcript intron variant |
| Savage SA., 2013 | Nat Genet. | CASC8 | 8 | rs7012462 | 0,0036 | nc transcript intron variant |
| Mirabello L., 2010 | Carcinogenesis | CASC8 | 8 | rs7014346 | 0,2365 | nc transcript intron variant |
| Mirabello L., 2010 | Carcinogenesis | CASC21, CASC8 | 8 | rs896324 | 0,0122 | nc transcript intron variant |
| Savage SA., 2013 | Nat Genet. | CASC8 | 8 | rs921146 | 0,0152 | nc transcript intron variant |
| Mirabello L., 2010 | Carcinogenesis | CASC11 | 8 | rs9642880 | 0,066 | nc transcript intron variant |
| Nakayama R., 2008 | Cancer Science | PARP-1/ADPRT, LOC105370531 | 14 | rs1136471 | 0,098 | nc transcript variant |
| Savage SA., 2013 | Nat Genet. | LOC105375741 | 8 | rs1367500 | 0,0381 | nc transcript variant |
| Lv H., 2014 | Mol Med Rep. | miR-34a | 1 | rs2910164 | 0,1304 | nc transcript variant |
| Savage SA., 2013 | Nat Genet. | RNF139-AS1 TRMT12, CR93366 | 8 | rs4128468 | 0,0372 | nc transcript variant |
| Shi ZW., 2016 | Cancer Biomark. | hsa-miR-124a | 8 | rs531564 |  | nc transcript variant |
| Lv H., 2014 | Mol Med Rep. | miR-34a | 1 | rs72631823 | <0.00001 | nc transcript variant |
| Savage SA., 2013 | Nat Genet. | CASC19 AK125310 | 8 | rs2466032 | 0,0012 | nc transcript variant |
| Mirabello L., 2011 | BMC Cancer | PTEN | 10 | rs478839 | 0,0052 | nc transcript variant, downstream variant 500B |
| Savage SA., 2013 | Nat Genet. | RNF139-AS1,RNF139 | 8 | rs3812472 | 0,0294 | nc transcript variant,upstream variant 2KB |
| Zhang N., 2016 | Onco Targets Ther. | WWOX | 16 | rs12828 | 0,4462 | nc transcript variant,utr variant 3 prime |
| Gloudemans T. 1993 | Cancer Res. | IGF2 | 12 | rs680 | 0,1005 | nc transcript variant,utr variant 3 prime |
| Mirabello L., 2010 | Carcinogenesis | CASC8, CASC21 | 8 | rs12155672 | 0,0729 | nc transcript, intergene |
| Savage SA., 2013 | Nat Genet. | CASC8, CASC21 | 8 | rs13258742 | 0,0022 | nc transcript, intergene |
| Mirabello L., 2010 | Carcinogenesis | CASC8, CASC21 | 8 | rs17766217 | 0,0412 | nc transcript, intergene |
| Mirabello L., 2010 | Carcinogenesis | CASC8 | 8 | rs4242382 | 0,473 | nc transcript, intergene |
| Savage SA., 2013 | Nat Genet. | CASC8 | 8 | rs6991990 | 0,0382 | nc transcript, intergene |
| Savage SA., 2013 | Nat Genet. | CASC8 | 8 | rs7004374 | 0,0231 | nc transcript, intergene |
| Mirabello L., 2010 | Carcinogenesis | CASC8 | 8 | rs7017300 | 0,176 | nc transcript, intergene |
| Mirabello L., 2010 | Carcinogenesis | CASC8 | 8 | rs7837688 | 0,615 | nc transcript, intergene |
| Savage SA., 2013 | Nat Genet. | WDYHV1 | 8 | rs10505435 | 0,0069 | synonymous codon |
| Kelley MJ., 2014 | Hum. Genet. | T | 6 | rs1056048 | 0,002 | synonymous codon |
| Savage SA., 2013 | Nat Genet. | ENPP2 | 8 | rs1058913 | 0,0021 | synonymous codon |
| Savage SA., 2013 | Nat Genet. | EEF1D | 8 | rs1062391 | 0,0186 | synonymous codon |
| Savage SA., 2013 | Nat Genet. | TRAPPC9 | 8 | rs11166965 | 0,0242 | synonymous codon |
| Martinelli M., 2016 | Oncotarget | CD99 | Y | rs1136447 | 0,6099 | synonymous codon |
| He Y., 2014 | Int Orthop. | IL1B | 2 | rs1143634 | 0,5093 | synonymous codon |
| Biason P., 2015 | Pharmacogenomics J. | ERCC1 | 19 | rs11615 | 0,3198 | synonymous codon |
| Savage SA., 2007 | Cancer Epidemiol Biomarkers Prev. | IGFR2 | 6 | rs1570070 | 0,8093 | synonymous codon |
| He J., 2013 | Endocrine | CTLA4 | 2 | rs16840275 | 0,0118 | synonymous codon |
| Mirabello L., 2011 | BMC Cancer | UGT1A8 | 2 | rs17863803 | 0,0445 | synonymous codon |
| Mirabello L., 2011 | BMC Cancer | ERCC4 | 16 | rs1799801 | 0,0298 | synonymous codon |
| Savage SA., 2007 | Pediat Blood Cancer | TP53 | 17 | rs1800372 | 0,2061 | synonymous codon |
| Mirabello L., 2011 | BMC Cancer | ATM | 11 | rs1800889 | 0,0278 | synonymous codon |
| He J., 2013 | Endocrine Journal | MDM2 | 12 | rs199812774 | 0,0306 | synonymous codon |
| Savage SA., 2013 | Nat Genet. | AGO2 | 8 | rs2293939 | 0,0253 | synonymous codon |
| Lu H., 2015 | Tumor Biol. | PRKCG | 19 | rs2547362 | 0,295 | synonymous codon |
| Kelley MJ., 2014 | Hum. Genet. | T | 6 | rs35819705 | 0,025 | synonymous codon |
| Lu H., 2015 | Tumor Biol. | PRKCG | 19 | rs3745406 | 0,0074 | synonymous codon |
| Savage SA., 2013 | Nat Genet. | RNF139, TATDN1 | 8 | rs3812471 | 0,0301 | synonymous codon |
| Adiguzel M., 2016 | Indian J Exp Biol. | MMP3 | 11 | rs41380244 | 0,6686 | synonymous codon |
| Savage SA., 2013 | Nat Genet. | TRAPPC9 | 8 | rs6578061 | 0,0038 | synonymous codon |
| Ruza E., 2003 | J Pediatr Hematol Oncol | VDR | 12 | rs731236 | 0,7434 | synonymous codon |
| Savage SA., 2007 | Cancer Epidemiol Biomarkers Prev. | IGFR2 | 6 | rs894817 | 0,965 | synonymous codon |
| Kelley MJ., 2014 | Hum. Genet. | T | 6 | rs920961 | 0,59 | synonymous codon |
| Savage SA., 2007 | Cancer Epidemiol Biomarkers Prev. | IGFR2 | 6 | rs998075 | 0,0036 | synonymous codon |
| Savage SA., 2007 | Cancer Epidemiol Biomarkers Prev. | IGFR2 | 6 | rs1803989 | 0,0581 | synonymous codon, downstream variant 500B |
| Bilbao-Aldaiturriaga N., 2015 | Pediat Blood Cancer | SND1 | 7 | rs3823994 | 0,052 | synonymous codon, intron variant |
| Koshkina NV., 2007 | [J Pediatr Hematol Oncol.](http://journals.lww.com/jpho-online/pages/default.aspx?desktopMode=true) | FAS | 10 | rs2229521 | 0,0355 | synonymous codon, intron variant,nc transcript variant |
| Koshkina NV., 2007 | [J Pediatr Hematol Oncol.](http://journals.lww.com/jpho-online/pages/default.aspx?desktopMode=true) | FAS | 10 | rs2234978 | 0,7981 | synonymous codon, intron variant,nc transcript variant |
| Bilbao-Aldaiturriaga N., 2015 | Pediat Blood Cancer | CNOT1 | 16 | rs11866002 | 0,0054 | synonymous codon, nc transcript variant |
| Yang W., 2014 | Med Oncol. | ITGA3 | 17 | rs2285524 | 0,6031 | synonymous codon, nc transcript variant |
| Mirabello L., 2011 | BMC Cancer | ESR1 | 6 | rs4986934 | 0,0120 | synonymous codon, nc transcript variant |
| Walsh KM., 2016 | Carcinogenesis. | RTEL1 | 20 | rs755017 | 0,0286 | synonymous codon, nc transcript variant |
| Walsh KM., 2016 | Carcinogenesis. | TERC | 3 | rs10936599 | 0,4694 | synonymous codon, nc transcript variant, utr variant 5 prime |
| Mirabello L., 2011 | BMC Cancer | POLM | 7 | rs3218655 | 0,0267 | synonymous codon, nc transcript variant, utr variant 5 prime |
| Liu Y., 2012 | PLoS ONE | LOX | 5 | rs2278226 | 0,6927 | synonymous codon, upstream variant 2KB |
| Zhang N., 2016 | Onco Targets Ther. | WWOX | 16 | rs10220974 | 0,4394 | upstream variant 2KB |
| Ruza E., 2003 | J Pediatr Hematol Oncol | COL1A1 | 17 | rs1107946 | 0,9507 | upstream variant 2KB |
| Savage SA., 2013 | Nat Genet. | FAM91A1 | 8 | rs11987639 | 0,0378 | upstream variant 2KB |
| Tie Z., 2014 | Int J Clin Exp Pathol. | VEGFA | 6 | rs1570360 | 0,0646 | upstream variant 2KB |
| He Y., 2014 | Int Orthop. | IL1B | 2 | rs16944 | 0,0809 | upstream variant 2KB |
| Mirabello L., 2011 | BMC Cancer | NEIL2 | 8 | rs17754589 | 0,0164 | upstream variant 2KB |
| Mirabello L., 2011 | BMC Cancer | ERCC4 | 14 | rs1799797 | 0,0217 | upstream variant 2KB |
| Oliveira ID., 2007 | J Pediatr Hematol Oncol. | TNF | 6 | rs1800629 | 0,0124 | upstream variant 2KB |
| Patio-Garcia A., 2000 | J Med Genet. | TNF | 6 | rs1800629 | 0,847 | upstream variant 2KB |
| Cui Y. | Tumour Biol. | IL10 | 1 | rs1800871 | 0,306 | upstream variant 2KB |
| Cui Y. | Tumour Biol. | IL10 | 1 | rs1800872 | 0,4001 | upstream variant 2KB |
| Cui Y. | Tumour Biol. | IL10 | 1 | rs1800896 | 0,0171 | upstream variant 2KB |
| Oliveira ID., 2007 | J Pediatr Hematol Oncol. | IL10 | 1 | rs1800896 | 0,3716 | upstream variant 2KB |
| Grünewald TG., 2015 | Nat Genet. | LOC107984012 | 10 | rs1876919 | 4,5540E-03 | upstream variant 2KB |
| Grünewald TG., 2015 | Nat Genet. | EGR2 | 10 | rs1888967 | 4,4900E-04 | upstream variant 2KB |
| Grünewald TG., 2015 | Nat Genet. | EGR2 | 10 | rs224277 | 1,4030E-06 | upstream variant 2KB |
| Grünewald TG., 2015 | Nat Genet. | EGR2 | 10 | rs224278 | 1,4030E-06 | upstream variant 2KB |
| Postel-Vinay S., 2012 | Nat Genet. | EGR2 | 10 | rs224278 | 4,00E-17 | upstream variant 2KB |
| Oliveira ID., 2007 | J Pediatr Hematol Oncol. | MPO |  | rs2333227 | 0,7907 | upstream variant 2KB |
| Cui Y., 2016 | Biomarkers | MMP2 | 16 | rs243865 | 0,0016 | upstream variant 2KB |
| Savage SA., 2013 | Nat Genet. | LOC105375752 | 8 | rs2456461 | 0,0181 | upstream variant 2KB |
| Mirabello L., 2011 | BMC Cancer | CYP17A1 | 10 | rs2486758 | 0,0375 | upstream variant 2KB |
| Cui Y., 2016 | Biomarkers | MMP3 | 11 | rs3025058 | 0,3079 | upstream variant 2KB |
| Patio-Garcia A., 2000 | J Med Genet. | TNF | 6 | rs361525 | 0,012 | upstream variant 2KB |
| Patio-Garcia A., 2000 | J Med Genet. | TNF | 6 | rs361525 | 0,6 | upstream variant 2KB |
| Mirabello L., 2011 | BMC Cancer | CDKN2A | 9 | rs3731257 | 0,0239 | upstream variant 2KB |
| Cui Y., 2016 | Biomarkers | MMP9 | 20 | rs3918242 | <0.00001 | upstream variant 2KB |
| Chen Y., 2016 | Tumour Biol. | CXCL8 | 4 | rs4073 | 0,0072 | upstream variant 2KB |
| Savage SA., 2013 | Nat Genet. | TRMT12,CR93366 | 8 | rs4132855 | 0,0367 | upstream variant 2KB |
| Feng D., 2013 | Genet Test Mol Biomarkers | CTLA4 | 2 | rs4553808 | 0,4626 | upstream variant 2KB |
| Liu Y., 2011 | DNA Cell Biol. | CTLA4 | 2 | rs4553808 | 0,3297 | upstream variant 2KB |
| Feng D., 2013 | Genet Test Mol Biomarkers | CTLA4 | 2 | rs5742909 | 0,7213 | upstream variant 2KB |
| Liu Y., 2011 | DNA Cell Biol. | CTLA4 | 2 | rs5742909 | 0,1493 | upstream variant 2KB |
| Mei JW., 2016 | Int J Clin Exp Pathol | CTLA4 | 2 | rs5742909 | 0,3834 | upstream variant 2KB |
| Yang S., 2012 | Genet Test Mol Biomarkers | CTLA4 | 2 | rs5742909 | 0,348 | upstream variant 2KB |
| Savage SA., 2013 | Nat Genet. | PTPRA,GNRH2 | 20 | rs6138991 | 0,0456 | upstream variant 2KB |
| Grünewald TG., 2015 | Nat Genet. | EGR2 | 10 | rs648746 | 1,2120E-06 | upstream variant 2KB |
| Grünewald TG., 2015 | Nat Genet. | EGR2 | 10 | rs648748 | 1,2120E-06 | upstream variant 2KB |
| Mirabello L., 2011 | BMC Cancer | ERCC4 | 16 | rs6498486 | 0,0242 | upstream variant 2KB |
| Tie Z., 2014 | Int J Clin Exp Pathol. | VEGFA | 6 | rs699947 | 0,008 | upstream variant 2KB |
| Zhang HF., 2015 | Gen Mol Res. | VEGFA | 6 | rs699947 | 0,014 | upstream variant 2KB |
| Mirabello L., 2011 | BMC Cancer | CYP11B2 | 8 | rs7016924 | 0,0290 | upstream variant 2KB |
| Grünewald TG., 2015 | Nat Genet. | EGR2 | 10 | rs7076924 | 1,2120E-06 | upstream variant 2KB |
| Mei JW., 2016 | Int J Clin Exp Pathol | CTLA4 | 2 | rs733618 | 0,0302 | upstream variant 2KB |
| Walsh KM., 2016 | Carcinogenesis | ZNF208 |  | rs8105767 | 0,8788 | upstream variant 2KB |
| Mirabello L., 2011 | BMC Cancer | VEGFA | 6 | rs833061 | 0,0462 | upstream variant 2KB |
| Zhang HF., 2015 | Gen Mol Res. | VEGFA | 6 | rs833061 | 0,4921 | upstream variant 2KB |
| Hu GL. , 2015 | Genet Mol Res. | VEGFA | 6 | rs10434 | 0,4258 | utr variant 3 prime |
| Tie Z., 2014 | Int J Clin Exp Pathol. | VEGFA | 6 | rs10434 | 0,3169 | utr variant 3 prime |
| Wang Z., 2013 | Tumor Biol. | VEGFA | 6 | rs10434 | 0,7319 | utr variant 3 prime |
| Zhang G., 2015 | Gen Mol Res. | VEGFA | 6 | rs10434 | 0,5543 | utr variant 3 prime |
| Mirabello L., 2011 | BMC Cancer | MPG | 19 | rs1045001 | 0,0352 | utr variant 3 prime |
| Zhao J., 2014 | BioMed Res. Int. | ARHGAP35 | 19 | rs1052667 | <0.00001 | utr variant 3 prime |
| He M., 2013 | Tumor Biol. | COL1A1 | 17 | rs1061970 | 0,001 | utr variant 3 prime |
| Mirabello L., 2011 | BMC Cancer | MDM2 | 12 | rs1690916 | 0,0029 | utr variant 3 prime |
| Naumov VA., 2012 | Bull Exp Biol Med. | MDM2 | 12 | rs1690916 | 0,0118 | utr variant 3 prime |
| Xu H., 2016 | Med Sci Monit. | RASSF1A | 3 | rs2073497 | 0,3071 | utr variant 3 prime |
| Grünewald TG., 2015 | Nat Genet. | ADO | 10 | rs224082 | 2,3880E-05 | utr variant 3 prime |
| Savage SA., 2013 | Nat Genet. | SAMD12 | 8 | rs2514742 | 0,0398 | utr variant 3 prime |
| Savage SA., 2013 | Nat Genet. | SAMD12 | 8 | rs2514981 | 0,031 | utr variant 3 prime |
| Hu GL. , 2015 | Genet Mol Res. | VEGFA | 6 | rs3025039 | 0,0371 | utr variant 3 prime |
| Tie Z., 2014 | Int J Clin Exp Pathol. | VEGFA | 6 | rs3025039 | 0,3323 | utr variant 3 prime |
| Wang Z., 2013 | Tumor Biol. | VEGFA | 6 | rs3025039 | 0,0365 | utr variant 3 prime |
| Zhang G., 2015 | Gen Mol Res. | VEGFA | 6 | rs3025039 | 0,3016 | utr variant 3 prime |
| Zhang HF., 2015 | Gen Mol Res. | VEGFA | 6 | rs3025039 | 0,0446 | utr variant 3 prime |
| Cong Y., 2014 | Tumor Biol. | BMP2 | 20 | rs3178250 | 0,0084 | utr variant 3 prime |
| Wang J., 2013 | DNA Cell Biol. | IL12A | 3 | rs3212227 | 0,0037 | utr variant 3 prime |
| Savage SA., 2013 | Nat Genet. | FBXO32 | 8 | rs3739286 | 0,0155 | utr variant 3 prime |
| Savage SA., 2013 | Nat Genet. | ZFP41,GLI4 | 8 | rs3750213 | 0,0331 | utr variant 3 prime |
| Mirabello L., 2011 | BMC Cancer | ERCC4 | 16 | rs4781563 | 0,0330 | utr variant 3 prime |
| Mirabello L., 2011 | BMC Cancer | CD79B | 17 | rs7921 | 0,0009 | utr variant 3 prime |
| Naumov VA., 2012 | Bull Exp Biol Med. | CD79B | 17 | rs7921 | 0,6427 | utr variant 3 prime |
| Mirabello L., 2011 | BMC Cancer | TNFRSF11A | 18 | rs884205 | 0,0368 | utr variant 3 prime |
| Wang K., 2016 | Tumour Biol. | GRM4 | 6 | rs2229901 | 0,4461 | utr variant 3 prime, nc transcript variant |
| Mirabello L., 2011 | BMC Cancer | ENDOV, FLJ35220 | 17 | rs8065843 | 0,0037 | utr variant 3 prime, utr variant 5 prime |
| Savage SA., 2013 | Nat Genet. | WWOX | 16 | rs11545028 | 2,92E-02 | utr variant 5 prime |
| Hu GL. , 2015 | Genet Mol Res. | VEGFA | 6 | rs2010963 | 0,5792 | utr variant 5 prime |
| Savage SA., 2013 | Nat Genet. | MYC | 8 | rs4645948 | 0,0053 | utr variant 5 prime |
| Savage SA., 2013 | Nat Genet. | GNRH2 | 20 | rs8125955 | 0,0071 | utr variant 5 prime |
| He Y., 2014 | Int Orthop. | IL1B | 2 | rs1143627 | 0,0428 | utr variant 5 prime, upstream variant 2KB |
| Wang Z., 2013 | Tumor Biol. | VEGFA | 6 | rs2010963 | 0,688 | utr variant 5 prime, upstream variant 2KB |
| Zhang G., 2015 | Gen Mol Res. | VEGFA | 6 | rs2010963 | 0,0015 | utr variant 5 prime, upstream variant 2KB |
| Liu Y., 2012 | PLoS ONE | LOX | 5 | rs750033944 | 0,0001 | utr variant 5 prime, upstream variant 2KB |
