# Supplementary material for: Genetic susceptibility to bone and soft tissue sarcomas: a field synopsis and meta-analysis
Source: Oncotarget. 2018 Apr 6;9(26):18607–26. doi: 10.18632/oncotarget.24719 (PMC5915097; doi:10.18632/oncotarget.24719)
Supplement: Supplementary file 3 [file oncotarget-09-18607-s003.docx]

| **SNP ID** | **Gene** | **Analysis** | **Model** | **Sarcoma type** | **# studies** | **meta-analysis Ethnicity** | **OR** | **CI lower** | **CI upper** | **I 2 %** | | **P value** | **# Cases** | **# Controls** | **Ref** | **Alt** |
| --- | --- | --- | --- | --- | --- | --- | --- | --- | --- | --- | --- | --- | --- | --- | --- | --- |
| rs10208273 | GRM4 | primary | Per allele | Osteosarcoma | 2 | Mixed | 1,15 | 0,8 | 1,65 | 81 | | 0,44 | 1109 | 3507 | T | C |
| rs1042522 | TP53 | primary | per allele | Mixed | 6 | Mixed | 0,60 | 0,39 | 0,93 | 84 | | 0,02 | 788 | 950 | G | C |
| rs1042522 | TP53 | primary | dominant | Mixed | 6 | Mixed | 0,67 | 0,53 | 0,84 | 0 | | 0,0007 | 788 | 950 | G | C |
| rs1042522 | TP53 | primary | recessive | Mixed | 6 | Mixed | 0,8 | 0,58 | 1,11 | 16 | | 0,18 | 788 | 950 | G | C |
| rs1042522 | TP53 | subgroup | per allele | Osteosarcoma | 3 | Mixed | 0,47 | 0,23 | 0,95 | 93 | | 0,04 | 509 | 737 | G | C |
| rs1042522 | TP53 | subgroup | dominant | Osteosarcoma | 3 | Mixed | 0,6 | 0,43 | 0,84 | 15 | | 0,002 | 509 | 737 | G | C |
| rs1042522 | TP53 | subgroup | recessive | Osteosarcoma | 3 | Mixed | 0,88 | 0,55 | 1,38 | 52 | | 0,57 | 509 | 737 | G | C |
| rs10434 | VEGFA | primary | per allele | Osteosarcoma | 4 | Asian | 1,09 | 0,96 | 1,25 | 0 | | 0,2 | 805 | 1162 | C | T |
| rs10434 | VEGFA | primary | Recessive | Osteosarcoma | 4 | Asian | 1,16 | 0,91 | 1,49 | 0 | | 0,23 | 805 | 1162 | C | T |
| rs10434 | VEGFA | primary | Dominant | Osteosarcoma | 4 | Asian | 1,09 | 0,9 | 1,32 | 0 | | 0,36 | 805 | 1162 | C | T |
| rs10761660 | ADO, EGR2 | primary | Per allele | Ewing's Sarcoma | 2 | Caucasian | 1,39 | 1,21 | 1,60 | 0 | | <0.00001 | 744 | 4603 | T | C |
| rs1129055 | CD86 | primary | Per allele | Mixed | 2 | Asian | 0,33 | 0,11 | 1,01 | 93 | | 0,05 | 363 | 428 | A | G |
| rs1129055 | CD86 | primary | Recessive | Mixed | 2 | Asian | 0,6 | 0,41 | 0,88 | 0 | | 0,008 | 363 | 428 | A | G |
| rs1129055 | CD86 | primary | Dominant | Mixed | 2 | Asian | 0,86 | 0,47 | 1,57 | 67 | | 0,62 | 363 | 428 | A | G |
| rs11599754 | ZNF365, ADO | primary | Per allele | Ewing's Sarcoma | 2 | Caucasian | 1,48 | 1,32 | 1,66 | 0 | | <0.00001 | 744 | 4603 | T | C |
| rs11737764 | NUDT6 | primary | Dominant | Bone tumor | 2 | Caucasian | 2,12 | 1,34 | 3,37 | 0 | | 0,001 | 164 | 1522 | C | A |
| rs13181 | ERCC2 | primary | Per allele | Mixed | 3 | Mixed | 1,09 | 0,9 | 1,33 | 0 | | 0,36 | 364 | 797 | A | C |
| rs13181 | ERCC2 | primary | Recessive | Mixed | 3 | Mixed | 0,84 | 0,54 | 1,3 | 0 | | 0,43 | 364 | 797 | A | C |
| rs13181 | ERCC2 | primary | Dominant | Mixed | 3 | Mixed | 1,5 | 0,89 | 2,52 | 55 | | 0,13 | 364 | 797 | A | C |
| rs1346044 | WRN | primary | per allele | Mixed | 2 | Asian | 0,98 | 0,64 | 1,49 | 0 | | 0,91 | 609 | 1443 | A | G |
| rs1509966 | ADO, EGR2 | primary | Per allele | Ewing's Sarcoma | 2 | Caucasian | 1,58 | 1,42 | 1,77 | 0 | | <0.00001 | 744 | 4603 | A | G |
| rs1690916 | MDM2 | primary | Per allele | Ewing's Sarcoma | 2 | Caucasian | 0,62 | 0,46 | 0,83 | 0 | | 0,001 | 164 | 1522 | C | T |
| rs1695147 | MDM2 | primary | Dominant | Mixed | 2 | Mixed | 0,97 | 0,27 | 3,55 | 83 | | 0,97 | 234 | 1557 | T | G |
| rs17206779 | ADAMTS6 | primary | Per allele | Osteosarcoma | 2 | Mixed | 0,79 | 0,67 | 0,93 | 35 | | 0,004 | 1109 | 3507 | C | T |
| rs17655 | ERCC5 | primary | Per allele | Mixed | 2 | Caucasian | 1,62 | 0,88 | 2,98 | 75 | | 0,12 | 223 | 515 | G | C |
| rs17655 | ERCC5 | primary | Recessive | Mixed | 2 | Caucasian | 2,04 | 1,07 | 3,9 | 0 | | 0,03 | 223 | 515 | G | C |
| rs17655 | ERCC5 | primary | Dominant | Mixed | 2 | Caucasian | 2,59 | 0,76 | 8,84 | 84 | | 0,13 | 223 | 515 | G | C |
| rs1799793 | ERCC2 | primary | Per allele | Osteosarcoma | 2 | Mixed | 0,75 | 0,58 | 0,97 | 23 | | 0,03 | 271 | 532 | G | A |
| rs1799793 | ERCC2 | primary | Recessive | Osteosarcoma | 2 | Mixed | 0,79 | 0,56 | 1,12 | 0 | | 0,18 | 271 | 532 | G | A |
| rs1799793 | ERCC2 | primary | Dominant | Osteosarcoma | 2 | Mixed | 0,63 | 0,44 | 89 | 0 | | 0,009 | 271 | 532 | G | A |
| rs1800469 | B9D2, TGFB1 | primary | per allele | Osteosarcoma | 2 | Asian | 1,02 | 0,83 | 1,27 | 0 | | 0,83 | 326 | 352 | C | T |
| rs1800469 | B9D2, TGFB1 | primary | Recessive | Osteosarcoma | 2 | Asian | 1,13 | 0,79 | 1,62 | 0 | | 0,5 | 326 | 352 | C | T |
| rs1800469 | B9D2, TGFB1 | primary | Dominant | Osteosarcoma | 2 | Asian | 0,93 | 0,62 | 1,41 | 32 | | 0,74 | 326 | 352 | C | T |
| rs1800629 | TNF | primary | per allele | Mixed | 2 | Mixed | 1,21 | 0,46 | 3,17 | 83 | | 0,7 | 190 | 271 | G | A |
| rs1800629 | TNF | subgroup | Per allele | Osteosarcoma | 2 | Mixed | 1,39 | 0,68 | 2,87 | 65 | | 0,37 | 143 | 271 | G | A |
| rs1800795 | IL6 | primary | Per allele | Osteosarcoma | 2 | Mixed | 1,21 | 0,57 | 2,56 | 88 | | 0,63 | 286 | 366 | G | C |
| rs1800795 | IL6 | primary | Recessive | Osteosarcoma | 2 | Mixed | 1,3 | 0,71 | 2,39 | 56 | | 0,39 | 286 | 366 | G | C |
| rs1800795 | IL6 | primary | Dominant | Osteosarcoma | 2 | Mixed | 0,95 | 0,21 | 4,42 | 89 | | 0,95 | 286 | 366 | G | C |
| rs1800896 | IL10 | primary | Per allele | Osteosarcoma | 2 | Mixed | 1,33 | 1,06 | 1,66 | 0 | | 0,01 | 340 | 420 | A | G |
| rs1800896 | IL10 | primary | Recessive | Osteosarcoma | 2 | Mixed | 1,37 | 0,78 | 2,42 | 56 | | 0,28 | 340 | 420 | A | G |
| rs1800896 | IL10 | primary | Dominant | Osteosarcoma | 2 | Mixed | 1,51 | 0,86 | 2,64 | 34 | | 0,15 | 340 | 420 | A | G |
| rs1848797 | ADO, EGR2 | primary | Per allele | Ewing's Sarcoma | 2 | Caucasian | 1,57 | 1,40 | 1,77 | 0 | | <0.00001 | 744 | 4603 | G | A |
| rs1906953 | GRM4 | primary | Per allele | Osteosarcoma | 3 | Mixed | 0,9 | 0,48 | 1,71 | 95 | | 0,76 | 1235 | 3675 | G | A |
| rs2010963 | VEGFA | primary | Per allele | Osteosarcoma | 2 | Asian | 1 | 0,83 | 1,2 | 0 | | 0,96 | 460 | 472 | G | C |
| rs2010963 | VEGFA | primary | Recessive | Osteosarcoma | 2 | Asian | 0,93 | 0,66 | 1,33 | 0 | | 0,7 | 460 | 472 | G | C |
| rs2010963 | VEGFA | primary | Dominant | Osteosarcoma | 2 | Asian | 1,03 | 0,79 | 1,35 | 0 | | 0,83 | 460 | 472 | G | C |
| rs2242245 | PRKCG | primary | per allele | Osteosarcoma | 2 | Asian | 1,08 | 0,92 | 1,28 | 0 | | 0,35 | 998 | 998 | A | C |
| rs2242245 | PRKCG | primary | Recessive | Osteosarcoma | 2 | Asian | 1,41 | 0,9 | 2,23 | 0 | | 0,14 | 998 | 998 | A | C |
| rs2242245 | PRKCG | primary | Dominant | Osteosarcoma | 2 | Asian | 1,04 | 0,86 | 1,27 | 0 | | 0,66 | 998 | 998 | A | C |
| rs224278 | EGR2 | primary | per allele | Ewing's Sarcoma | 2 | Caucasian | 1,73 | 1,49 | 2,02 | 0 | | <0.00001 | 744 | 4603 | T | C |
| rs224292 | ADO, EGR2 | primary | Per allele | Ewing's Sarcoma | 2 | Caucasian | 1,67 | 1,42 | 1,96 | 0 | | <0.00001 | 744 | 4603 | A | G |
| rs2279744 | MDM2 | primary | per allele | mixed | 4 | Mixed | 1,36 | 1,06 | 1,76 | 26 | | 0,02 | 448 | 563 | T | G |
| rs2279744 | MDM2 | primary | Recessive | mixed | 4 | Mixed | 1,58 | 1,03 | 2,42 | 20 | | 0,04 | 448 | 563 | T | G |
| rs2279744 | MDM2 | primary | Dominant | mixed | 4 | Mixed | 1,55 | 1,05 | 2,29 | 36 | | 0,03 | 448 | 563 | T | G |
| rs2301291 | EWSR1 | primary | per allele | Ewing's Sarcoma | 2 | Mixed | 0,94 | 0,7 | 1,27 | 0 | | 0,7 | 158 | 398 | G | A |
| rs2301291 | EWSR1 | primary | Recessive | Ewing's Sarcoma | 2 | Mixed | 1,26 | 0,56 | 2,84 | 35 | | 0,58 | 158 | 398 | G | A |
| rs2301291 | EWSR1 | primary | Dominant | Ewing's Sarcoma | 2 | Mixed | 0,86 | 0,58 | 1,26 | 0 | | 0,43 | 158 | 398 | G | A |
| rs2305089 | T | primary | Per allele | Chordoma | 3 | Mixed | 2,87 | 1,35 | 6,08 | 86 | | 0,006 | 228 | 1001 | G | A |
| rs2305089 | T | primary | Recessive | Chordoma | 2 | Mixed | 4,16 | 1,21 | 14,25 | 82 | | 0,02 | 125 | 841 | G | A |
| rs2305089 | T | primary | Dominant | Chordoma | 2 | Mixed | 2,49 | 0,56 | 10,98 | 68 | | 0,23 | 125 | 841 | G | A |
| rs231775 | CTLA4 | primary | per allele | mixed | 4 | Asian | 1,36 | 1,20 | 1,54 | 0 | | <0.00001 | 1003 | 1162 | G | A |
| rs231775 | CTLA4 | primary | Recessive | mixed | 4 | Asian | 2,00 | 1,53 | 2,62 | 0 | | <0.00001 | 1003 | 1162 | G | A |
| rs231775 | CTLA4 | primary | Dominant | mixed | 4 | Asian | 1,35 | 1,14 | 1,61 | 0 | | 0,0007 | 1003 | 1162 | G | A |
| rs231775 | CTLA4 | subgroup | per allele | Ewing's Sarcoma | 2 | Asian | 1,36 | 1,15 | 1,61 | 0 | | 0,0003 | 531 | 664 | G | A |
| rs231775 | CTLA4 | subgroup | recessive | Ewing's Sarcoma | 2 | Asian | 2,00 | 1,39 | 2,89 | 0 | | 0,0002 | 531 | 664 | G | A |
| rs231775 | CTLA4 | subgroup | dominant | Ewing's Sarcoma | 2 | Asian | 1,36 | 1,07 | 1,72 | 0 | | 0,01 | 531 | 664 | G | A |
| rs231775 | CTLA4 | subgroup | per allele | Osteosarcoma | 2 | Asian | 1,36 | 1,13 | 1,64 | 0 | | 0,001 | 472 | 498 | G | A |
| rs231775 | CTLA4 | subgroup | recessive | Osteosarcoma | 2 | Asian | 2,00 | 1,34 | 2,98 | 0 | | 0,0007 | 472 | 498 | G | A |
| rs231775 | CTLA4 | subgroup | dominant | Osteosarcoma | 2 | Asian | 1,35 | 1,04 | 1,75 | 0 | | 0,02 | 472 | 498 | G | A |
| rs3025039 | VEGFA | primary | per allele | Osteosarcoma | 5 | Asian | 1,28 | 1,12 | 1,47 | 0 | | 0,0004 | 987 | 1344 | C | T |
| rs3025039 | VEGFA | primary | Recessive | Osteosarcoma | 5 | Asian | 1,65 | 1,19 | 2,27 | 6 | | 0,002 | 987 | 1344 | C | T |
| rs3025039 | VEGFA | primary | Dominant | Osteosarcoma | 5 | Asian | 1,24 | 1,04 | 1,47 | 0 | | 0,02 | 987 | 1344 | C | T |
| rs3087243 | CTLA4 | primary | per allele | mixed | 2 | Asian | 1,01 | 0,82 | 1,24 | 0 | | 0,95 | 575 | 644 | A | G |
| rs3087243 | CTLA4 | primary | recessive | mixed | 2 | Asian | 1,12 | 0,63 | 1,98 | 0 | | 0,7 | 575 | 644 | A | G |
| rs3087243 | CTLA4 | primary | dominant | mixed | 2 | Asian | 0,99 | 0,78 | 1,26 | 0 | | 0,93 | 575 | 644 | A | G |
| rs3761243 | GNRH2 | primary | Per allele | Osteosarcoma | 2 | Caucasian | 1,03 | 0,41 | 2,57 | 90 | | 0,96 | 164 | 1522 | A | C |
| rs454006 | PRKCG | primary | Per allele | Osteosarcoma | 2 | Asian | 1,35 | 1,18 | 1,54 | 0 | | <0.0001 | 998 | 998 | T | C |
| rs454006 | PRKCG | primary | recessive | Osteosarcoma | 2 | Asian | 1,99 | 1,54 | 2,58 | 0 | | <0.0001 | 998 | 998 | T | C |
| rs454006 | PRKCG | primary | dominant | Osteosarcoma | 2 | Asian | 1,20 | 0,99 | 1,46 | 15 | | 0,06 | 998 | 998 | T | C |
| rs4553808 | CTLA4 | primary | per allele | mixed | 2 | Asian | 1,13 | 0,92 | 1,40 | 0 | | 0,23 | 575 | 644 | A | G |
| rs4553808 | CTLA4 | primary | recessive | mixed | 2 | Asian | 1,31 | 0,76 | 2,24 | 0 | | 0,33 | 575 | 644 | A | G |
| rs4553808 | CTLA4 | primary | dominant | mixed | 2 | Asian | 1,13 | 0,88 | 1,44 | 0 | | 0,33 | 575 | 644 | A | G |
| rs4820803 | EWSR1 | primary | Per allele | Ewing's Sarcoma | 2 | Mixed | 1,1 | 0,76 | 1,59 | 0 | | 0,61 | 151 | 397 | G | C |
| rs4820803 | EWSR1 | primary | Recessive | Ewing's Sarcoma | 2 | Mixed | 0,76 | 0,22 | 2,67 | 0 | | 0,67 | 151 | 397 | G | C |
| rs4820803 | EWSR1 | primary | Dominant | Ewing's Sarcoma | 2 | Mixed | 1,2 | 0,78 | 1,82 | 0 | | 0,4 | 151 | 397 | G | C |
| rs5742909 | CTLA4 | primary | per allele | mixed | 4 | Asian | 1,07 | 0,90 | 1,28 | 6 | | 0,44 | 895 | 1066 | C | T |
| rs5742909 | CTLA4 | primary | recessive | mixed | 4 | Asian | 1,33 | 0,68 | 2,62 | 29 | | 0,41 | 895 | 1066 | C | T |
| rs5742909 | CTLA4 | primary | dominant | mixed | 4 | Asian | 1,05 | 0,87 | 1,27 | 0 | | 0,61 | 895 | 1066 | C | T |
| rs5742909 | CTLA4 | Subgroup | per allele | Ewing's Sarcoma | 2 | Asian | 1,04 | 0,84 | 1,29 | 0 | | 0,72 | 531 | 664 | C | T |
| rs5742909 | CTLA4 | Subgroup | recessive | Ewing's Sarcoma | 2 | Asian | 1,06 | 0,39 | 2,91 | 50 | | 0,91 | 531 | 664 | C | T |
| rs5742909 | CTLA4 | Subgroup | dominant | Ewing's Sarcoma | 2 | Asian | 1,05 | 0,82 | 1,34 | 0 | | 0,72 | 531 | 664 | C | T |
| rs5742909 | CTLA4 | Subgroup | per allele | Osteocarcoma | 2 | Asian | 1,05 | 0,67 | 1,65 | 52 | | 0,83 | 364 | 402 | C | T |
| rs5742909 | CTLA4 | Subgroup | recessive | Osteocarcoma | 2 | Asian | 1,95 | 0,80 | 4,78 | 3 | | 0,14 | 364 | 402 | C | T |
| rs5742909 | CTLA4 | Subgroup | dominant | Osteocarcoma | 2 | Asian | 1,03 | 0,69 | 1,53 | 29 | | 0,9 | 364 | 402 | C | T |
| rs6479860 | LOC107984012, NRBF2 | primary | Per allele | Ewing's Sarcoma | 2 | Caucasian | 1,79 | 1,36 | 2,34 | 66 | | <0.0001 | 744 | 4603 | C | T |
| rs6599400 | FGFR3 | primary | Per allele | Osteocarcoma | 2 | Caucasian | 1,53 | 1,19 | 1,97 | 0 | | 0,001 | 164 | 1522 | C | A |
| rs699947 | VEGFA | primary | Per allele | Osteosarcoma | 2 | Asian | 1,46 | 1,19 | 1,79 | 0 | | 0,0003 | 347 | 512 | C | A |
| rs699947 | VEGFA | primary | Recessive | Osteosarcoma | 2 | Asian | 1,73 | 1,17 | 2,55 | 0 | | 0,006 | 347 | 512 | C | A |
| rs699947 | VEGFA | primary | Dominant | Osteosarcoma | 2 | Asian | 1,51 | 1,14 | 2 | 0 | | 0,004 | 347 | 512 | C | A |
| rs7591996 | LOC105373401 | primary | Per allele | Osteosarcoma | 2 | Mixed | 1,28 | 1,02 | 1,61 | 53 | | 0,03 | 1109 | 3507 | A | C |
| rs7921 | CD79B | primary | Per allele | Mixed | 2 | Caucasian | 0,66 | 0,39 | 1,11 | 61 | | 0,12 | 164 | 1522 | C | T |
| rs7956547 | IGF1 | primary | Per allele | Osteosarcoma | 2 | Caucasian | 0,69 | 0,39 | 1,24 | 68 | | 0,22 | 164 | 1522 | T | C |
| rs8103851 | PRKCG | primary | per allele | Osteosarcoma | 2 | Asian | 0,95 | 0,80 | 1,13 | 47 | | 0,55 | 998 | 998 | C | G |
| rs8103851 | PRKCG | primary | recessive | Osteosarcoma | 2 | Asian | 0,91 | 0,66 | 1,26 | 54 | | 0,58 | 998 | 998 | C | G |
| rs8103851 | PRKCG | primary | dominant | Osteosarcoma | 2 | Asian | 0,94 | 0,78 | 1,14 | 0 | | 0,54 | 998 | 998 | C | G |
| rs820196 | RECQL5 | primary | Per allele | Osteosarcoma | 2 | Asian | 1,03 | 0,57 | 1,87 | 88 | | 0,93 | 397 | 441 | T | C |
| rs820196 | RECQL5 | primary | Recessive | Osteosarcoma | 2 | Asian | 2,15 | 1,41 | 3,29 | 0 | | 0,0004 | 397 | 441 | T | C |
| rs820196 | RECQL5 | primary | Dominant | Osteosarcoma | 2 | Asian | 1,49 | 1,12 | 1,98 | 0 | | 0,006 | 397 | 441 | T | C |
| rs833061 | VEGFA | primary | Per allele | Osteosarcoma | 2 | Mixed | 1,23 | 0,99 | 1,52 | 0 | | 0,06 | 278 | 1608 | T | C |
| rs861539 | XRCC3, KLC1 | primary | Per allele | Osteosarcoma | 2 | Asian | 1,57 | 1,25 | 1,97 | 0 | | 0,0001 | 288 | 440 | C | T |
| rs861539 | XRCC3, KLC1 | primary | Recessive | Osteosarcoma | 2 | Asian | 2,23 | 1,4 | 3,57 | 0 | | 0,0008 | 288 | 440 | C | T |
| rs861539 | XRCC3, KLC1 | primary | Dominant | Osteosarcoma | 2 | Asian | 1,57 | 1,16 | 2,13 | 0 | | 0,003 | 288 | 440 | C | T |
| rs944684 | LOC107984012 | primary | Per allele | Ewing's Sarcoma | 2 | Caucasian | 1,73 | 1,40 | 2,14 | 49 | | <0.00001 | 744 | 4603 | C | T |
| rs9633562 | EGR2, LOC107984012 | primary | Per allele | Ewing's Sarcoma | 2 | Caucasian | 1,46 | 1,29 | 1,65 | 0 | | <0.00001 | 744 | 4603 | A | C |
| rs9895829 | TP53 | primary | Per allele | Osteosarcoma | 2 | Mixed | 0,98 | 0,66 | 1,45 | 0 | | 0,93 | 311 | 491 | A | G |
| rs9895829 | TP53 | primary | Dominant | Osteosarcoma | 2 | Mixed | 0,98 | 0,65 | 1,48 | 0 | | 0,93 | 311 | 491 | A | G |
| deletion | GSTM1 | primary | Recessive | Mixed | 5 | Mixed | 1,2 | 0,72 | 2,01 | 72 | | 0,48 | 407 | 1038 | non-null | null |
| deletion | GSTT1 | primary | Recessive | Mixed | 4 | Mixed | 1,32 | 1,01 | 1,73 | 4 | | 0,04 | 355 | 938 | non-null | null |
|  |  |  |  |  |  |  |  |  |  |  | |  |  |  |  |  |
| **Sensitivity by ethnicity** | |  |  |  |  |  |  |  |  |  | |  |  |  |  |  |
| rs1042522 | TP53 | sensitivity by ethnicity | per allele | Osteosarcoma | 2 | Caucasian | 0,36 | 0,09 | 1,42 | 95 | | 0,14 | 299 | 317 | G | C |
| rs1042522 | TP53 | sensitivity by ethnicity | dominant | Osteosarcoma | 2 | Caucasian | 0,4 | 0,11 | 1,43 | 47 | | 0,16 | 299 | 317 | G | C |
| rs1042522 | TP53 | sensitivity by ethnicity | recessive | Osteosarcoma | 2 | Caucasian | 1,16 | 0,72 | 1,85 | | 0 | 0,54 | 299 | 317 | G | C |
| rs13181 | ERCC2 | sensitivity by ethnicity | Per allele | Mixed | 2 | Caucasian | 1,02 | 0,81 | 1,29 | 0 | | 0,85 | 223 | 303 | A | C |
| rs13181 | ERCC2 | sensitivity by ethnicity | Recessive | Mixed | 2 | Caucasian | 0,62 | 0,36 | 1,07 | 0 | | 0,08 | 223 | 303 | A | C |
| rs13181 | ERCC2 | sensitivity by ethnicity | Dominant | Mixed | 2 | Caucasian | 1,76 | 0,82 | 3,81 | 63 | | 0,15 | 223 | 303 | A | C |
| rs1906953 | GRM4 | sensitivity by ethnicity | Per allele | Osteosarcoma | 2 | Asian | 0,68 | 0,55 | 0,84 | 0 | | 0,0004 | 294 | 384 | G | A |
| rs2279744 | MDM2 | sensitivity by ethnicity | per allele | mixed | 2 | Caucasian | 1,25 | 0,81 | 1,94 | 70 | | 0,31 | 269 | 435 | T | G |
| rs2279744 | MDM2 | sensitivity by ethnicity | Recessive | mixed | 2 | Caucasian | 1,47 | 0,71 | 3,03 | 62 | | 0,29 | 269 | 435 | T | G |
| rs2279744 | MDM2 | sensitivity by ethnicity | Dominant | mixed | 2 | Caucasian | 1,3 | 0,85 | 1,99 | 35 | | 0,23 | 269 | 435 | T | G |
| rs2305089 | T | sensitivity by ethnicity | Per allele | Chordoma | 2 | Caucasian | 3,91 | 2,4 | 6,38 | 47 | | <0.00001 | 163 | 881 | G | A |
| deletion | GSTT1 | sensitivity by ethnicity | Recessive | bone cancer | 2 | Asian | 1,38 | 0,84 | 2,29 | 62 | | 0,21 | 263 | 478 | non-null | null |
| deletion | GSTM1 | sensitivity by ethnicity | Recessive | bone cancer | 3 | Asian | 1,69 | 1,02 | 2,81 | 66 | | 0,04 | 315 | 578 | non-null | null |
