# Supplementary material for: Genetic susceptibility to bone and soft tissue sarcomas: a field synopsis and meta-analysis
Source: Oncotarget. 2018 Apr 6;9(26):18607–26. doi: 10.18632/oncotarget.24719 (PMC5915097; doi:10.18632/oncotarget.24719)
Supplement: Supplementary file 4 [file oncotarget-09-18607-s004.doc]

| **Reference** | **Gene** | **Nucleotide change** | **Aminoacid change** | **Polymorphism ID** | **Restriction enzyme** |
| --- | --- | --- | --- | --- | --- |
| Wang J., 2012 | CD86 | +1057 G>A |  | rs1129055 | BbvI GCAGCNNNNNNNNNNNN |
| Ruza E., 2003 | COL1A1 | M=C  m=A |  | rs1107946 | MscI TGGCCA [?]  BsaI GGTCTC |
| He J., 2014 | CTLA4 | c.75 G>C |  | rs16840275 | HpaII CCGG |
| He J., 2014 | CTLA4 | c.326 G>A |  | rs144988077 | TaqI TCGA |
| Feng D., 2013  Liu Y., 2011 | CTLA4 | -1661 A>G |  | rs4553808 | MseI TTAA |
| Feng D., 2013  Liu Y., 2011 | CTLA4 | 60 A>G |  | rs3087243 | HpyCH4 IV ACGT |
| Feng D., 2013  Liu Y., 2011 | CTLA4 | +49 A>G |  | rs231775 | Eco91I GGTNACC |
| Feng D., 2013  Liu Y., 2011  Mei JW., 2016 | CTLA4 | -318 C>T |  | rs5742909 | MseI TTAA |
| Mei JW., 2016 | CTLA4 | -1722 T>C |  | rs733618 | BbvI GCAGCNNNNNNNNNNNN |
| Chen Y., 2016 | CXCL8 / IL8 | -251 T>A |  | rs4073 | MfeI CAATTG |
| Chen Y., 2016 | CXCL8 / IL8 | +781 C>T |  | rs2227306 | ? |
| Li L., 2016 | CYP1A1 | 4889 A>G | Ile462Val | rs1048943 | MspI CCGG |
| Ruza E., 2003 | ER | P=T  p=C |  | rs2234693 | PvuII CAGCTG3 |
| Ruza E., 2003 | ER | X=A  x=G |  | rs9340799 | XbaI TCTAGA |
| Le Morvan V., 2006 | ERCC2 / XPG |  | Asp1104His | rs17655 | MboI ATCC |
| Le Morvan V., 2006 | ERCC5 / XPD |  | Lys751Gln | rs13181 | PstI CTGCAG  EarI CTCTTC |
| Koshkina NV., 2007 | FAS | -1377 G>A |  | rs2234767 | BsrGI TGTACA |
| Koshkina NV., 2007 | FAS | -670 A>G |  | rs1800682 | MvaI CCWGG |
| Koshkina NV., 2007 | FAS | Exon3 18272 A>G |  | rs2229521 | BsrGI TGTACA |
| Koshkina NV., 2007 | FAS | Exon7 22628 C>T |  | rs2234978 | DraI TTTAAA |
| Saito T., 2000 | GJA4 / CX37 |  | Pro319Ser | rs1764391 |  |
| Barnette P., 2004 | GSTM1 | A/B | Lys173Asn  Lys=G  Asn=C | rs1065411 |  |
| Barnette P., 2004  Salinas-Souza C., 2010 | GSTM3 | A/B | B= CTC deletion | rs58210492 | MnlI CCTCNNNNNNN |
| Barnette P., 2004  Qu WR., 2016 | GSTP1 | A/B/C/D | Ile =A  Val= G  Ile104Val | rs1695 |  |
| Barnette P., 2004 | GSTP1 | A/B/C/D | Ala114Val  C=Ala  T=Val | rs1138272 |  |
| Gloudemans T. 1993 | IGF2 | a=C; b=T |  | rs680 | AvaII GGWCC |
| Cui Y., 2015  Oliveira ID., 2007 | IL10 | -1082 A>G |  | rs1800896 | NlaIII CATG |
| Cui Y., 2015 | IL10 | -819 C>T |  | rs1800871 | BglII AGATCT |
| Cui Y., 2015 | IL10 | -592 A>C |  | rs1800872 | RsaI GTAC |
| He Y., 2014 | IL1B | −31 T>C |  | rs1143627 | AluI AGCT |
| He Y., 2014 | IL1B | −511 C>T |  | rs16944 | DdeI CTNAG |
| He Y., 2014 | IL1B | +3954 C>T |  | rs1143634 | TaqI TCGA |
| Oliveira ID., 2007  Qi Y., 2016 | IL6 | -174 G>C |  | rs1800795 | NlaIII CATG |
| Qi Y., 2016 | IL6 | -572 G>C |  | rs1800796 | MbiI CCGCTC |
| Tang YJ., 2014 | IL27 | -964 A>G |  | rs153109 | XhoI CTCGAG |
| Tang YJ., 2014 | IL27 | 2905 T>G |  | rs17855750 | BstuI CGCG |
| Tang YJ., 2014 | IL27 | 4730 T>C |  | rs181206 | Fau I CCCGC |
| Liu Y., 2012 | LOX | -22 G>T |  | rs750033944 |  |
| Liu Y., 2012 | LOX | 225 C>G |  | rs2278226 | SacII CCGCGG |
| Liu Y., 2012 | LOX | 473 G>A |  | rs1800449 | PstI CTGCAG |
| Oliveira ID., 2007 | LTA / TNF-beta | +252 A>G |  | rs909253 | NcoI CCATGG |
| Nakayama R., 2008 | MBD4 | 1212 G>A | Glu346Lys | rs140693 |  |
| Alhopuro P, 2005  Ito M., 2010  Thurow HS., 2013  Toffoli G., 2009 | MDM2 | 309 T>G |  | rs2279744 | MspA1I CMGCKG |
| Hu Z., 2015 | MDM2 | c.346 G>A |  | rs11177386 |  |
| He J., 2013 | MDM2 | c.44C>T |  | rs201821879 | AvaII GGWCC |
| He J., 2013 | MDM2 | c.1002T>C |  | rs199812774 | TspEI AATT |
| Lv H., 2014 | miR-34a | G>C |  | rs2910164 |  |
| Lv H., 2014 | miR-34a | G>A |  | rs72631823 |  |
| Adiguzel M., 2016 | MMP-3 |  | E45K | rs679620 | TaqI TCGA |
| Adiguzel M., 2016 | MMP-3 |  | T102T | rs41380244 | AclI AACGTT |
| Oliveira ID., 2007 | MPO | -463 A>G |  | rs2333227 | AcII CCGC |
| Nakayama R., 2008 | MSH6 | 203 G>A | Gly39Glu | rs1042821 |  |
| Ozger H., 2008 | MTHFR | C677T |  | rs1801133 | HinfI GANTC |
| Aoyama T., 2002 | NFATC2 /NFAT1 | A1557T | His446Arg | rs12479626 |  |
| Aoyama T., 2002 | NFATC2 / NFAT1 | C2859T | Pro850Leu | rs55980737 |  |
| Nakayama R., 2008 | PARP / ADPRT | 2978 A>G | Lys940Arg | rs1136471 |  |
| Oliveira ID., 2007 | PECAM-1 / CD31 | +125 C>G | Val125Leu | rs281865545 | AluI AGCT |
| Ergen A., 2010 | PON1 |  | Gln192Arg | rs662 | AlwI GGATCNNNNN |
| Ergen A., 2010 | PON1 |  | Leu55Met | rs854560 | Hsp192II CATG |
| Grochola LF., 2009 | PPP2R5E | Epsilon-2SNP |  | rs11158941 |  |
| Nakayama R., 2008 | REV1 | 1330 A>G | Asn373Ser | rs3087399 |  |
| Wu Y., 2015 | TGFB1 | 29 T>C |  | rs1800470 |  |
| Wu Y., 2015 | TGFB1 | 509 C>T |  | rs1800469 |  |
| Wu Y., 2015 | TGFB1 | 869 T>C |  | rs1800473 |  |
| Hu YS., 2010 | TGFBR1 | TGFBR1*6A |  | rs11466445 |  |
| Hu YS., 2011 | TGFBR1 | Int7G24A |  | rs334354 | Bsr1 ACTGGN |
| Oliveira ID., 2007  Patio-Garcia A., 2000 | TNFA / TNF alpha | -308 G>A |  | rs1800629 | NcoI CCATGG |
| Patio-Garcia A., 2000 | TNFA/ TNF alpha | -238 G>A |  | rs361525 |  |
| Almeida PSR., 2008  Ito M., 2010  Thurow HS., 2013  Toffoli G., 2009 | TP53 | 466 G>C | Arg72Pro | rs1042522 |  |
| Savage SA., 2007 | TP53-52 | E3346_28 |  | rs17880604 |  |
| Savage SA., 2007 | TP53-71 | E3355_424 |  | rs17887200 |  |
| Ruza E., 2003 | VDR | TaqI  T=C  t=T |  | rs731236 | TaqI TCGA |
| Ruza E., 2003 | VDR | FokI  F=T  f=C |  | rs2228570 | FokI GGATGNNNNNNNNNNNNN |
| Ruza E., 2003 | VDR | ApaI  A=C  a=A |  | rs7975232 | ApaI GGGCCC |
| Hu GL., 2015 Wang Z., 2013  Zhang G., 2015 | VEGFA | -634 G>C |  | rs2010963 | BsmFI GGGAC [?] |
| Hu GL., 2015 Wang Z., 2013 Zhang G., 2015 Zhang HF., 2015 | VEGFA | +936 C>T |  | rs3025039 | NlaIII CATG |
| Hu GL., 2015  Wang Z., 2013 Zhang G., 2015 | VEGFA | +1612 G>A |  | rs10434 | MnII CCTCNNNNNN_N |
| Zhang HF., 2015 | VEGFA | -2578 C>A |  | rs699947 | BglII AGATCT [?] |
| Zhang HF., 2015 | VEGFA | -460 T>C |  | rs833061 | [?] |
| Nakayama R., 2008 | WRN | 4330 T>C | Cys1367Arg | rs1346044 |  |
| Nakayama R., 2008 | XRCC1 | 685 C>T | Arg194Trp | rs1799782 |  |
| Guo J., 2015  Yang LB., 2015 | XRCC3 |  | Thr241Met | rs861539 | NlaIII CATG |
